# Mechanistic Insight Into the AuCN Catalyzed Annulation Reaction of Salicylaldehyde and Aryl Acetylene: Cyanide Ion Promoted Umpolung Hydroacylation/Intramolecular Oxa-Michael Addition Mechanism

**DOI:** 10.3389/fchem.2019.00557

**Published:** 2019-08-06

**Authors:** Manyi Yang, Guoqiang Wang, Jingxiang Zou, Shuhua Li

**Affiliations:** Key Laboratory of Mesoscopic Chemistry of Ministry of Education, School of Chemistry and Chemical Engineering, Institute of Theoretical and Computational Chemistry, Nanjing University, Nanjing, China

**Keywords:** DFT, MD/CD, cyanide ion, umpolung, hydroacylation

## Abstract

The detailed mechanism of the AuCN-catalyzed annulation of salicylaldehyde (SA) and phenyl acetylene leading to isoflavanone-type complexes has been investigated via density functional theory (DFT) calculations. Reaction pathways and possible stationary points are obtained with the combined molecular dynamics and coordinate driving (MD/CD) method. Our calculations reveal that the cyanide ion promoted umpolung hydroacylation/intramolecular oxa-Michael addition mechanism is more favorable than the Au(I)/Au(III) redox mechanism proposed previously. In the umpolung mechanism, the hydroxyl of SA is found to strongly stabilize the cyanide ion involved intermediates and transition states via hydrogen bond interactions, while the Au(I) ion always acts as a counter cation. The overall reaction is exergonic by 41.8 kcal/mol. The hydroacylation of phenyl acetylene is the rate-determining step and responsible for the regioselectivity with a free energy barrier of 27.3 kcal/mol. These calculated results are in qualitative accord with the experimental findings.

## Introduction

The catalytic annulation of chelating aldehydes and unsaturated hydrocarbons provides a general, highly efficient, and atom-economical strategy to synthesize complex carbocyclic and heterocyclic frameworks (Willis, 2010). Salicylaldehyde (SA) is a highly favored chelating aldehyde because of its ready availability as well as common ortho-hydroxy or alkoxy arylcarbonyl motifs in natural products and bioactive molecules. In recent years, starting from SA, various transition-metal-catalyzed C_sp2_-H activation and heterocyclization reactions have been developed to efficiently construct various important heterocycles of biologically active compounds and drug molecules (Shimizu et al., 2008; Zeng and Li, 2014; Baruah et al., 2016; Yang and Yoshikai, 2016). For example, the Rh(III)-catalyzed (Shimizu et al., 2008), Ru(II)-catalyzed (Baruah et al., 2016), Co(I)-diphosphine-catalyzed (Yang and Yoshikai, 2016) C_sp2_-H activation and annulation reactions of SA with monosubstituted and disubstituted alkyne have been reported for the synthesis of chromone derivatives.

**Scheme 1:**
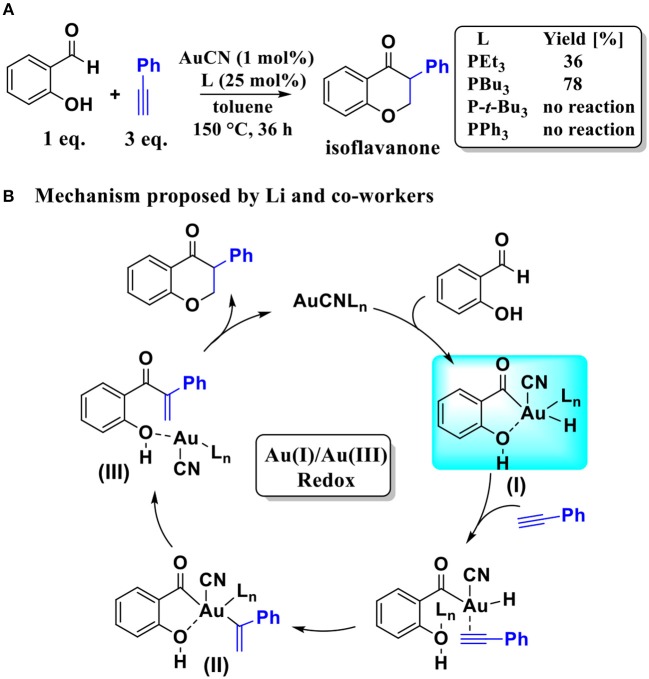
**(A)** AuCN-catalyzed annulation of salicylaldehyde with aryl acetylene; **(B)** The Au(I)/Au(III) redox mechanism proposed by Li and co-workers.

Over the last two decades, homogeneous gold catalysis has developed very rapidly and broadly in organic synthesis due to its rich chemistry and fascinating reactivity. It has emerged as a very efficient method for rapid construction of complex and highly functionalized molecules (Fukuda and Utimoto, 1991; Teles et al., 1998; Hashmi et al., 2000a,b; Yao and Li, 2004; Nguyen et al., 2006; Boorman and Larrosa, 2011; Xie et al., 2014; Dorel and Echavarren, 2015; Joost et al., 2015; Qian and Zhang, 2015; Kumar and Nevado, 2017; Akram et al., 2018; Mandal and Datta, 2018; Matsumoto et al., 2018; Wu et al., 2018; Kreuzahler et al., 2019; Mascareñas et al., 2019). Very recently, starting with SA and aryl acetylene, Li and co-workers developed an AuCN-catalyzed system (Skouta and Li, 2007) to afford the isoflavanone-type frameworks, which have many possible applications in the synthesis of isoflavanone natural products. Although the AuCN-catalyzed annulation of SA and aryl acetylene can effectively construct isoflavanone-type frameworks, the reaction conditions are very harsh. It takes place at high temperature (150°C), and over-stoichiometric amounts of alkynes (3 equivalent) are required, as shown in Scheme 1A. Moreover, the detailed mechanism of this reaction is still elusive. We believe that the elucidation of the mechanism of this reaction is helpful for designing new and milder catalytic reaction systems.

Based on the regioselectivity of the AuCN-catalyzed annulation reaction, Li and co-workers suggested an Au(I)/Au(III) redox mechanism via C_sp2_-H activation of aldehyde, as shown in Scheme 1B. This mechanism starts with an oxidative addition of the aldehyde C_sp2_-H bond leading to an acyl Au(III) hydride **I**. Then a hydrometalation step occurs, followed by a reductive elimination process and then an intramolecular conjugate addition, affording the final isoflavanone derivative and regenerating the Au(I) catalyst. Experiments indicated that the phosphine ligands had significant influence on the yield of product. As shown in Scheme 1A, triethylphosphane (PEt_3_) and tributylphosphane (PBu_3_) exhibited moderate to good efficiency (36 and 78% yield, respectively), while other alternative ligands, such as triphenyphosphane (PPh_3_) and tri-*tert*-butylphosphane (P-*t*-Bu_3_), providing only trace amounts of product.

In the present work, we carry out density functional theory (DFT) calculations to investigate the mechanism of the AuCN-catalyzed annulation reaction between SA and phenyl acetylene using AuCN as the catalyst and PEt_3_ as the model ligand. The combined molecular dynamics and coordinate driving (MD/CD) method (Yang et al., 2017b, 2018) developed by us is applied to explore reaction pathways and determine the structures of all possible stationary points along reaction pathways. This method is a cost-effective theoretical tool for automatically searching low-energy reaction pathways for relatively large chemical reactions in both gas phase and solution. Our calculations indicate that the pathway initiated by the oxidative addition of aldehyde Csp_2_-H bond toward Au (I) center could be excluded, due to its high activation barrier. Instead, our calculations suggest a cyanide ion promoted umpolung hydroacylation/intramolecular oxa-Michael addition mechanism for this reaction.

## Computational Details

Geometries of all stationary points, including minimum structures (reactants, intermediates, and products) and transition states (TSs), were fully optimized by the DFT calculations using the B3PW91 functional (Lee et al., 1988) with Grimme's dispersion correction (GD3BJ) (Grimme et al., 2010). The SDD (Figgen et al., 2005) basis set and associated effective core potentials were used for Au, 6-31+G(2d) basis set for P and 6-31+G(d) for other atoms (BS1). To obtain more accurate electronic energies, single-point energies of the optimized stationary points were recalculated (with the same functional) by employing the SDD basis set for Au and the 6-311+G(2d,p) basis set for all other atoms (BS2). The C-PCM Solvation Model (Cossi et al., 2003) was used as the implicit solvation model with the toluene as the solvent. For each TS, the intrinsic reaction coordinate (IRC) (Gonzalez and Schlegel, 1989) analysis was performed to verify whether the TS truly connects the reactant and the product. The Gibbs free energies were calculated at T = 423 K and 1.0 atm in toluene (corresponding to the experimental conditions) by the way described previously (Zeng and Li, 2011), in which the entropy of translational movement was evaluated with the method developed by Mammen et al. (1998). All calculations were performed with the Gaussian16 (Frisch et al., 2016) software package.

The MD/CD method implemented in the automated design of chemical reaction (ADCR) program (Yang et al., 2017a) is applied to locate all the stationary points along the most probable reaction pathways. Since the system is very large and the resulting lower-energy pathways will be further verified with more accurate calculations, we performed MD/CD calculations with the B3PW91 functional (also with GD3BJ) at a smaller basis set. In this basis set, the effective core potential LanL2DZ was used for Au and the 6-31G basis set was used for other atoms (BS3). The C-PCM model was used as the implicit solvation model with toluene as the solvent. In the MD/CD calculation, the MD simulations were skipped for all intermediate structures (due to their relatively rigid structures) and the energy cutoff 45 kcal/mol was used.

Activation free energy barriers (ΔG^≠^) used in this work are defined as the free energy difference between the TS and the most stable points (initial reactants and intermediates) along the energy profile. Given that the formation of the PEt_3_AuCN complex (from the initial AuCN catalyst and the PEt_3_ ligand) is highly exergonic, free energies discussed in this work are with respect to PEt_3_AuCN.

## Results and Discussions

### Au(I)/Au(III) Redox Mechanism

**Figure 1:**
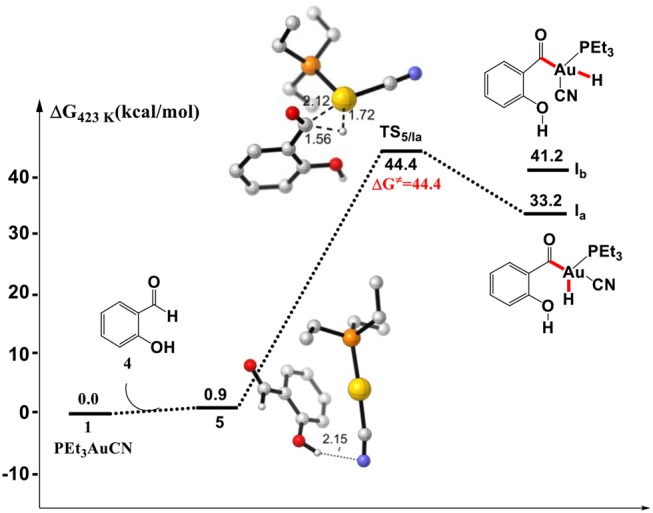
Gibbs free energy profile of the oxidative addition process. Relative free energies calculated at 423 K and 1 atm (with respect to separated reactants, in kcal/mol). All listed distances are in Å.

Initially, we investigated the “intuitive” Au(I)/Au(III) redox mechanism, which involves the direct oxidative addition of aldehyde C_sp2_-H bond to PEt_3_AuCN complex **1**. As shown in Figure 1, the formation of a hydrogen bond between cyano group of PEt_3_AuCN and the hydroxyl of SA in the complex **5** is endergonic by 0.9 kcal/mol. However, the direct oxidative addition of the formyl C_sp2_-H bond of SA toward the Au(I) center via transition state **TS**_5/Ia_ to form the Au(III) intermediate **Ia** is required to overcome a high activation barrier of 44.4 kcal/mol (relative to reactants), which is inconsistent with the experimental conditions that the reaction occurs at 150°C. Therefore, instead of Au(I)/Au(III) redox catalytic cycle proposed previously, there might be an energetically more favorable pathway.

### The Cyanide Ion Promoted Umpolung Pathway

#### Determination of the Active Catalyst

**Scheme 2:**
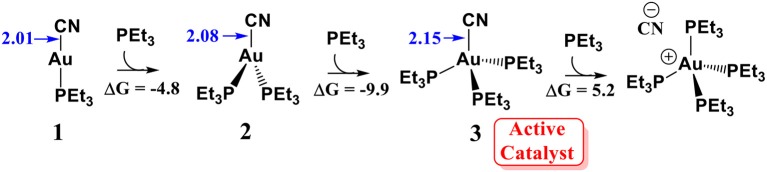
Reaction energies (ΔG) for the association of PEt_3_AuCN with PEt_3_ ligand. All energies are in kcal/mol; All bond lengths are in Å.

According to the previous experimental studies (Hormann et al., 1986), AuCN can readily react with a molecular phosphine ligand to generate a linear PEt_3_AuCN species **1**. It is generally accepted that linear Au(I) could not associate with a second or third ligand because this is an entropy decreasing process (Schwerdtfeger et al., 2003; Carvajal et al., 2004). However, our calculations show that the association of PEt_3_AuCN with two additional PEt_3_ ligands to form a tetracoordinated Au(I) species **3** is exergonic by 9.9 kcal/mol (relative to complex **1**, see Scheme 2. This result indicates the formation of the tetracoordinated Au (I) complex **3** is possible. In fact, in the related experimental studies (Skouta and Li, 2007), 25-fold amounts of phosphine ligand (with respect to AuCN) is required to achieve a good yield of the isoflavanone derivatives. These theoretical and experimental results suggest that the tetracoordinated Au (I) complex **3** could be formed under the experimental conditions. Although the reaction of Au (I) complex **3** with another PEt_3_ molecule could generate a zwitterionic intermediate (PEt_3_)_4_PAu^+^…CN^−^, this process is thermodynamically unfavorable (highly endergonic by 15.1 kcal/mol). Thus, we assume that the tetracoordinated complex **3** might be involved in the catalytic cycle. The elongation of Au-CN bond length in (PEt_3_)_3_PAuCN by 0.14 Å (compared to PEt_3_AuCN) indicates the Au-CN bond strength in (PEt_3_)_3_PAuCN is weakened, increasing the nucleophilic reactivity of cyanide ion.

#### HCN Promoted Formation of α-Cyano Carbanion Intermediate

**Figure 2:**
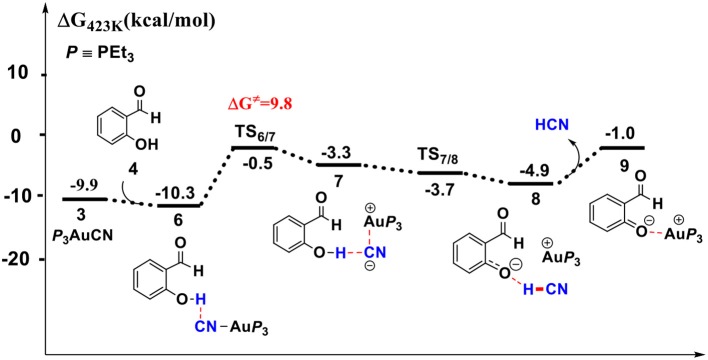
Gibbs free energy profile for the formation of free HCN molecule in toluene solution. Relative free energies (with respect to separated reactants) are given in kcal/mol.

**Figure 3:**
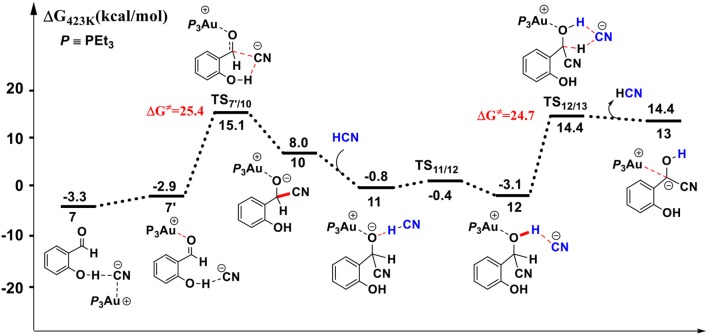
Gibbs free energy profile for the formation of the α-cyano carbanion intermediate **13** in toluene solution. Relative free energies (with respect to separated reactants) are given in kcal/mol.

**Figure 4:**
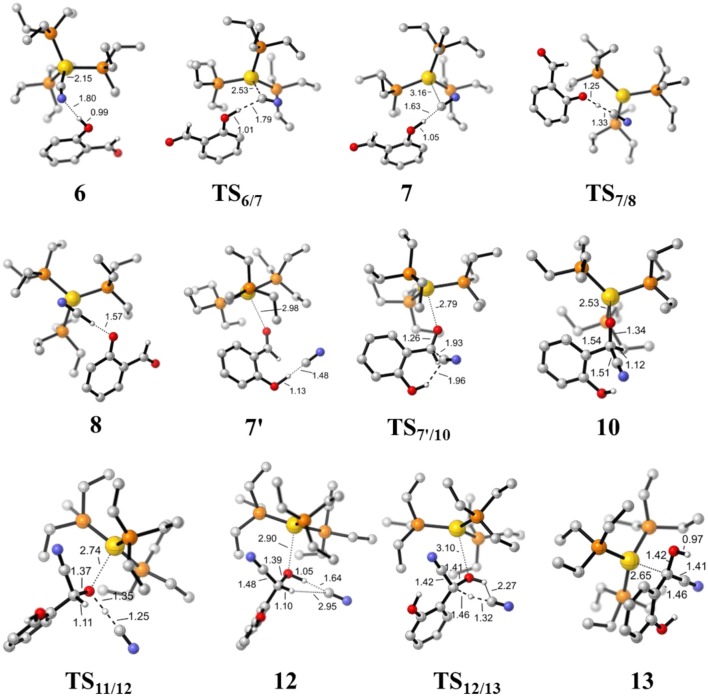
Optimized geometries of some species involved in the formation of the HCN and α-cyano carbanion intermediate **13**. Hydrogen atoms except for those involved in the reaction are omitted for clarity. All bond lengths are in Å.

With (PEt_3_)_3_AuCN **3** as the active catalyst, we found that the cyano group of catalyst **3** could react with the phenolic hydroxyl group of SA to liberate the HCN molecule, which then occurs nucleophilic addition toward the carbonyl of SA to generate a α-cyano carbanion intermediate **13**. The Gibbs free energy profiles for these processes are presented in Figures 2, 3, respectively. The optimized geometries of the involved species along these reaction pathways are displayed in Figure 4.

First, complexation of the catalyst **3** and SA forms the complex **6**, which is stabilized by a O-H…N hydrogen bond interaction (*d*_O−H…N_ = 1.80 Å) and exergonic by 0.4 kcal/mol, as shown in Figure 2. Then, the dissociation of cyanide ion from the Au(I) center in complex **6** leads to an [Au(I)]^+^…*CN*^−^ ion-pair complex **7** (via the transition state **TS**_6/7_). Both the transition state **TS**_6/7_ and ion-pair complex **7** could be stabilized by strong hydrogen bond interaction with the phenolic hydroxyl of SA, in which the distances of O-H…CN^−^ are 1.79 and 1.63 Å, respectively. This process is slightly endergonic by 7.0 kcal/mol with a barrier of 9.3 kcal/mol. Subsequently, complex **7** undergoes a proton transfer process from the acidic phenolic hydroxyl to the cyanide ion to generate a new ternary ion-pair complex **8**, HCN-SA^−^-(PEt_3_)_3_Au^+^. The liberation of HCN from the complex **8** forms the complex **9**, SA^–^ – (*PEt*_*3*_)_3_Au^+^. Overall, the formation of the separated HCN and complex **9** is endergonic by 9.3 kcal/mol with a free energy barrier of only 9.8 kcal/mol (with respect to complex **6**). These results indicate that a free HCN molecule may be generated under the experimental conditions (150°C).

For the species **7**, it may convert into a new complex **7′**, in which the cationic gold center of (PEt_3_)_3_Au^+^ is associated with the oxygen atom of carbonyl group (*d*_Au−O_ = 2.86 Å) (see Figure 3). Cyanide ion in complex **7′** subsequently attacks the carbonyl carbon atom of SA (with the assistance of the phenolic hydroxy), leading to a gold-ligated alkoxide intermediate **10** via the transition state **TS**_7′/10_ (with a barrier of 25.4 kcal/mol). Then, the association of the basic alkoxide intermediate **10** and a free HCN (generated through the pathway described above) forms a hydrogen bond stabilized complex **11** (*d*_O…H−CN_ = 1.39 Å). The following intramolecular proton transfer leads to an Au^+^-cyanohydrin-CN^−^ complex **12** (via transition state **TS**_11/12_). The cyanide ion in complex **12**, stabilized by hydrogen bond interaction with cyanohydrin, could abstract the α-H of cyanohydrin to form α-cyano carbanion intermediate **13** and regenerate the HCN simultaneously. The free energy barrier for the CN^−^ assisted proton abstraction (via transition state **T2**_12/13_) is 24.7 kcal/mol. In addition, the pathway involving SA-catalyzed intramolecular 1,2 H-shift of the gold-ligated alkoxide intermediate **10** could also form α-cyano carbanion intermediate **13** (via transition state **TS**_12/13−SA_, see Figure S1 for details). In summary, the HCN promoted carbonyl umpolung process is endergonic by 24.7 kcal/mol (relative to intermediate **6**). The rate-limiting step for this stage is the nucleophilic attack of cyanide ion at the carbonyl carbon atom with an activation barrier of 25.4 kcal/mol.

Other reaction channels to form the alkoxide intermediate **10**, starting from complex **6** or **9**, could also be located with the MD/CD method (see Figure S2 for details). However, the related free energy barriers for these pathways are higher by 3~5 kcal/mol than the pathway listed in Figure 3. Therefore, the pathway, starting from complexes **7** or **7′** is responsible for the formation of alkoxide intermediate **10**, which then converts into α-cyano carbanion intermediate **13**.

#### Hydroacylation of Phenylacetylene

**Figure 5:**
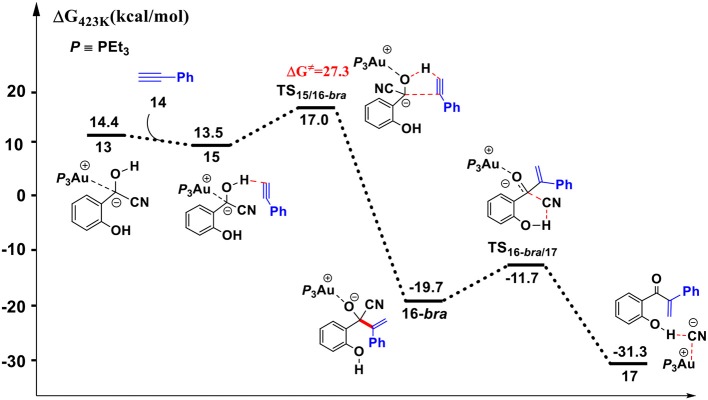
Gibbs free energy profile for the hydroacylation of phenylacetylene process. Relative free energies (with respect to separated reactants) are given in kcal/mol.

**Figure 6:**
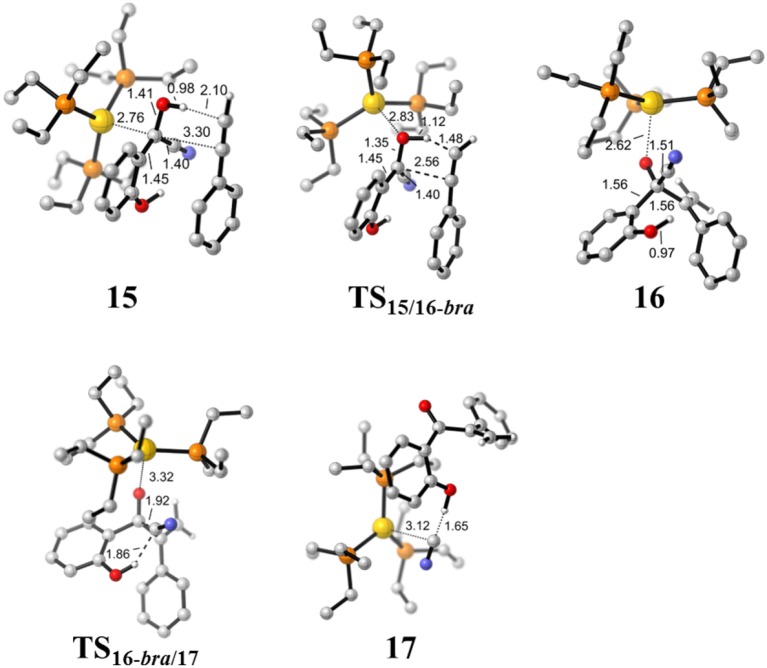
Optimized geometries of some species involved in the hydroacylation of phenylacetylene. All bond lengths are in Å.

Addition of cyanide ion to SA leads to an umpolung of the carbonyl SA, and the corresponding α-cyano carbanion intermediate **13** could act as an acyl anion equivalent to react with phenylacetylene **14**, forming a branched α-β unsaturated ketone complex **17** and regenerating the cyanide ion. The Gibbs free energy profile for this process is presented in Figure 5. The optimized geometries of some stationary points are displayed in Figure 6.

First, complexation of the phenyl acetylene **14** with the α-cyano carbanion intermediate **13** forms an intermolecular complex **15**. Then, a branched hydroacylation intermediate **16*-bra*** could be generated via a concerted alkyne-Ene transition state (Piel et al., 2011; Schedler et al., 2013) (transition state **TS**_15/16**−*bra***_), in which, the proton transfer from the α-cyano carbanion intermediate to the terminal carbon (C_1_) of phenylacetylene is accomplished with an nucleophilic attack of the acetyl anion center at the C_2_ position of phenyl acetylene. The formation of intermediate **16*-bra*** is exergonic by 33.2 kcal/mol with a barrier of 27.3 kcal/mol. Subsequently, the elimination of the cyanide ion from intermediate **16**, with the assistance of the phenolic hydroxyl (via transition state **TS**_16-***bra***/17_**)**, forms a branched α-β unsaturated ketone complex **17** (Other possible reaction pathways starting from intermediate **16-*bra*** are presented in Figure S3). This ternary intermediate **17** was stabilized by multiple non-covalent interactions, including the electrostatic interaction between the Au cation and cyanide ion (*d*_Au…CN_ = 3.12 Å) and a strong hydrogen bond interaction (*d*_O−H…CN−_ = 1.65 Å) formed between the cyanide ion and phenolic hydroxyl group. Along the energy profile described above, we can find that this hydroacylation process is exergonic by 45.7 kcal/mol, relative to the intermediates **13** and **14**. The concerted alkyne-Ene process is the rate-determining step with a free energy barrier of 27.3 kcal/mol.

#### Intramolecular Oxa-Michael Addition

**Figure 7:**
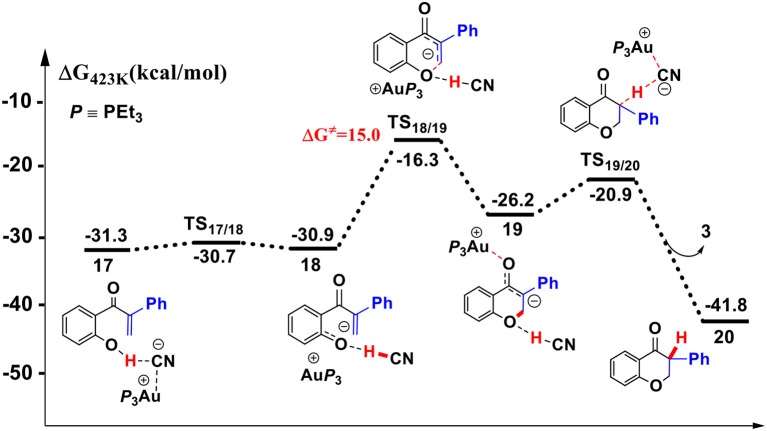
Gibbs free energy profile of the intramolecular oxa-Michael process in toluene solution. Relative free energies (with respect to separated reactants) are given in kcal/mol.

**Figure 8:**
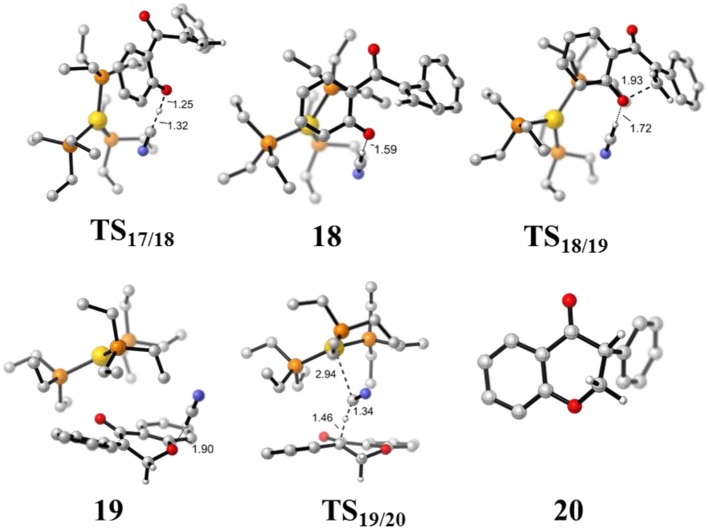
Optimized geometries of some species involved in the intramolecular oxa-Michael process. All bond lengths are in Å.

With the assistance of a basic cyanide ion, the branched α,β-unsaturated ketone proceeds through an intramolecular oxa-Michael addition to form the isoflavanone **20** and regenerate the active catalyst **3**. The Gibbs free energy profile and the optimized geometries of involved stationary points along the reaction pathway are displayed in Figures 7, 8, respectively.

First, the deprotonation of the phenolic hydroxyl by the cyanide ion forms a complex **18** (via transition state **TS**_17/18_) containing phenol anion, HCN, and (PEt_3_)_3_Au cation. The formation of the complex **18** is slightly endergonic, in which the nucleophilicity of the oxygen atom of phenol is increased. Then, the complex **18** could convert into the complex **19** via an intramolecular oxa-Michael addition reaction (via transition state **TS**_18/19_). Finally, protonation of the enolate anion center with HCN forms the isoflavanone product **20** and regenerates the active catalyst (PEt_3_)_3_AuCN **3**. The process described above is exergonic by 10.5 kcal/mol (relative to intermediate **17**), and the rate-limiting step is the intramolecular nucleophilic addition reaction of complex **18** with a free energy barrier of 15.0 kcal/mol.

**Scheme 3:**
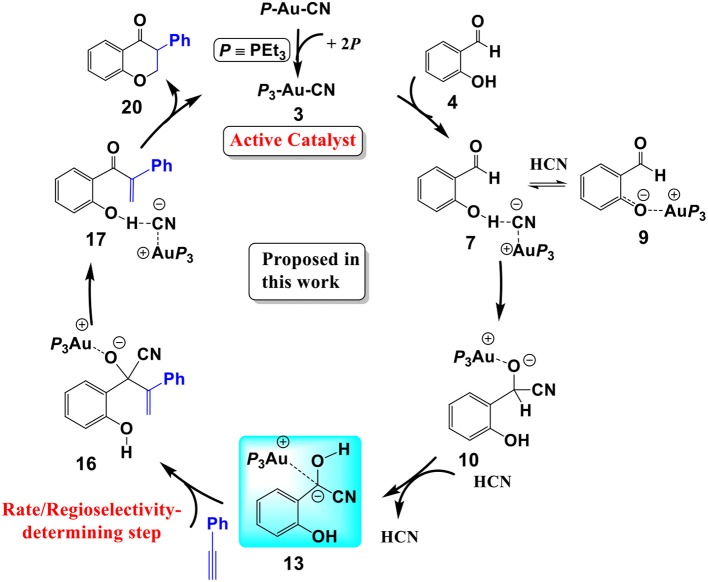
The cyanide ion promoted umpolung hydroacylation/intramolecular oxa-Michael addition mechanism proposed in this work.

In summary, our calculations reveal that a cyanide ion promoted umpolung hydroacylation/intramolecular oxa-Michael addition mechanism, as shown in Scheme 3, is more favorable than the Au(I)/Au(III) redox mechanism proposed previously. The new pathway contains four stages described above. The overall reaction is exergonic by 41.8 kcal/mol. The hydroacylation of phenyl acetylene is the rate-determining step with a free energy barrier of 27.3 kcal/mol. The free energy barrier is in accord with the experimental fact that the studied reaction takes place at 150°C.

### Possible Theoretical Explanations of Experimental Findings

**Scheme 4:**
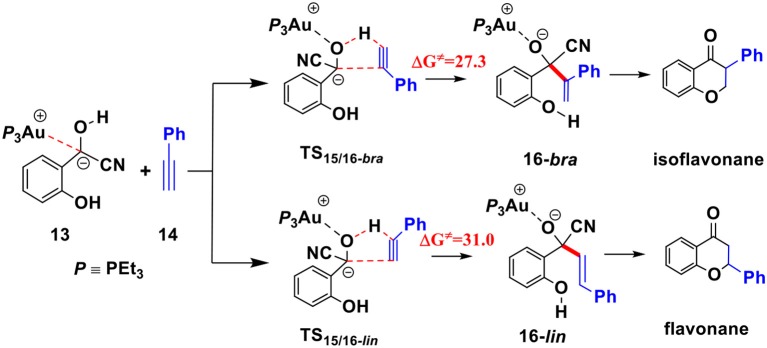
Two possible pathways for the hydroacylation of phenylacetylene; Activation free energy barriers (ΔG^≠^) are given in kcal/mol.

To understand the experimental fact that only the isoflavanone product is observed, we also explored the pathway involving the formation of linear enone intermediate **16*-lin*** (related to the formation of flavanone product) via **TS**_15/16-***lin***_ (see Scheme 4). The free energy barrier of this transition state is 31.0 kcal/mol, which is higher than **TS**_15/16-***bra***_ by 3.7 kcal/mol. These results indicate that the formation of isoflavanone product is dynamically favorable, which is in accord with the experimental observed regioselectivity.

In addition to PEt_3_, we also investigated the impact of the ligand on the reactivity of this annulation reaction, including PBu_3_, and PPh_3_. Key transition states with relative higher free energies (**TS**_7′/10_, **TS**_12/13_, **TS**_15/16-***bra***_), involved in the pathway, were determined to evaluate the ligand effect. The free energy barriers of these different TSs with different phosphines are listed in Table 1 and the related optimized geometries are presented in Figure S4. The calculated results show that the rate-determining step for the studied reaction is the hydroacylation of phenyl acetylene (**TS**_15/16-***bra***_) with free energy barriers of 27.3 and 32.0 kcal/mol, respectively, when the ligands are PEt_3_, and PPh_3_, respectively. However, for the PBu_3_ ligand, both the HCN-assisted 1,2 H-shift (**TS**_12/13_) and hydroacylation of phenyl acetylene (**TS**_15/16-***bra***_) may be the rate-determining steps, with comparable barriers (25.0 and 24.9 kcal/mol, respectively). The calculated energy barriers are in qualitative accord with the experimental fact that ligands PEt_3_ and PBu_3_ provided moderate to good yield (36 and 78%, respectively), while for the ligand PPh_3_, only trace amounts of products were observed.

**Table 1:** Comparison of the free energy barriers (kcal/mol) of different TSs with various ligands (PEt_3_, PBu_3_, and PPh_3_).

**Ligands**	** 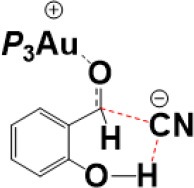 **	** 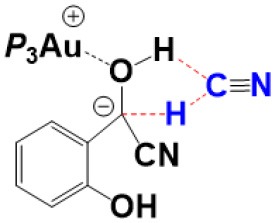 **	** 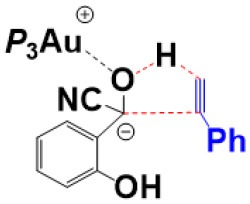 **	**Exp. Yield [%]**
PEt_3_	25.4	24.7	27.3	36
PBu_3_	22.1	25.0	24.9	78
PPh_3_	29.3	28.0	31.2	trace

**Scheme 5:**
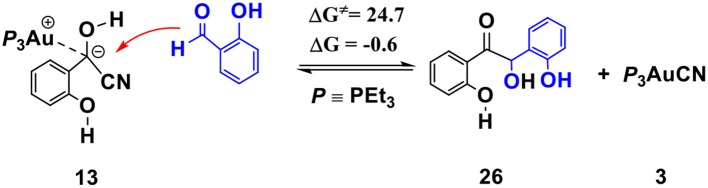
Activation free energy barrier (ΔG^≠^) and reaction energy (ΔG) of the competitive benzoin condensation pathway of SA via α-cyano carbanion intermediate **13** (all energies are given in kcal/mol).

The competing benzoin condensation (Wöhler, 1832; Zinin, 1839, 1840) pathway of SA via α-cyano carbanion intermediate **13** (see Scheme 5) is also possible. The free energy barrier of the rate-limiting step in this competing pathway is 24.7 kcal/mol (see Figures S5, S6 for more details). However, this process is almost thermally neutral (slightly exergonic by 0.6 kcal/mol, relative to separated reactants), which is incomparable with the pathway of the annulation of SA and phenyl acetylene (described above, exergonic by 41.8 kcal/mol). This result indicates that the generation of the isoflavanone-type product is thermodynamically much more favorable. Our calculations are in accord with the experimental finding that excessive amount of phenyl acetylene (3-fold amounts of SA) are required to improve the yield of the isoflavanone product.

## Conclusions

We have performed DFT calculations assisted by the MD/CD method to explore the detailed mechanism of the AuCN-catalyzed annulation of salicylaldehyde (SA) and phenyl acetylene. Our calculations reveal that a cyanide ion promoted umpolung hydroacylation/intramolecular oxa-Michael addition mechanism is more favorable than the Au(I)/Au(III) redox mechanism proposed previously. The new proposed pathway contains four stages, as shown in Scheme 3: (1) the association of AuCN with three PEt_3_ molecule generates an active catalyst (PEt_3_)_3_AuCN **3**; (2) The nucleophilic attack of the cyanide ion on the carbonyl in SA and subsequent HCN-assisted 1,2 H-shift provide the α-cyano carbanion intermediate **13**, which is a key intermediate involved in this mechanism; (3) the hydroacylation of phenylacetylene, followed by the elimination of the cyanide ion, leads to a branched α-β unsaturated ketone **17**; (4) the intramolecular conjugate addition of the α,β unsaturated ketone forms the isoflavanone product **20** and regenerates the active catalyst **3**. The overall reaction is exergonic by 41.8 kcal/mol. The hydroacylation of phenyl acetylene is the rate-determining step and responsible for the regioselectivity with a free energy barrier of 27.3 kcal/mol at T = 423 K and 1.0 atm in toluene. In the umpolung mechanism, the hydroxyl of SA is found to strongly stabilize the cyanide ion involved intermediates and transition states via hydrogen bond interactions, while the Au(I) ion always acts as a counter cation. Our results are in qualitative accord with the experimental findings. The results provide important insight into Au(I)-catalyzed annulation of SA and aryl acetylenes, which may be useful in designing more effective catalysts for synthesis of heterocyclic frameworks.

## Data Availability

All datasets generated for this study are included in the manuscript/Supplementary Files.

## Author Contributions

The work was completed by cooperation of all authors. MY, GW, JZ, and SL were responsible for the study of concept and design of the project. MY performed corresponding calculations. MY, GW, JZ, and SL drafted and revised the manuscript.

### Conflict of Interest Statement

The authors declare that the research was conducted in the absence of any commercial or financial relationships that could be construed as a potential conflict of interest.
