# Supplementary material for: Mechanistic Insight Into the AuCN Catalyzed Annulation Reaction of Salicylaldehyde and Aryl Acetylene: Cyanide Ion Promoted Umpolung Hydroacylation/Intramolecular Oxa-Michael Addition Mechanism
Source: Front Chem. 2019 Aug 6;7:557. doi: 10.3389/fchem.2019.00557 (PMC6691126; doi:10.3389/fchem.2019.00557)
Supplement: Supplementary file 1 [file Data_Sheet_1.docx]

Supplementary Materials for

**Mechanistic Insight into the AuCN Catalyzed Annulation Reaction of** **Salicylaldehyde and Aryl Acetylene: Cyanide Ion Promoted Umpolung Hydroacylation/Intramolecular Oxa-Michael Addition Mechanism**

**Manyi Yang, ^‡^ Guoqiang Wang,^‡^ Jingxiang Zou, Shuhua Li^*^**

School of Chemistry and Chemical Engineering, Key Laboratory of Mesoscopic Chemistry of Ministry of Education, Institute of Theoretical and Computational Chemistry, Nanjing University, Nanjing, 210023, People’s Republic of China

^‡^ These authors contributed equally to this work

*** Correspondence:**Corresponding Author
shuhua@nju.edu.cn

**Figure S1**. Gibbs free energy profile of another possible pathways leading to the cyanohydrin intermediate **13** in toluene solution. Relative free energies calculated at 423 K and 1 atm (with respect to separated reactants) are given in kcal/mol. Optimization geometries of the transition states are presented; All listed distances are in Å.

**Figure S2**. Gibbs free energy profile of other possible reaction pathways to generate the alkoxide intermediate **10**. Relative free energies (with respect to separated reactants) are given in kcal/mol. Optimization geometries of the transition states are presented; All listed distances are in Å.

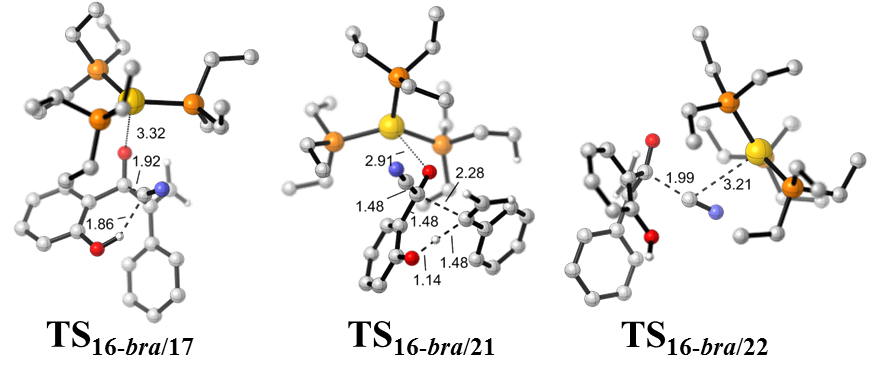


**Figure S3**. Gibbs free energy profile of possible reaction pathways starting from intermediate **16*-bra***. Relative free energies (with respect to separated reactants) are given in kcal/mol. Optimization geometries of the transition states are presented; All listed distances are in Å.


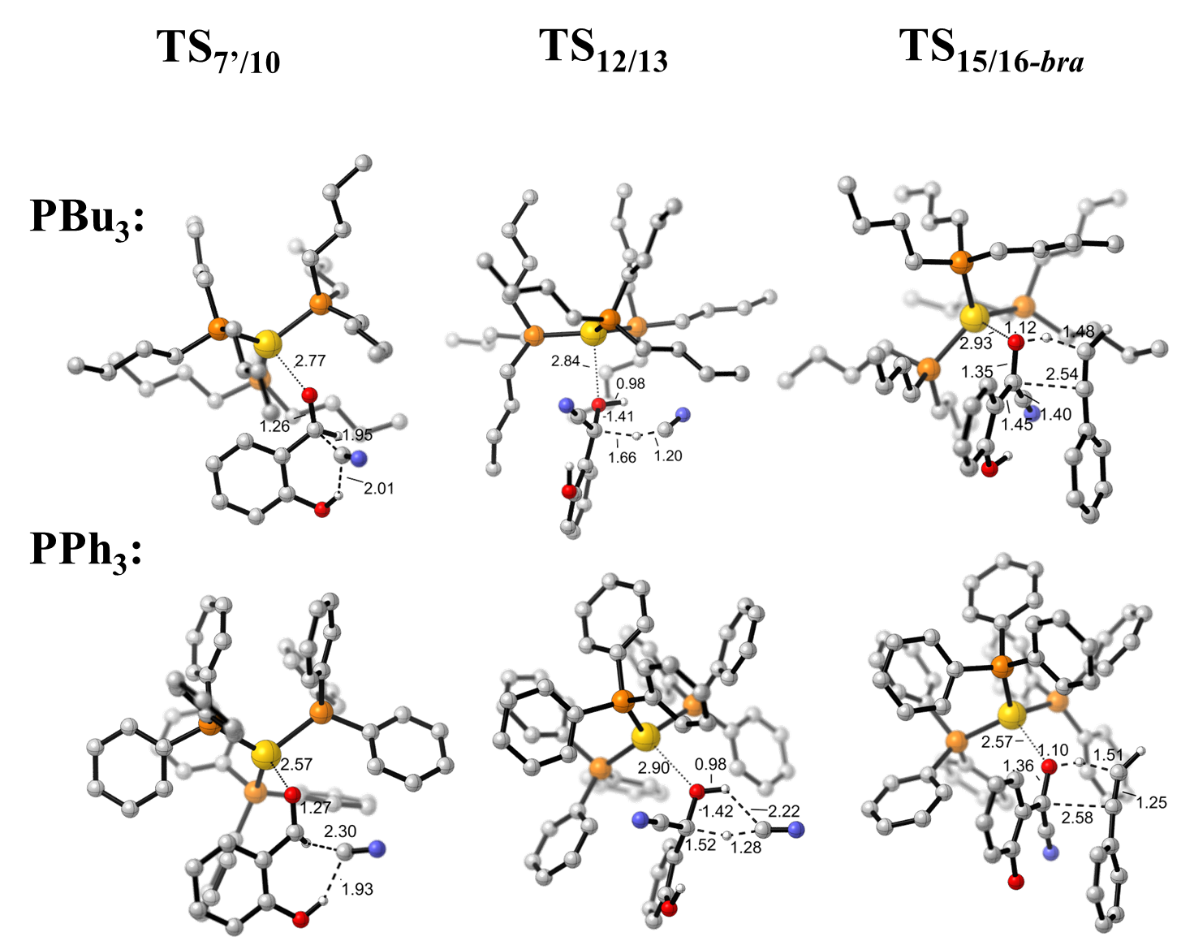


**Figure S4**. The optimization geometries of different transition states for various phosphine ligands (PBu_3_, and PPh_3_). All bond lengths are in Å.

**Figure S5**. Gibbs free energy profiles of the condensation process leading to benzoin-type product **25**. Relative free energies (with respect to separated reactants) are given in kcal/mol.


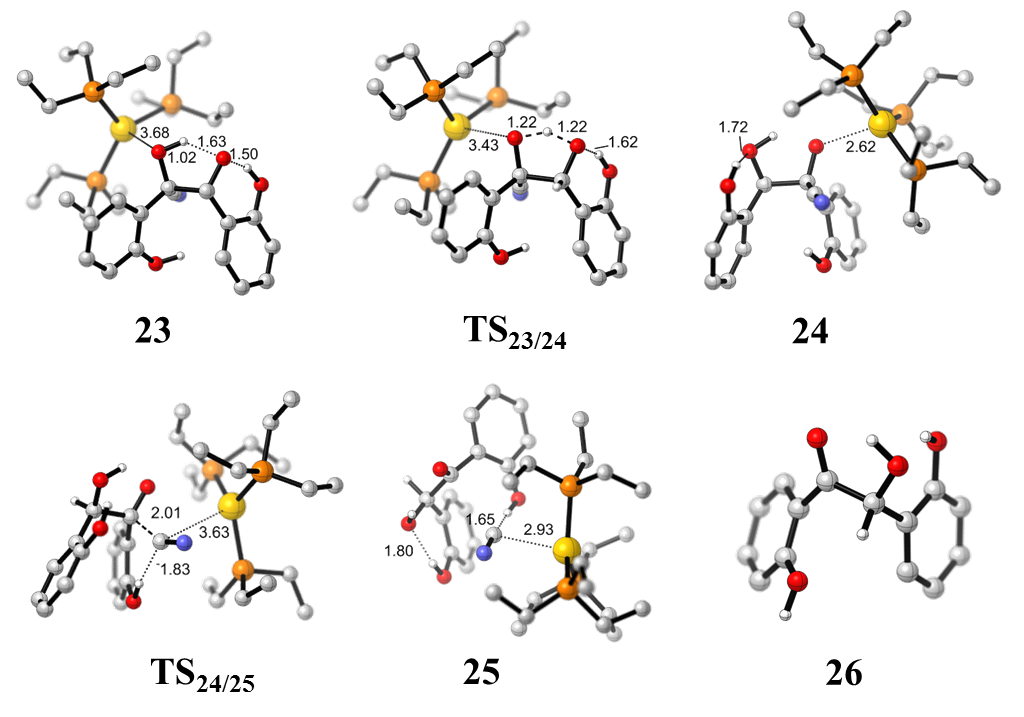


**Figure S6.** Optimized structures of some species involved in the condensation process leading to benzoin-type product **25**. Hydrogen atoms except for those involved in the reaction are omitted for clarity. All bond lengths are in Å.

## XYZ coordinates for all reported structures investigated computationally

Structure: AuCN

Symbol X Y Z

Au 1.820105 7.960068 5.023826

C 1.820752 6.812287 3.478041

N 1.815143 6.163645 2.507131

Structure: HCN

Symbol X Y Z

C 1.016111 1.013590 -0.000000

N 2.173264 1.013590 0.000000

H -0.057605 1.013590 0.000000

Structure: PEt_3_

Symbol X Y Z

P 1.673369 9.180480 6.776605

C 3.335382 9.751847 7.388046

C 3.391229 10.543538 8.691414

C 0.688007 10.753687 6.831085

C 1.171683 11.792967 5.821223

C 0.979640 8.349116 8.285657

C 1.747658 7.087205 8.675027

H 3.773019 10.335341 6.567516

H 3.954437 8.848313 7.463170

H 4.425945 10.814846 8.935421

H 2.996828 9.965705 9.534444

H 2.815768 11.473472 8.626544

H 0.688482 11.171550 7.846448

H -0.348748 10.468976 6.608353

H 0.512970 12.669580 5.812602

H 1.194413 11.379886 4.805661

H 2.182459 12.143664 6.059451

H -0.059560 8.094272 8.039462

H 0.939946 9.052735 9.127691

H 1.257258 6.569456 9.508014

H 2.770838 7.321699 8.990510

H 1.811976 6.385664 7.834657

Structure: 1

Symbol X Y Z

Au 1.727532 7.992638 4.997766

P 1.685142 9.233076 6.943848

C 1.761284 6.910206 3.304521

N 1.780736 6.279581 2.319915

C 3.360650 9.773253 7.452400

C 3.427923 10.601211 8.732983

C 0.670497 10.751095 6.819378

C 1.170219 11.711817 5.742179

C 0.981270 8.326565 8.369292

C 1.761080 7.058015 8.709615

H 3.774201 10.325821 6.600873

H 3.962198 8.860953 7.537791

H 4.467303 10.871184 8.949072

H 3.045468 10.047517 9.596717

H 2.857507 11.531837 8.646606

H 0.640517 11.234894 7.803078

H -0.348469 10.414979 6.593885

H 0.491791 12.566678 5.652217

H 1.223818 11.217090 4.766208

H 2.165701 12.102216 5.978730

H -0.050608 8.082076 8.090848

H 0.931397 9.006849 9.227951

H 1.263329 6.512319 9.518079

H 2.779484 7.286050 9.041631

H 1.829034 6.392081 7.842419

Structure: 2

Symbol X Y Z

Au 0.953912 4.146635 1.006297

P -0.995625 4.170609 -0.365553

C -0.934055 2.955582 -1.746193

C -2.128936 2.916227 -2.694247

C -2.548706 3.786958 0.536046

C -2.467738 2.471365 1.307419

C -1.378592 5.775321 -1.173366

C -0.229631 6.264415 -2.051798

H -0.772228 1.980853 -1.270759

H -0.006702 3.170135 -2.290984

H -1.977670 2.148907 -3.462339

H -2.271857 3.872213 -3.209563

H -3.058600 2.674005 -2.168171

H -3.388130 3.783116 -0.170207

H -2.714775 4.625384 1.223133

H -3.385955 2.302684 1.881048

H -1.623308 2.476528 2.005521

H -2.334497 1.617606 0.634390

H -1.565086 6.482425 -0.355768

H -2.309992 5.693955 -1.747623

H -0.428170 7.273681 -2.429175

H -0.079324 5.609537 -2.916978

H 0.709192 6.290079 -1.486747

P 1.467670 6.094430 2.260592

C 1.202697 7.672154 1.347826

C 1.565241 8.962972 2.076260

H 1.344491 9.830766 1.443813

H 0.997098 9.080593 3.005146

H 2.630747 9.001210 2.325573

H 1.771668 7.580668 0.415093

H 0.147034 7.671369 1.052069

C 3.189516 6.209626 2.887998

C 4.214473 6.184283 1.756321

H 5.233305 6.162810 2.158339

H 4.078455 5.294472 1.131287

H 4.129836 7.068234 1.113872

H 3.302667 7.105447 3.511698

H 3.324797 5.337874 3.539405

C 0.460258 6.259315 3.788043

C -1.039598 6.171207 3.518396

H -1.607343 6.259872 4.451406

H -1.378620 6.967445 2.845987

H -1.291087 5.211365 3.055505

H 0.776267 5.441464 4.446779

H 0.719879 7.197131 4.294312

C 2.143737 2.445197 1.064794

N 2.816185 1.484720 1.090969

Structure: 3

Symbol X Y Z

Au 0.360295 3.488758 3.568354

P -2.025479 3.289130 3.525604

C -2.774820 3.528293 1.857390

C -4.295705 3.463797 1.759146

C -2.733827 1.682139 4.076125

C -2.236501 0.512926 3.229139

C -2.924289 4.507294 4.572331

C -2.661687 5.943902 4.126890

H -2.313336 2.769885 1.214729

H -2.403176 4.494896 1.497160

H -4.619191 3.625111 0.723891

H -4.776017 4.230269 2.376881

H -4.680338 2.488273 2.074657

H -3.829952 1.740475 4.075743

H -2.421885 1.550094 5.118594

H -2.594824 -0.438896 3.637411

H -1.142091 0.486024 3.210367

H -2.594113 0.584595 2.195580

H -2.560226 4.348743 5.594490

H -3.998810 4.284359 4.577081

H -3.062639 6.661146 4.852055

H -3.130140 6.153683 3.158804

H -1.586177 6.126018 4.022425

P 1.160882 4.438535 5.641206

C 0.368094 5.950713 6.338348

C 0.863158 6.422586 7.702530

H 0.335551 7.333947 8.008660

H 0.692002 5.667002 8.476578

H 1.934355 6.650757 7.689400

H 0.494163 6.737400 5.584453

H -0.707388 5.741954 6.368555

C 2.963375 4.809787 5.712303

C 3.355251 6.049164 4.916317

H 4.444868 6.155403 4.868227

H 2.975258 5.988821 3.892934

H 2.950961 6.963500 5.364881

H 3.277208 4.902938 6.759896

H 3.456718 3.919026 5.304908

C 1.018323 3.199658 6.993427

C -0.424297 2.821734 7.308259

H -0.463620 2.023191 8.057560

H -0.993367 3.673346 7.699400

H -0.926597 2.461834 6.405319

H 1.567036 2.320869 6.634028

H 1.535149 3.568718 7.888209

C 1.348308 1.584788 3.744383

N 1.927960 0.573214 3.880904

P 1.074211 4.429561 1.479181

C 2.901481 4.407555 1.241266

C 3.447611 4.947198 -0.076938

C 0.443736 3.530144 0.000513

C 0.705443 2.028137 0.062293

C 0.559730 6.151920 1.078620

C 1.066607 7.182190 2.081381

H 3.195542 3.361891 1.393747

H 3.327173 4.955155 2.088899

H 4.542472 4.888255 -0.087115

H 3.173824 5.996900 -0.231118

H 3.082008 4.373967 -0.935304

H 0.875393 3.973383 -0.905879

H -0.633527 3.727492 -0.036625

H 0.253486 1.520808 -0.797983

H 0.291839 1.598452 0.980072

H 1.778026 1.805108 0.055223

H -0.537480 6.134443 1.081269

H 0.873516 6.406137 0.057947

H 0.639681 8.170653 1.876749

H 2.157304 7.277863 2.042727

H 0.791281 6.895393 3.102267

Structure: 4

Symbol X Y Z

C -5.019306 -2.026310 0.298803

C -3.635157 -2.038211 0.439916

C -2.923325 -0.834421 0.427473

C -3.607832 0.387105 0.272265

C -5.004442 0.371300 0.131825

C -5.714071 -0.820616 0.143674

H -5.560416 -2.968815 0.310192

H -3.103654 -2.979904 0.559786

H -5.510065 1.325468 0.013451

H -6.794317 -0.820456 0.034207

C -2.880883 1.665953 0.256051

O -3.421351 2.755256 0.125257

H -1.784912 1.601681 0.371122

O -1.572578 -0.797238 0.561808

H -1.225207 -1.696595 0.660022

Structure: AuCN_4PEt_3_

Symbol X Y Z

Au -3.930342 5.511535 3.955024

P -6.227258 4.699733 3.925303

C -6.744284 3.670352 2.483360

C -8.215021 3.262093 2.444370

C -6.690757 3.645004 5.353103

C -6.162265 2.219383 5.200040

C -7.492869 6.035250 3.890205

C -7.462239 6.816024 2.578321

H -6.106005 2.781134 2.504966

H -6.477785 4.224948 1.576586

H -8.399448 2.590643 1.597419

H -8.875934 4.126872 2.327552

H -8.514197 2.734531 3.355548

H -7.780061 3.644006 5.480835

H -6.269392 4.098627 6.255936

H -6.153287 1.732080 6.178997

H -5.135338 2.214118 4.818211

H -6.776881 1.630124 4.511172

H -7.279723 6.694985 4.734175

H -8.484592 5.601699 4.067227

H -8.068434 7.726351 2.648074

H -7.855253 6.221149 1.746875

H -6.438701 7.109126 2.320437

P -3.814092 7.913205 4.500823

C -4.610812 9.069680 3.300126

C -4.259433 10.550465 3.408292

H -4.844756 11.129300 2.684195

H -4.478728 10.954306 4.402208

H -3.201130 10.733490 3.195418

H -4.385608 8.693590 2.298403

H -5.688702 8.923455 3.431684

C -2.077113 8.519216 4.623999

C -1.241535 8.233314 3.385091

H -0.210943 8.581478 3.517801

H -1.218337 7.158320 3.188781

H -1.648901 8.728766 2.496409

H -2.087870 9.590171 4.860541

H -1.641878 8.010594 5.492704

C -4.446960 8.558076 6.112527

C -5.938199 8.368785 6.355029

H -6.220905 8.790604 7.325724

H -6.545642 8.865118 5.590261

H -6.203466 7.308307 6.370632

H -3.876568 8.054110 6.897823

H -4.181035 9.621154 6.168864

C -6.397234 2.980939 8.797998

N -5.445174 2.339023 8.519903

P -3.110216 4.981810 1.703423

C -1.328539 5.199812 1.258638

C -0.863582 4.567432 -0.050299

C -3.474162 3.240373 1.220854

C -3.008261 2.190888 2.220848

C -3.969833 5.863082 0.320780

C -3.403305 7.245660 0.012204

H -0.734362 4.827130 2.098048

H -1.162589 6.281781 1.235696

H 0.164765 4.874910 -0.273922

H -1.489540 4.869190 -0.897691

H -0.874064 3.474144 0.002362

H -3.051523 3.057029 0.225949

H -4.560416 3.194983 1.105385

H -3.393019 1.200782 1.951778

H -3.362641 2.433716 3.227916

H -1.916088 2.122259 2.255563

H -5.019131 5.940829 0.626802

H -3.944303 5.233252 -0.577688

H -4.084051 7.808817 -0.635590

H -2.438239 7.178705 -0.500204

H -3.249600 7.829970 0.924398

P -2.555392 4.590525 5.759564

C -2.875502 2.886056 6.363292

C -1.994413 2.399792 7.510200

C -0.737992 4.622687 5.437952

C -0.271068 3.503610 4.514097

C -2.651768 5.585966 7.301343

C -4.052008 5.587757 7.901758

H -2.791330 2.223047 5.494067

H -3.921871 2.852840 6.688744

H -2.245054 1.360111 7.746372

H -2.173877 2.980561 8.419553

H -0.925110 2.445610 7.274482

H -0.206360 4.575280 6.396659

H -0.519933 5.602934 4.997859

H 0.771474 3.652218 4.210510

H -0.884583 3.458289 3.611007

H -0.341492 2.526607 5.002442

H -2.320084 6.598406 7.042085

H -1.919154 5.194213 8.017390

H -4.122741 6.297950 8.733932

H -4.337519 4.600544 8.276151

H -4.796732 5.869665 7.150749

Structure: 5

Symbol X Y Z

C -3.957973 1.238962 1.869485

C -3.242602 1.297963 3.058549

C -3.108176 2.519475 3.735523

C -3.688193 3.684398 3.191519

C -4.421418 3.593894 1.996317

C -4.562388 2.385151 1.333627

H -4.052479 0.285384 1.356477

H -2.778188 0.406686 3.469132

H -4.870654 4.503999 1.607474

H -5.135657 2.323136 0.413040

C -3.495775 4.988462 3.836857

O -3.907803 6.044914 3.372187

O -2.440832 2.620139 4.904567

H -1.790639 1.892490 4.983568

Au -0.284142 2.683502 1.960954

P -0.730228 4.131322 0.222189

C 0.795385 4.582451 -0.688353

C 0.605605 5.526811 -1.872468

C -1.892665 3.446225 -1.009435

C -1.414332 2.126736 -1.609952

C -1.497243 5.696772 0.767347

C -0.673158 6.426394 1.825023

H 1.253363 3.638300 -1.005230

H 1.476677 5.012397 0.055036

H 1.573117 5.735728 -2.341837

H 0.175725 6.485347 -1.564383

H -0.044930 5.093027 -2.638917

H -2.069136 4.201778 -1.784293

H -2.836281 3.302937 -0.471910

H -2.169622 1.724971 -2.293766

H -1.236169 1.382621 -0.825757

H -0.485178 2.249759 -2.176835

H -2.480724 5.431586 1.168753

H -1.669150 6.326855 -0.113437

H -1.207453 7.317939 2.167367

H 0.300400 6.744088 1.436232

H -0.496990 5.784836 2.695442

C 0.026256 1.458758 3.530459

N 0.031237 0.802083 4.499783

H -2.928672 4.981479 4.782642

Structure: TS_5/Ia_

Symbol X Y Z

C -5.448118 1.437283 1.405542

C -4.261899 0.924012 1.920065

C -3.364086 1.759350 2.591216

C -3.662873 3.126643 2.756934

C -4.874322 3.615438 2.241430

C -5.762588 2.790148 1.565229

H -6.129030 0.774948 0.877737

H -4.022983 -0.130547 1.799416

H -5.088014 4.671159 2.379280

H -6.688516 3.191679 1.164566

C -2.738556 4.119521 3.361087

O -3.109351 5.235949 3.688810

O -2.197615 1.283234 3.093612

H -2.123298 0.330896 2.927698

Au -0.675041 3.787586 2.994299

P -0.715578 4.462621 0.717657

C 0.953667 4.587281 -0.013024

C 1.003924 5.018370 -1.475991

C -1.679913 3.309978 -0.323828

C -1.098035 1.897893 -0.332377

C -1.529135 6.083961 0.504841

C -0.824021 7.198582 1.275572

H 1.421583 3.607516 0.136057

H 1.513234 5.277022 0.629123

H 2.044030 5.060507 -1.817424

H 0.567941 6.012308 -1.622098

H 0.471647 4.315513 -2.125689

H -1.747647 3.721169 -1.338537

H -2.694202 3.306464 0.092873

H -1.758736 1.219737 -0.882613

H -0.985913 1.513102 0.686241

H -0.114425 1.869071 -0.812720

H -2.554643 5.955708 0.871535

H -1.592510 6.312577 -0.566085

H -1.374869 8.138849 1.168288

H 0.193874 7.364485 0.907326

H -0.766184 6.962105 2.343124

C 1.317049 3.349949 3.306143

N 2.457342 3.105116 3.397534

H -1.693277 3.512563 4.350119

Structure: Ib

Symbol X Y Z

C -5.744801 0.592277 2.470131

C -4.367650 0.402105 2.470962

C -3.506101 1.500587 2.383954

C -4.026265 2.806108 2.306790

C -5.422420 2.965981 2.290558

C -6.282034 1.880446 2.372479

H -6.401051 -0.270924 2.543500

H -3.951867 -0.601544 2.533257

H -5.806785 3.980087 2.232077

H -7.357938 2.028848 2.372195

C -3.179992 4.031256 2.297860

O -3.690057 5.122319 2.066739

O -2.158256 1.325999 2.337247

H -1.930761 0.415406 2.578987

Au -1.115309 3.944043 2.756188

P -0.653340 4.699647 0.612193

C -1.531704 3.396358 4.655267

N -1.773515 3.063643 5.747820

C 1.081958 4.428153 0.117558

C 1.442936 4.935719 -1.275916

C -1.699870 3.975995 -0.695596

C -1.512107 2.468496 -0.848419

C -0.990534 6.489489 0.558631

C -0.086044 7.305583 1.479945

H 1.263986 3.351603 0.213646

H 1.690852 4.906115 0.892590

H 2.505227 4.756511 -1.473253

H 1.265653 6.011862 -1.374870

H 0.871731 4.423833 -2.057169

H -1.479813 4.505170 -1.630871

H -2.732866 4.218328 -0.424235

H -2.215019 2.077245 -1.591315

H -1.692602 1.950762 0.098062

H -0.499871 2.220502 -1.185863

H -2.040971 6.584770 0.859073

H -0.910849 6.822447 -0.483278

H -0.383340 8.359028 1.455524

H 0.964539 7.248990 1.176138

H -0.159414 6.958417 2.515740

H 0.490949 3.968858 3.109593

Structure: Ia

Symbol X Y Z

C -5.577985 0.695414 1.981029

C -4.226739 0.487123 2.229307

C -3.372257 1.573156 2.452475

C -3.879194 2.887787 2.433894

C -5.250138 3.066131 2.176868

C -6.099112 1.993386 1.950637

H -6.225654 -0.160188 1.809116

H -3.821965 -0.522895 2.248557

H -5.624055 4.085563 2.166438

H -7.154689 2.158599 1.756184

C -3.060130 4.111503 2.668443

O -3.588242 5.215105 2.630930

O -2.047571 1.376242 2.673073

H -1.852484 0.427584 2.716792

Au -1.027100 4.004096 2.970457

P -0.709614 4.650167 0.688042

C 0.982188 4.367924 0.064821

C 1.235041 4.849841 -1.361259

C -1.850228 3.859581 -0.501380

C -1.592736 2.362958 -0.663811

C -1.055807 6.437912 0.563346

C -0.098361 7.286713 1.398561

H 1.171249 3.292967 0.165451

H 1.654921 4.851945 0.780509

H 2.273597 4.646185 -1.643699

H 1.072067 5.928263 -1.459529

H 0.589971 4.342093 -2.086163

H -1.765382 4.384809 -1.460566

H -2.862818 4.041950 -0.124309

H -2.373592 1.911043 -1.284281

H -1.590789 1.854162 0.304422

H -0.629461 2.172499 -1.148634

H -2.086846 6.560166 0.916055

H -1.031729 6.727061 -0.494274

H -0.384638 8.341951 1.339234

H 0.935074 7.200840 1.047173

H -0.120988 6.990204 2.452239

C 0.983080 3.979658 3.391074

N 2.134329 3.970307 3.590350

H -1.402296 3.669946 4.505400

Structure: 6

Symbol X Y Z

C -0.702685 1.303525 -0.401307

C -1.053108 2.651097 -0.408686

C -2.345681 3.040579 -0.015534

C -3.272567 2.059169 0.402406

C -2.891902 0.704920 0.404415

C -1.620069 0.319088 0.002731

H 0.295678 1.015194 -0.714964

H -0.340807 3.409239 -0.710533

H -3.626980 -0.022900 0.732062

H -1.335261 -0.726860 0.002503

C -4.605250 2.446257 0.850714

O -5.442739 1.648426 1.324981

H -4.849678 3.512420 0.759342

O -2.739880 4.345206 -0.022072

H -1.952781 4.987903 -0.034906

Au -0.204225 5.418095 3.624153

P 1.400264 6.660030 5.018153

C 2.847135 5.613890 5.649212

C 3.854117 6.335557 6.538598

C 2.263910 8.118890 4.185491

C 2.996818 7.684547 2.918073

C 0.708887 7.436393 6.593637

C 0.136957 6.373545 7.528702

H 3.329555 5.208185 4.753398

H 2.396585 4.764245 6.173282

H 4.648215 5.647825 6.851764

H 3.383417 6.730198 7.445248

H 4.330324 7.170513 6.014381

H 2.941901 8.592618 4.904628

H 1.477127 8.842946 3.948263

H 3.414783 8.552246 2.396421

H 2.313074 7.169045 2.235004

H 3.824265 7.003446 3.146898

H -0.072098 8.130892 6.264972

H 1.491046 8.026574 7.084434

H -0.384651 6.834229 8.374490

H 0.926823 5.731577 7.934058

H -0.573950 5.733594 6.995236

P -2.544457 6.269479 4.011873

C -3.194924 6.736164 5.729763

C -4.569281 7.398551 5.753769

H -4.877837 7.604797 6.785456

H -4.565844 8.350796 5.213437

H -5.334021 6.758468 5.301355

H -3.194058 5.811323 6.317119

H -2.436279 7.388124 6.175567

C -3.943848 5.235809 3.274705

C -4.292307 4.022561 4.131835

H -5.004403 3.371899 3.614230

H -3.399349 3.431197 4.353631

H -4.740728 4.317414 5.086923

H -4.818904 5.880334 3.129765

H -3.587307 4.934839 2.283504

C -2.726498 7.872142 3.030179

C -1.771221 8.953648 3.524692

H -1.837881 9.849402 2.897602

H -1.992879 9.250470 4.556531

H -0.739761 8.587667 3.487624

H -2.495539 7.601439 1.994446

H -3.770108 8.203695 3.068749

C -0.234522 5.760679 1.551299

N -0.615212 5.831635 0.429176

P 0.105796 3.023385 4.162437

C -1.040473 1.816767 3.268488

C -0.810527 0.339171 3.561747

C 1.822992 2.385119 3.701828

C 2.115764 2.599377 2.218366

C -0.030355 2.513148 5.976322

C -1.440061 2.724620 6.521514

H -0.916681 2.029902 2.203444

H -2.062632 2.112876 3.518493

H -1.536745 -0.264322 3.007078

H -0.927153 0.108407 4.626311

H 0.188457 0.015710 3.250694

H 1.896377 1.327191 3.978780

H 2.533786 2.939467 4.323380

H 3.141486 2.299070 1.977411

H 1.988679 3.653235 1.948238

H 1.437040 2.013474 1.589678

H 0.690254 3.132728 6.521067

H 0.285475 1.468755 6.080480

H -1.474113 2.537248 7.600214

H -2.159272 2.051226 6.043232

H -1.774743 3.752335 6.343145

Structure: TS_6/7_

Symbol X Y Z

C -2.142820 2.216560 -3.295904

C -1.445311 1.037701 -3.072659

C -2.137063 -0.182197 -2.978984

C -3.542865 -0.193694 -3.128859

C -4.223183 1.015865 -3.351239

C -3.539241 2.218626 -3.431330

H -1.592339 3.151733 -3.365493

H -0.368103 1.038151 -2.960396

H -5.304041 0.975584 -3.455262

H -4.073663 3.148356 -3.603084

C -4.293854 -1.450006 -3.032259

O -5.515759 -1.528207 -3.082801

H -3.687245 -2.363995 -2.906991

O -1.506947 -1.338301 -2.723681

H -0.562183 -1.164325 -2.419949

Au 0.549818 -0.116729 0.336291

P 1.544629 2.080153 0.187569

C 3.377910 2.179911 0.056875

C 3.997110 3.570525 0.167238

C 0.976370 3.209135 -1.155210

C 1.636020 2.905481 -2.500996

C 1.178320 3.019485 1.727040

C 1.663020 2.281602 2.973075

H 3.631937 1.710689 -0.899620

H 3.782137 1.518293 0.828851

H 5.078443 3.517955 -0.005775

H 3.844725 4.003978 1.161341

H 3.579825 4.266102 -0.568599

H 1.153530 4.250940 -0.858208

H -0.107772 3.074664 -1.228199

H 1.112701 3.426307 -3.310694

H 1.632547 1.833626 -2.722037

H 2.678949 3.237892 -2.518447

H 0.091602 3.147923 1.765934

H 1.618291 4.022171 1.662101

H 1.373071 2.819414 3.882833

H 2.753949 2.177828 2.981833

H 1.231273 1.274977 3.015425

P -1.783015 -0.152288 0.829507

C -1.990763 -0.022139 2.657036

C -3.419242 -0.076215 3.189596

H -3.420085 -0.005121 4.283632

H -4.028934 0.748498 2.806128

H -3.916137 -1.014128 2.919854

H -1.382378 -0.818335 3.096986

H -1.499466 0.914813 2.946557

C -2.741033 -1.642091 0.338632

C -2.219226 -2.946510 0.924713

H -2.755495 -3.799667 0.494634

H -1.154478 -3.070428 0.703840

H -2.348640 -2.987293 2.011651

H -3.796756 -1.486703 0.592612

H -2.666505 -1.675637 -0.752002

C -2.899798 1.169469 0.201851

C -2.524402 2.576369 0.642792

H -3.200909 3.310375 0.191188

H -2.578008 2.697633 1.730626

H -1.508529 2.823515 0.325218

H -2.876688 1.102970 -0.887613

H -3.923357 0.924134 0.510763

C 1.150012 -0.826498 -2.013959

N 2.106403 -0.815956 -2.697376

P 2.045053 -1.780204 1.238703

C 2.005594 -3.376512 0.329927

C 2.967289 -4.458740 0.811306

C 3.830470 -1.327346 1.243467

C 4.431047 -1.191090 -0.152262

C 1.834533 -2.240608 3.012062

C 0.575483 -3.045527 3.307412

H 2.188206 -3.113696 -0.718329

H 0.968512 -3.728899 0.370055

H 2.852137 -5.364414 0.204517

H 2.783163 -4.736032 1.855209

H 4.011612 -4.140096 0.728879

H 4.381351 -2.070749 1.834436

H 3.895188 -0.381796 1.795340

H 5.429474 -0.741731 -0.099909

H 3.804455 -0.579243 -0.804567

H 4.530396 -2.168488 -0.635710

H 1.827590 -1.297287 3.571968

H 2.726552 -2.793532 3.333277

H 0.464277 -3.209786 4.385175

H 0.600306 -4.026562 2.821478

H -0.316757 -2.527259 2.950759

Structure: 7

Symbol X Y Z

C -2.303611 2.252680 -3.525143

C -1.662518 1.021948 -3.500690

C -2.392715 -0.155622 -3.240610

C -3.789373 -0.056698 -3.024395

C -4.413633 1.201586 -3.062604

C -3.685893 2.355784 -3.303274

H -1.721106 3.149317 -3.723783

H -0.595617 0.948642 -3.677579

H -5.485808 1.239433 -2.888385

H -4.176143 3.324773 -3.328520

C -4.576550 -1.255109 -2.725562

O -5.771737 -1.247562 -2.447798

H -4.022904 -2.209421 -2.756224

O -1.798857 -1.347800 -3.172660

H -0.790001 -1.244543 -2.897470

Au 0.663920 -0.071913 0.702084

P 1.628330 2.102669 0.317612

C 3.432800 2.159677 -0.032063

C 4.073497 3.542799 -0.105399

C 0.902741 3.154726 -1.010703

C 1.375942 2.750112 -2.406021

C 1.443846 3.133653 1.830221

C 2.047458 2.458756 3.060174

H 3.563849 1.609197 -0.969902

H 3.917351 1.551544 0.738434

H 5.128979 3.456400 -0.389104

H 4.034779 4.059111 0.859723

H 3.585068 4.183301 -0.847354

H 1.134596 4.207981 -0.805377

H -0.183771 3.041488 -0.935444

H 0.791701 3.271371 -3.172184

H 1.274277 1.672349 -2.559961

H 2.427965 3.008808 -2.564242

H 0.369274 3.290522 1.976503

H 1.889971 4.120972 1.659247

H 1.873987 3.060050 3.959757

H 3.129810 2.323960 2.954755

H 1.601322 1.469782 3.217149

P -1.701623 -0.193749 0.815182

C -2.284473 -0.172857 2.562022

C -3.794145 -0.277569 2.761438

H -4.042163 -0.218984 3.827663

H -4.331000 0.529527 2.252071

H -4.185344 -1.227736 2.383520

H -1.770269 -0.989887 3.078295

H -1.891342 0.751635 3.001967

C -2.498914 -1.668012 0.073283

C -2.057586 -2.980411 0.708824

H -2.492180 -3.829810 0.170503

H -0.968545 -3.077873 0.672916

H -2.368215 -3.055271 1.756950

H -3.587069 -1.544203 0.133879

H -2.228014 -1.656664 -0.987238

C -2.631838 1.187341 0.039801

C -2.473629 2.514279 0.772190

H -2.886592 3.332367 0.171852

H -2.991195 2.513982 1.737324

H -1.419328 2.737974 0.962655

H -2.249908 1.269821 -0.981433

H -3.689116 0.910195 -0.041727

C 0.718755 -1.035303 -2.307631

N 1.819368 -0.793956 -1.972401

P 2.178403 -1.763606 1.442132

C 2.141070 -3.303720 0.444835

C 3.057662 -4.430473 0.911823

C 3.960643 -1.301264 1.476064

C 4.577146 -1.191717 0.082677

C 1.923291 -2.310099 3.181114

C 0.588078 -3.009317 3.407587

H 2.372450 -2.985412 -0.577530

H 1.097215 -3.634843 0.419871

H 2.983175 -5.284949 0.229215

H 2.791116 -4.785493 1.913441

H 4.107640 -4.119959 0.935340

H 4.509092 -2.025907 2.091933

H 4.006328 -0.341033 2.004108

H 5.546016 -0.681248 0.127842

H 3.921874 -0.649952 -0.604055

H 4.744373 -2.183419 -0.350602

H 1.993447 -1.405838 3.798470

H 2.758245 -2.960177 3.471678

H 0.439215 -3.231047 4.470308

H 0.530200 -3.954821 2.857937

H -0.241243 -2.381928 3.070484

Structure: TS_7/8_

Symbol X Y Z

C -2.823734 6.939799 -0.153321

C -2.180346 5.712933 -0.169260

C -2.881009 4.514063 0.132269

C -4.274835 4.624720 0.419585

C -4.902048 5.883212 0.419898

C -4.193995 7.041390 0.147210

H -2.256674 7.839341 -0.384803

H -1.126553 5.648807 -0.419395

H -5.964670 5.916992 0.648545

H -4.687165 8.009424 0.150940

C -5.045875 3.428281 0.739487

O -6.225285 3.424279 1.089689

H -4.494311 2.475586 0.653738

O -2.270296 3.353792 0.175259

H -1.078630 3.455759 0.522628

Au 0.189221 4.601518 4.134331

P 1.152520 6.775951 3.736147

C 2.956804 6.844509 3.382476

C 3.585241 8.232627 3.296520

C 0.417452 7.821279 2.410160

C 0.858525 7.388085 1.013316

C 0.964557 7.800005 5.252469

C 1.573262 7.125504 6.480216

H 3.094673 6.290488 2.447575

H 3.447881 6.246261 4.156811

H 4.643650 8.151865 3.022567

H 3.533925 8.761258 4.254225

H 3.097182 8.857979 2.541601

H 0.670387 8.873113 2.595993

H -0.669005 7.726944 2.506466

H 0.276953 7.916092 0.250319

H 0.719058 6.313034 0.870977

H 1.915310 7.613328 0.836440

H -0.110635 7.950625 5.399823

H 1.404898 8.790080 5.083236

H 1.398677 7.725391 7.380412

H 2.655941 6.995229 6.373241

H 1.131091 6.134867 6.637868

P -2.181917 4.477988 4.195761

C -2.804809 4.475405 5.928575

C -4.319358 4.367751 6.086337

H -4.595425 4.414822 7.146183

H -4.842932 5.179772 5.571202

H -4.699551 3.421665 5.687789

H -2.304248 3.650525 6.445958

H -2.424109 5.393957 6.391278

C -2.950895 3.009637 3.416876

C -2.517556 1.693540 4.050289

H -2.931121 0.847568 3.490571

H -1.427279 1.603606 4.043995

H -2.856256 1.604510 5.088644

H -4.040269 3.127991 3.456563

H -2.661402 3.034459 2.360263

C -3.085692 5.870181 3.413448

C -2.949621 7.185490 4.170717

H -3.340419 8.012651 3.568093

H -3.500874 7.172148 5.116940

H -1.902479 7.404992 4.402259

H -2.676504 5.965346 2.403352

H -4.139505 5.594602 3.296507

C 0.163380 3.572181 0.986674

N 1.259656 3.731755 1.360865

P 1.700158 2.896609 4.847948

C 1.647259 1.347664 3.864537

C 2.571252 0.225683 4.328909

C 3.482846 3.353874 4.857616

C 4.071415 3.474780 3.453416

C 1.450958 2.368562 6.592648

C 0.111458 1.680181 6.829105

H 1.864091 1.652833 2.835046

H 0.603648 1.015519 3.860031

H 2.480144 -0.637943 3.660120

H 2.324646 -0.114434 5.340654

H 3.622184 0.533669 4.325567

H 4.041132 2.622194 5.455762

H 3.540257 4.309312 5.392995

H 5.044871 3.977135 3.483370

H 3.407535 4.032087 2.787635

H 4.220171 2.487685 3.003554

H 1.528886 3.278206 7.200959

H 2.283156 1.716300 6.885838

H -0.036598 1.471925 7.894554

H 0.047015 0.728581 6.290928

H -0.714589 2.309051 6.486144

Structure: 8

Symbol X Y Z

C -2.323598 -0.109934 2.647552

C -2.183726 1.261783 2.738736

C -1.152137 1.965804 2.051135

C -0.243462 1.162842 1.280365

C -0.411383 -0.233922 1.203046

C -1.437360 -0.880688 1.868735

H -3.128119 -0.601551 3.193025

H -2.845313 1.851385 3.364217

H 0.305772 -0.792738 0.605757

H -1.555407 -1.958627 1.800872

C 0.898636 1.780825 0.624848

O 1.739701 1.188940 -0.052724

H 1.002564 2.869424 0.784142

O -1.050477 3.253938 2.142168

H -1.030849 4.071131 0.712199

Au -1.783565 4.470170 4.464771

P -0.524208 6.470845 4.065881

C 0.861367 6.705550 5.256998

C 1.657563 8.001913 5.142774

C 0.259278 6.684897 2.421089

C 1.316967 5.621345 2.138933

C -1.525294 7.999868 4.258344

C -2.123939 8.121551 5.657795

H 1.515785 5.836484 5.125307

H 0.423117 6.602452 6.256874

H 2.457926 8.024765 5.891533

H 1.027250 8.882102 5.308233

H 2.125552 8.103280 4.158032

H 0.677485 7.697162 2.352887

H -0.546215 6.623895 1.680854

H 1.694298 5.721495 1.115608

H 0.886977 4.621072 2.247739

H 2.171639 5.710132 2.818802

H -2.316468 7.939720 3.501586

H -0.915403 8.877892 4.012136

H -2.839904 8.949437 5.706942

H -1.348651 8.308195 6.408878

H -2.647315 7.201412 5.941555

P -4.070172 4.514310 3.827878

C -4.947958 6.008365 4.449529

C -6.416137 6.148613 4.058337

H -6.818686 7.099101 4.427329

H -6.549560 6.133118 2.971310

H -7.028118 5.346312 4.483172

H -4.833025 6.003847 5.539919

H -4.366451 6.865254 4.093139

C -5.176136 3.135264 4.339012

C -5.240456 2.970826 5.853557

H -5.812195 2.076843 6.125944

H -4.234581 2.874832 6.274600

H -5.719641 3.831469 6.333528

H -6.173874 3.303283 3.915262

H -4.786654 2.225612 3.869620

C -4.285245 4.558228 2.006497

C -3.789465 5.863781 1.393287

H -3.798473 5.806758 0.300710

H -4.411862 6.714827 1.690948

H -2.760910 6.070260 1.704788

H -3.699262 3.714228 1.625133

H -5.337693 4.376617 1.756112

C -1.028930 4.662535 -0.241881

N -1.026663 5.295725 -1.214282

P -0.720680 2.979044 5.985707

C -1.418862 1.286162 6.152508

C -0.782595 0.373087 7.195936

C 1.072526 2.700095 5.704557

C 1.360385 2.104008 4.329874

C -0.746633 3.674605 7.687120

C -2.159039 3.945782 8.197460

H -1.354530 0.844490 5.152094

H -2.489869 1.415981 6.347184

H -1.283752 -0.601908 7.197049

H -0.863560 0.785252 8.207688

H 0.277111 0.193965 6.986721

H 1.472349 2.070907 6.509716

H 1.548382 3.682899 5.802700

H 2.439500 2.008803 4.166467

H 0.942719 2.736582 3.541852

H 0.916997 1.109219 4.216960

H -0.175284 4.609274 7.636685

H -0.202705 3.003921 8.363640

H -2.131446 4.427610 9.181339

H -2.734076 3.018729 8.299025

H -2.701351 4.602985 7.508336

Structure: 9

Symbol X Y Z

C 0.615981 4.441833 -1.052684

C -0.287690 5.152430 -0.290307

C -1.004299 6.283930 -0.811597

C -0.680469 6.654799 -2.169990

C 0.241767 5.901916 -2.923495

C 0.888796 4.800560 -2.391792

H 1.122332 3.581253 -0.616106

H -0.516292 4.852627 0.728188

H 0.428086 6.215761 -3.948838

H 1.597015 4.227109 -2.983721

C -1.347785 7.794076 -2.765473

O -1.206856 8.191200 -3.926764

H -2.037846 8.328272 -2.085981

O -1.888869 6.896536 -0.119597

Au -3.534868 5.267079 1.179075

P -5.177148 7.007476 1.084117

C -5.854765 7.521934 2.718405

C -6.905220 8.628548 2.703975

C -4.575820 8.564774 0.324402

C -3.515037 9.260243 1.172387

C -6.680753 6.607030 0.104368

C -7.501298 5.486930 0.740347

H -4.988908 7.817028 3.322698

H -6.249739 6.611535 3.185510

H -7.229091 8.864479 3.724546

H -7.794586 8.335731 2.135889

H -6.513164 9.549764 2.260286

H -5.432263 9.222269 0.127541

H -4.140272 8.276146 -0.638141

H -3.141299 10.150329 0.653733

H -2.668754 8.587778 1.332119

H -3.911674 9.584144 2.141628

H -6.318248 6.308885 -0.886187

H -7.289987 7.509156 -0.034235

H -8.277954 5.131575 0.053918

H -7.998384 5.826295 1.655553

H -6.866506 4.633633 1.004190

P -3.559691 3.592083 -0.495626

C -5.240953 2.859053 -0.682255

C -5.382847 1.701790 -1.666738

H -6.421855 1.352878 -1.695098

H -5.108601 1.998278 -2.684354

H -4.756921 0.848379 -1.385469

H -5.558369 2.555507 0.322566

H -5.897560 3.689141 -0.965821

C -2.473276 2.136468 -0.222639

C -2.847487 1.369131 1.041617

H -2.089633 0.615486 1.283108

H -2.934851 2.050983 1.894942

H -3.807630 0.852718 0.932473

H -2.498806 1.485441 -1.105541

H -1.458219 2.541381 -0.148492

C -3.113640 4.133956 -2.188728

C -3.858992 5.394654 -2.613115

H -3.520953 5.725321 -3.600707

H -4.941495 5.227432 -2.668048

H -3.667801 6.202594 -1.899458

H -2.034734 4.317288 -2.181578

H -3.294725 3.304737 -2.883663

P -2.439408 5.201481 3.290139

C -1.093806 3.970887 3.560065

C -0.408116 3.985238 4.923398

C -1.695147 6.798182 3.808273

C -0.519234 7.202890 2.921085

C -3.645538 4.855862 4.633057

C -4.432892 3.569408 4.395853

H -0.363395 4.138765 2.760383

H -1.534410 2.988311 3.356854

H 0.365736 3.209842 4.966317

H -1.113989 3.789387 5.737611

H 0.078350 4.945878 5.121800

H -1.406979 6.744084 4.866127

H -2.499906 7.538887 3.729511

H -0.164725 8.204600 3.190058

H -0.816829 7.207914 1.866375

H 0.324747 6.513135 3.035665

H -4.324084 5.716813 4.652377

H -3.128499 4.833861 5.600155

H -5.193781 3.428860 5.172018

H -3.780181 2.689287 4.409051

H -4.934675 3.598732 3.421747

Structure: TS_9/10_

Symbol X Y Z

C 0.443782 4.756354 -1.605256

C -0.244747 5.493184 -0.642333

C -0.978426 6.615742 -1.025838

C -1.041358 7.015964 -2.368895

C -0.328107 6.277834 -3.312982

C 0.412519 5.152818 -2.944509

H 1.010240 3.877468 -1.306256

H -0.224060 5.215659 0.407211

H -0.391262 6.602981 -4.348066

H 0.953775 4.584608 -3.696509

C -1.949733 8.142429 -2.841604

O -1.897469 8.514360 -4.046247

H -2.953456 8.036090 -2.369456

O -1.643917 7.307599 -0.056921

Au -3.585892 5.347265 1.057508

P -5.196317 7.123633 1.042076

C -5.898663 7.462342 2.709499

C -6.975842 8.540671 2.783866

C -4.591763 8.754886 0.460465

C -3.591675 9.392565 1.422490

C -6.674429 6.802421 0.000145

C -7.495224 5.623614 0.519027

H -5.045865 7.720614 3.348206

H -6.275758 6.502977 3.083982

H -7.312280 8.673494 3.818575

H -7.853210 8.279865 2.182933

H -6.603914 9.508085 2.430806

H -5.451826 9.414950 0.294230

H -4.131154 8.583940 -0.519086

H -3.124053 10.265834 0.957796

H -2.795629 8.690599 1.688247

H -4.078030 9.719395 2.347925

H -6.295192 6.597019 -1.007105

H -7.287081 7.710048 -0.064084

H -8.253828 5.324717 -0.212533

H -8.013809 5.875478 1.450239

H -6.857573 4.755189 0.719302

P -3.532658 3.687945 -0.637620

C -5.162495 2.839547 -0.754616

C -5.262701 1.704158 -1.769624

H -6.263329 1.257655 -1.740107

H -5.091987 2.059994 -2.790704

H -4.539897 0.907007 -1.566637

H -5.405728 2.487286 0.255037

H -5.890910 3.624453 -0.984465

C -2.331968 2.321151 -0.384136

C -2.673355 1.469393 0.834949

H -1.843052 0.802796 1.092453

H -2.885816 2.099574 1.705231

H -3.556880 0.847060 0.657960

H -2.277704 1.707816 -1.292124

H -1.356525 2.804579 -0.264672

C -3.187243 4.241229 -2.346436

C -4.131417 5.342439 -2.812436

H -3.809913 5.739685 -3.780402

H -5.160788 4.980685 -2.917899

H -4.134235 6.172220 -2.098567

H -2.153330 4.595636 -2.354777

H -3.230824 3.369097 -3.010188

P -2.418345 5.209662 3.146602

C -1.013287 4.027971 3.288153

C -0.366036 3.892184 4.663542

C -1.752884 6.771744 3.850368

C -0.517184 7.284457 3.113469

C -3.616281 4.676459 4.434941

C -4.335112 3.377418 4.079786

H -0.275315 4.347296 2.542876

H -1.387000 3.060295 2.937747

H 0.465064 3.178843 4.620765

H -1.075908 3.524119 5.411608

H 0.037221 4.845350 5.020655

H -1.543617 6.619121 4.916982

H -2.567109 7.502709 3.785465

H -0.223796 8.266195 3.501316

H -0.707564 7.384205 2.041055

H 0.336210 6.610366 3.246368

H -4.337996 5.496889 4.525187

H -3.103764 4.595382 5.401127

H -5.090662 3.133339 4.834909

H -3.638495 2.533632 4.024888

H -4.836722 3.462173 3.108708

C -1.476466 9.548029 -1.696921

N -1.316067 10.635273 -1.289675

H -1.743706 8.234524 -0.379534

Structure: 7'

Symbol X Y Z

C -4.073545 -0.842321 -6.028088

C -3.957360 -0.122211 -4.849152

C -2.888231 -0.359843 -3.964026

C -1.953378 -1.376316 -4.285762

C -2.082953 -2.079901 -5.499664

C -3.125847 -1.821804 -6.371295

H -4.904702 -0.636302 -6.698671

H -4.673888 0.651546 -4.588393

H -1.345643 -2.847725 -5.718503

H -3.218630 -2.374075 -7.302082

C -0.914942 -1.779699 -3.339513

O -0.043417 -2.611260 -3.580103

H -0.958128 -1.315598 -2.337959

O -2.818291 0.375983 -2.852822

H -1.830645 0.634192 -2.577205

Au 0.390818 0.244084 0.822761

P 1.846093 1.926525 1.743735

C 3.353550 1.258839 2.567233

C 4.290876 2.281484 3.202987

C 2.489810 3.163844 0.552122

C 3.492349 2.565867 -0.430646

C 1.079065 2.951570 3.064081

C 0.724279 2.125248 4.298712

H 3.882221 0.671137 1.808210

H 2.999538 0.538016 3.313903

H 5.159736 1.780411 3.645675

H 3.795351 2.845807 3.999952

H 4.663880 3.000235 2.465864

H 2.923781 4.004677 1.108053

H 1.616937 3.531503 0.002551

H 3.788729 3.317226 -1.171007

H 3.038395 1.731746 -0.971213

H 4.402105 2.217483 0.071322

H 0.179250 3.389646 2.619836

H 1.745717 3.782509 3.326931

H 0.093536 2.699645 4.986356

H 1.621654 1.821490 4.848368

H 0.181290 1.214507 4.022112

P -1.953219 0.514874 1.038922

C -2.442462 0.654254 2.810296

C -3.938219 0.709918 3.107689

H -4.107005 0.805295 4.186791

H -4.417000 1.567303 2.623424

H -4.454182 -0.195791 2.772698

H -1.973846 -0.191719 3.327515

H -1.941645 1.552611 3.187995

C -3.004482 -0.841040 0.387711

C -2.733415 -2.168713 1.087236

H -3.243258 -2.991947 0.574835

H -1.660225 -2.386925 1.097393

H -3.078417 -2.157573 2.127326

H -4.060492 -0.554951 0.471840

H -2.781156 -0.903007 -0.682747

C -2.678268 2.003835 0.250199

C -1.927792 3.285493 0.590014

H -2.379036 4.141465 0.075592

H -1.948479 3.497435 1.665413

H -0.884853 3.206616 0.267432

H -2.645466 1.821846 -0.827402

H -3.736370 2.068660 0.532012

C -0.411940 1.206823 -2.100301

N 0.553738 1.732711 -1.687240

P 1.655836 -1.722806 0.344300

C 0.861781 -3.176103 -0.451002

C 1.720549 -4.429280 -0.599701

C 3.171833 -1.439582 -0.651791

C 2.855017 -1.052718 -2.094878

C 2.318489 -2.375464 1.930431

C 1.215583 -2.650886 2.949964

H 0.525278 -2.842902 -1.438654

H -0.042892 -3.392306 0.127576

H 1.150400 -5.209957 -1.116358

H 2.034833 -4.835739 0.367741

H 2.618539 -4.235488 -1.195755

H 3.808467 -2.332419 -0.606650

H 3.714761 -0.630182 -0.149663

H 3.776959 -0.806206 -2.634041

H 2.191999 -0.182406 -2.131691

H 2.358787 -1.868169 -2.630189

H 3.008071 -1.614949 2.312570

H 2.912994 -3.275953 1.734878

H 1.638942 -2.991348 3.901889

H 0.525826 -3.425722 2.596862

H 0.629087 -1.743977 3.138000

Structure: TS_7'/10_

Symbol X Y Z

C -3.551285 2.649952 -2.722430

C -3.951339 3.745114 -1.959751

C -3.290039 4.042185 -0.764877

C -2.227783 3.236334 -0.318626

C -1.829914 2.159595 -1.110017

C -2.478666 1.857095 -2.305616

H -4.072870 2.423135 -3.649129

H -4.766693 4.388027 -2.279789

H -1.001260 1.556187 -0.746034

H -2.160474 1.008242 -2.906393

C -1.578761 3.447017 1.030125

O -0.571959 2.757855 1.362620

H -2.336294 3.662231 1.810693

O -3.706376 5.123371 -0.053713

H -2.947422 5.431679 0.491265

Au 0.911658 0.460739 0.808209

P 2.157178 1.073604 2.762015

C 3.981753 1.074633 2.513261

C 4.833937 1.432580 3.727183

C 1.772577 2.734199 3.432909

C 2.184957 3.862763 2.493254

C 1.913726 -0.040483 4.203756

C 2.414922 -1.456415 3.928580

H 4.168926 1.772040 1.688398

H 4.240831 0.080115 2.129993

H 4.684345 0.725434 4.550132

H 4.601060 2.435464 4.100020

H 2.241321 2.834416 4.420322

H 0.685350 2.754537 3.562166

H 1.667412 3.760702 1.537017

H 3.267714 3.882563 2.322411

H 0.834827 -0.041068 4.399532

H 2.401218 0.383357 5.090800

H 3.508579 -1.489545 3.876672

H 2.025695 -1.833635 2.976029

P -1.034034 -0.862368 1.061696

C -0.758148 -2.344686 2.118514

C -1.950521 -3.276336 2.313631

H -2.796025 -2.759297 2.779298

H -2.295935 -3.699709 1.364644

H 0.089616 -2.882537 1.677423

H -0.401485 -1.964098 3.081849

C -1.759196 -1.546877 -0.481096

C -0.785589 -2.462359 -1.217014

H 0.183407 -1.968380 -1.351356

H -0.609829 -3.392223 -0.664590

H -2.697221 -2.067156 -0.250606

H -2.018949 -0.680098 -1.098060

C -2.448521 0.008376 1.836096

C -2.106136 0.575787 3.210258

H -1.833624 -0.213083 3.921202

H -1.275692 1.282668 3.119796

H -2.714611 0.817657 1.147521

H -3.304520 -0.676631 1.880203

C -1.033215 5.298280 0.910884

N -0.323509 6.224375 1.021198

P 2.044386 1.033689 -1.202715

C 1.178900 0.738807 -2.799637

C 1.941458 1.095158 -4.072375

C 2.585250 2.783256 -1.298872

C 1.400704 3.745194 -1.356524

C 3.615313 0.091764 -1.339480

C 3.402129 -1.417724 -1.253313

H 0.245980 1.309044 -2.739395

H 0.885998 -0.317340 -2.800640

H 2.866890 0.517781 -4.171302

H 2.200579 2.158505 -4.103325

H 3.256473 2.912338 -2.157533

H 3.178398 2.964877 -0.394525

H 0.714966 3.568433 -0.522621

H 0.834184 3.631716 -2.287208

H 4.243281 0.434152 -0.508679

H 4.131570 0.368686 -2.266720

H 2.804077 -1.788627 -2.093245

H 2.878940 -1.682513 -0.327120

H 1.325158 0.882320 -4.953661

H 1.743034 4.783344 -1.297749

H 4.360892 -1.948268 -1.269929

H 1.895492 4.830946 2.913851

H 5.898834 1.415219 3.467100

H 2.099713 -2.145500 4.720056

H -1.675752 -4.112972 2.966567

H -2.966868 1.111730 3.626088

H -1.172074 -2.731223 -2.206328

Structure: 10

Symbol X Y Z

C -3.864315 0.925888 -3.224041

C -4.509196 1.377069 -2.073167

C -3.761979 1.917837 -1.024045

C -2.364249 1.978839 -1.103525

C -1.740755 1.537744 -2.267839

C -2.473960 1.014581 -3.331140

H -4.452676 0.509622 -4.038035

H -5.590543 1.330519 -1.977406

H -0.658091 1.609885 -2.280534

H -1.969583 0.667356 -4.229265

C -1.474907 2.465889 0.055020

O -0.154713 2.292724 -0.133888

H -1.862262 1.964567 0.975658

O -4.454757 2.342498 0.081147

H -3.942804 3.013150 0.561852

Au 0.395811 -0.157987 0.135677

P 1.643175 0.298620 2.155501

C 3.440604 0.621634 1.900769

C 4.301943 0.770987 3.151213

C 1.121787 1.683599 3.247990

C 1.339964 3.052326 2.605006

C 1.627193 -1.146656 3.293735

C 2.208580 -2.397407 2.639186

H 3.491641 1.528055 1.286610

H 3.812307 -0.193386 1.268509

H 5.342787 0.978342 2.875691

H 4.297949 -0.140521 3.758474

H 3.958039 1.597193 3.782256

H 1.645524 1.595958 4.209229

H 0.056643 1.529755 3.452674

H 0.899814 3.839672 3.227985

H 0.872113 3.078830 1.612734

H 2.407566 3.277433 2.497640

H 0.575297 -1.304426 3.560993

H 2.158822 -0.902455 4.221954

H 2.060388 -3.279501 3.272378

H 3.284915 -2.291979 2.463151

H 1.730520 -2.586087 1.671379

P -1.632078 -1.347430 0.429944

C -1.261196 -2.962461 1.240368

C -2.446793 -3.880960 1.519678

H -2.102659 -4.821174 1.966529

H -3.156737 -3.426286 2.218504

H -2.992009 -4.131774 0.603517

H -0.528362 -3.461052 0.595243

H -0.726934 -2.725675 2.166878

C -2.599929 -1.814873 -1.057044

C -1.827780 -2.725856 -2.003182

H -2.354223 -2.830958 -2.958319

H -0.834859 -2.313779 -2.206743

H -1.690385 -3.728771 -1.583482

H -3.544394 -2.277777 -0.744372

H -2.850271 -0.873207 -1.554388

C -2.942220 -0.624387 1.496673

C -2.444827 -0.254978 2.888297

H -3.230951 0.247304 3.462736

H -2.130066 -1.138704 3.455312

H -1.587840 0.422125 2.822093

H -3.320977 0.259663 0.974464

H -3.777667 -1.333318 1.551333

C -1.813267 3.915176 0.295218

N -2.058782 5.032513 0.515647

P 1.871913 -0.336795 -1.733641

C 1.187021 -0.473867 -3.440315

C 2.193898 -0.667141 -4.570521

C 3.078088 1.041528 -1.881447

C 2.390697 2.372985 -2.183119

C 2.958569 -1.811837 -1.569132

C 2.173818 -3.120566 -1.576849

H 0.600263 0.436219 -3.601975

H 0.460813 -1.292601 -3.424775

H 1.673872 -0.722051 -5.534196

H 2.766068 -1.593680 -4.453117

H 2.904634 0.163765 -4.628670

H 3.833106 0.793493 -2.638841

H 3.596966 1.089755 -0.918228

H 3.107974 3.199021 -2.111108

H 1.565401 2.558243 -1.484311

H 1.981391 2.384951 -3.200341

H 3.483813 -1.692616 -0.613869

H 3.720602 -1.801918 -2.358254

H 2.832315 -3.973133 -1.376029

H 1.690002 -3.294409 -2.544375

H 1.390469 -3.104615 -0.811277

Structure: 11

Symbol X Y Z

C 2.295177 1.296187 -4.716070

C 1.036389 1.543462 -5.265841

C -0.104346 1.030925 -4.645153

C 0.004193 0.283825 -3.465042

C 1.266160 0.042393 -2.936231

C 2.416173 0.538196 -3.551843

H 3.179121 1.693893 -5.208205

H 0.940760 2.128921 -6.178624

H 1.307076 -0.540238 -2.022342

H 3.396305 0.332785 -3.130059

C -1.226435 -0.293253 -2.760860

O -0.935098 -1.100246 -1.698080

H -1.825820 -0.816544 -3.535950

O -1.361818 1.229158 -5.137487

H -1.320443 1.754620 -5.950029

Au -0.075217 -0.085669 0.628139

P 1.001070 2.035012 0.507675

C 2.686480 2.140440 -0.223906

C 3.413717 3.474786 -0.079786

C 0.097365 3.436312 -0.279991

C 0.244632 3.480043 -1.798591

C 1.275633 2.654769 2.221257

C 2.161090 1.731679 3.051577

H 2.573098 1.867539 -1.278488

H 3.269356 1.335883 0.236987

H 4.361050 3.449372 -0.631223

H 3.649508 3.697511 0.966107

H 2.825329 4.309266 -0.475295

H 0.439393 4.376431 0.171939

H -0.956688 3.307610 -0.012313

H -0.432766 4.228729 -2.222970

H -0.001886 2.518064 -2.250605

H 1.264326 3.737522 -2.101803

H 0.291475 2.748602 2.690492

H 1.699605 3.664790 2.165677

H 2.269570 2.113261 4.073144

H 3.165175 1.638338 2.623191

H 1.725018 0.730179 3.106548

P -2.376257 -0.133724 1.351244

C -2.494370 -0.845065 3.048735

C -3.889511 -0.954779 3.655898

H -3.835917 -1.388459 4.661548

H -4.371920 0.024383 3.744978

H -4.541738 -1.596664 3.054464

H -2.014693 -1.829320 2.998270

H -1.840834 -0.231212 3.680786

C -3.568312 -1.116046 0.357263

C -3.222328 -2.593428 0.243040

H -3.974127 -3.115522 -0.359879

H -2.256166 -2.710122 -0.249391

H -3.183527 -3.085816 1.221651

H -4.567046 -0.971266 0.788922

H -3.572624 -0.652330 -0.633540

C -3.291335 1.457822 1.479311

C -2.691468 2.441401 2.475251

H -3.241330 3.389308 2.462727

H -2.716191 2.055513 3.500692

H -1.649624 2.659178 2.226147

H -3.286593 1.877031 0.467004

H -4.336946 1.239631 1.731896

C -2.090066 0.836960 -2.307501

N -2.760953 1.692166 -1.894162

P 1.135782 -2.137549 0.917610

C 0.106995 -3.655673 1.072394

C 0.814456 -4.946481 1.475038

C 2.364411 -2.607928 -0.366224

C 3.494995 -1.597061 -0.522047

C 2.122925 -2.126818 2.469577

C 1.239628 -2.011167 3.709814

H -0.380871 -3.780448 0.100595

H -0.691225 -3.413174 1.782035

H 0.088478 -5.765891 1.530538

H 1.295430 -4.865080 2.455844

H 1.576401 -5.239038 0.746080

H 1.804927 -2.713736 -1.302401

H 2.768089 -3.597489 -0.122266

H 4.138579 -1.869032 -1.365929

H 4.122907 -1.550131 0.374455

H 3.102193 -0.593946 -0.707342

H 2.801356 -1.269186 2.405101

H 2.746471 -3.028716 2.512707

H 1.847984 -1.889034 4.612938

H 0.617983 -2.902789 3.844884

H 0.566740 -1.149746 3.633477

C -0.534628 -3.554465 -2.495188

N -0.322204 -4.659501 -2.797280

H -0.728860 -2.402985 -2.144180

Structure: TS_11/12_

Symbol X Y Z

C -0.596382 5.969730 -2.799572

C -1.962061 6.095146 -2.541455

C -2.465057 5.741910 -1.288292

C -1.609908 5.269041 -0.284337

C -0.253326 5.138021 -0.563828

C 0.262958 5.487317 -1.812794

H -0.211322 6.244701 -3.778226

H -2.636988 6.460595 -3.313458

H 0.384194 4.744319 0.221536

H 1.324993 5.377708 -2.014566

C -2.156889 4.848934 1.082511

O -1.222568 4.262718 1.898424

H -3.021215 4.178553 0.884452

O -3.793427 5.828809 -0.978113

H -4.286804 6.173387 -1.736816

Au -1.358355 4.532821 4.623027

P -0.198805 6.590901 4.957868

C 1.570541 6.378932 5.424189

C 2.366050 7.647113 5.717839

C -0.164333 7.759200 3.547346

C 0.516345 7.159806 2.321124

C -0.867273 7.630455 6.316942

C -0.831992 6.924188 7.669316

H 2.026329 5.818053 4.602403

H 1.577974 5.704636 6.288914

H 3.405301 7.396381 5.961204

H 1.954241 8.200096 6.568762

H 2.384267 8.321401 4.855174

H 0.314116 8.694503 3.864616

H -1.213266 7.981457 3.324658

H 0.394047 7.814741 1.451550

H 0.078231 6.186371 2.079314

H 1.591354 7.018265 2.484065

H -1.895244 7.878052 6.029126

H -0.312167 8.575969 6.355196

H -1.344160 7.517400 8.435187

H 0.196802 6.760659 8.008523

H -1.321453 5.945003 7.615178

P -3.716118 4.321376 4.911875

C -4.093814 3.189238 6.315626

C -5.568605 2.911656 6.591882

H -5.678091 2.270245 7.474255

H -6.129635 3.832982 6.781593

H -6.045126 2.397425 5.750796

H -3.557054 2.257841 6.104378

H -3.602100 3.623384 7.194712

C -4.664124 3.618722 3.504558

C -4.176251 2.239410 3.082400

H -4.715108 1.889839 2.195194

H -3.112256 2.263585 2.837790

H -4.324346 1.497779 3.875508

H -5.726175 3.599169 3.779785

H -4.567413 4.336610 2.683407

C -4.693164 5.835660 5.284077

C -4.426988 6.404160 6.673783

H -4.890890 7.390751 6.784190

H -4.830451 5.757789 7.460645

H -3.355342 6.515516 6.856780

H -4.417470 6.555636 4.505578

H -5.759818 5.610980 5.155938

C -2.749491 6.039099 1.751783

N -3.202864 6.953817 2.308386

P -0.034002 2.528049 4.746623

C -0.972018 0.945973 4.827529

C -0.161617 -0.337117 4.988288

C 1.221564 2.201411 3.443792

C 1.958537 3.454129 2.984161

C 0.965547 2.538361 6.291259

C 0.114081 2.785434 7.533440

H -1.561240 0.910829 3.904769

H -1.691726 1.057273 5.647169

H -0.832335 -1.203358 5.028927

H 0.431291 -0.336600 5.909499

H 0.518496 -0.490336 4.144580

H 0.682900 1.751530 2.603943

H 1.922521 1.445359 3.819487

H 2.636879 3.215107 2.156829

H 2.562867 3.884206 3.790483

H 1.245302 4.208453 2.639359

H 1.704024 3.339298 6.169164

H 1.525118 1.598435 6.373638

H 0.739162 2.860624 8.430585

H -0.604287 1.973863 7.695193

H -0.454251 3.716822 7.431636

C -1.274094 1.774715 1.150477

N -1.318126 0.663862 0.798560

H -1.219092 2.966365 1.526303

Structure: 12

Symbol X Y Z

C -0.687324 6.035087 -2.906807

C -2.054264 6.038325 -2.627831

C -2.502552 5.644518 -1.366553

C -1.590019 5.253049 -0.378585

C -0.230749 5.244962 -0.675766

C 0.228876 5.635720 -1.934131

H -0.344157 6.340429 -3.891848

H -2.771600 6.340752 -3.388455

H 0.458231 4.912429 0.093743

H 1.292567 5.620963 -2.153960

C -2.093087 4.808295 0.988968

O -1.095192 4.276158 1.802139

H -2.901863 4.073222 0.833403

O -3.825024 5.613792 -1.027511

H -4.366943 5.882531 -1.783736

Au -1.333957 4.536256 4.682664

P -0.173118 6.602518 4.973036

C 1.614478 6.423303 5.377553

C 2.383802 7.705164 5.682283

C -0.205486 7.776891 3.565977

C 0.504481 7.227153 2.332399

C -0.821152 7.617773 6.358846

C -0.767804 6.882535 7.695264

H 2.058536 5.900030 4.525295

H 1.669783 5.723972 6.220319

H 3.440547 7.478527 5.866165

H 1.997508 8.212206 6.572693

H 2.340137 8.411677 4.846634

H 0.226189 8.733461 3.886671

H -1.264753 7.948663 3.347838

H 0.332173 7.876297 1.467118

H 0.134634 6.228226 2.084772

H 1.587439 7.157971 2.486882

H -1.852613 7.872399 6.090729

H -0.263752 8.561221 6.407754

H -1.258881 7.464719 8.482980

H 0.265463 6.700845 8.010856

H -1.268754 5.909811 7.629613

P -3.701127 4.342021 4.925302

C -4.084994 3.219618 6.334306

C -5.561625 2.946932 6.605572

H -5.675492 2.314972 7.494050

H -6.122789 3.870650 6.782956

H -6.033985 2.423893 5.767746

H -3.548923 2.286112 6.131868

H -3.596513 3.658820 7.212618

C -4.653456 3.641397 3.520177

C -4.182291 2.256665 3.095692

H -4.762267 1.898597 2.238154

H -3.131057 2.265080 2.797967

H -4.299075 1.524726 3.902839

H -5.713492 3.631373 3.803977

H -4.560057 4.358529 2.697819

C -4.661640 5.868074 5.293048

C -4.396322 6.429536 6.686072

H -4.839476 7.425886 6.793352

H -4.822034 5.791198 7.467529

H -3.324232 6.516738 6.880778

H -4.373385 6.586385 4.517639

H -5.730248 5.657503 5.158137

C -2.708174 5.958042 1.688356

N -3.191539 6.862514 2.233497

P -0.045859 2.505882 4.766811

C -1.008270 0.940315 4.770564

C -0.220740 -0.354677 4.946178

C 1.248027 2.208503 3.498654

C 2.052108 3.453806 3.144857

C 0.887234 2.475685 6.352109

C -0.024914 2.648527 7.563910

H -1.541494 0.930927 3.814304

H -1.767349 1.045937 5.554461

H -0.904678 -1.211310 4.942348

H 0.332988 -0.378353 5.891412

H 0.489862 -0.503468 4.127425

H 0.719257 1.832173 2.616413

H 1.903961 1.404792 3.855775

H 2.760484 3.232612 2.338205

H 2.629876 3.819671 4.000858

H 1.387558 4.254384 2.806023

H 1.610315 3.297887 6.298398

H 1.464243 1.545187 6.420136

H 0.558052 2.707393 8.490082

H -0.723038 1.809876 7.662560

H -0.618039 3.565918 7.474024

C -1.249024 1.666376 1.221639

N -1.313631 0.514427 1.004466

H -1.102118 3.248912 1.607920

Structure: TS_12/13_

Symbol X Y Z

C 0.407055 5.600726 -2.513384

C -0.556222 6.550640 -2.177014

C -1.412291 6.349654 -1.094767

C -1.307484 5.183325 -0.303047

C -0.334480 4.240577 -0.667638

C 0.515745 4.436018 -1.754554

H 1.061480 5.773264 -3.363918

H -0.667887 7.465896 -2.752341

H -0.254357 3.341776 -0.065944

H 1.256573 3.680438 -2.003690

C -2.218626 4.880235 0.825518

O -1.827227 3.777306 1.617868

H -3.398004 4.216313 0.276124

O -2.336002 7.334956 -0.876967

H -2.800975 7.207290 -0.033791

Au -1.244762 4.637286 4.535714

P 0.017339 6.665660 4.575559

C 1.823763 6.392007 4.806602

C 2.702405 7.635846 4.901212

C -0.096527 7.794379 3.136832

C 0.516402 7.208850 1.869289

C -0.438478 7.755802 5.980611

C -0.376116 7.037736 7.325913

H 2.138091 5.757080 3.970941

H 1.924463 5.777197 5.708693

H 2.439002 8.255761 5.764528

H 2.623332 8.257245 4.003235

H 0.376584 8.748043 3.403128

H -1.165632 7.980006 2.988760

H 0.079649 6.236481 1.628276

H 1.600330 7.077922 1.961167

H -1.455398 8.105824 5.769842

H 0.207680 8.642029 5.976428

H 0.650168 6.749767 7.579443

H -0.984846 6.125878 7.312455

P -3.580371 4.460441 4.980851

C -3.859189 3.496974 6.525364

C -5.314370 3.274805 6.927867

H -5.841130 4.221428 7.087411

H -5.862439 2.711845 6.165191

H -3.343702 2.540380 6.382663

H -3.307603 4.019407 7.316496

C -4.575387 3.588413 3.708911

C -4.162285 2.137162 3.500979

H -3.093191 2.069087 3.279877

H -4.357417 1.527065 4.389864

H -5.633459 3.656827 3.989710

H -4.460484 4.160200 2.782627

C -4.541350 6.005606 5.226709

C -4.141099 6.769111 6.484313

H -4.424085 6.231133 7.395760

H -3.060283 6.931398 6.517728

H -4.358016 6.606022 4.328849

H -5.610315 5.758661 5.243034

C -2.707389 5.951328 1.619893

N -3.213631 6.836374 2.202836

P 0.066745 2.622613 4.442412

C -0.785547 1.068402 4.948039

C 0.082127 -0.172324 5.143690

C 0.818393 2.208850 2.820256

C 1.644241 3.356616 2.247765

C 1.502546 2.715950 5.585323

C 1.071023 3.016059 7.018905

H -1.548530 0.887478 4.183103

H -1.333207 1.304066 5.868101

H 0.794824 -0.044763 5.965065

H 0.648094 -0.422936 4.240567

H -0.016671 1.979919 2.149374

H 1.426108 1.301885 2.925787

H 2.505061 3.593541 2.882706

H 1.031428 4.255259 2.141624

H 2.146611 3.516720 5.208644

H 2.081574 1.786081 5.529350

H 0.462603 2.205325 7.435173

H 0.473321 3.934075 7.061393

C -4.266295 3.240613 0.059829

N -5.200993 2.585365 -0.188653

H -2.457470 3.052419 1.415655

H -5.369175 2.704385 7.862377

H -4.711957 1.702509 2.660702

H -0.546936 -1.036447 5.387002

H 1.942218 3.146203 7.670631

H -0.748307 7.681942 8.130236

H 3.754697 7.349338 5.011943

H 0.330580 7.866426 1.014043

H -4.629128 7.749591 6.512089

H 2.027947 3.096809 1.255679

Structure: 13

Symbol X Y Z

Au -0.260971 5.092327 3.117398

P -0.894055 2.938313 4.112518

C -1.806540 3.003503 5.716690

C -2.057365 1.671918 6.419178

C -1.897430 1.736746 3.140710

C -3.376434 2.114620 3.087833

C 0.593957 1.954118 4.577698

C 1.414384 2.637355 5.668919

H -2.753014 3.514365 5.509437

H -1.236497 3.673289 6.369553

H -1.121710 1.181105 6.706365

H -1.761909 0.732595 3.563103

H -1.478985 1.733756 2.130307

H -3.495451 3.147989 2.752291

H -3.862046 1.999981 4.062791

H 1.186505 1.844094 3.664092

H 0.293255 0.945108 4.886381

H 0.897018 2.612766 6.634100

H 1.600609 3.686329 5.415868

P 1.931194 4.911350 2.092408

C 3.300143 4.297586 3.175175

C 4.720447 4.405655 2.626240

H 4.835571 3.885192 1.669840

H 5.017774 5.448680 2.477399

H 3.226059 4.829565 4.128719

H 3.053992 3.252420 3.396383

C 2.589242 6.477967 1.390696

C 2.780594 7.568294 2.437805

H 1.850419 7.740648 2.988149

H 3.551938 7.302813 3.169738

H 3.526006 6.268874 0.858607

H 1.857523 6.786660 0.635409

C 2.044464 3.790306 0.633757

C 1.218889 2.516987 0.770784

H 1.580605 1.878849 1.584988

H 0.170509 2.763310 0.965019

H 1.682478 4.376500 -0.215393

H 3.100895 3.562943 0.447129

P -0.767613 6.685168 4.863141

C -0.726449 8.503235 4.542238

C -0.787461 9.423098 5.758682

C -2.407829 6.466796 5.675437

C -3.550349 6.290289 4.684840

C 0.352540 6.483854 6.312458

C 1.801079 6.836346 5.999448

H -1.566352 8.697998 3.867831

H 0.172889 8.708001 3.953425

H 0.084837 9.303831 6.409764

H -1.684005 9.253158 6.364153

H -2.585750 7.322906 6.337443

H -2.319018 5.588860 6.322669

H -3.366185 5.436492 4.026232

H -3.670182 7.166436 4.040746

H 0.276757 5.431607 6.610525

H -0.029692 7.082727 7.148862

H 1.916901 7.897632 5.754538

H 2.154845 6.262442 5.138504

H -2.618253 0.976015 5.787021

C -4.588107 8.355552 1.646405

C -3.223427 8.661068 1.648513

C -2.279491 7.652504 1.484369

C -2.671715 6.306009 1.306308

C -4.043067 6.021864 1.331353

C -4.994678 7.030435 1.500114

H -5.320111 9.149451 1.770384

H -2.875147 9.682786 1.778264

H -4.365850 4.989627 1.228860

H -6.051654 6.775779 1.514182

C -1.644538 5.255595 1.099517

O -2.172311 3.947118 0.903669

O -0.957317 7.992919 1.502977

H -0.455118 7.213037 1.838326

C -0.755672 5.578743 0.020321

N -0.030416 5.807273 -0.870818

H -2.687044 3.938752 0.076301

H 1.267265 1.927602 -0.152555

H 5.432024 3.957916 3.330347

H 3.075483 8.512825 1.967285

H 2.383691 2.143456 5.800604

H -2.640207 1.828966 7.334645

H -3.907307 1.471656 2.376248

H -4.498877 6.125844 5.209887

H -0.810938 10.469243 5.431193

H 2.453343 6.615409 6.851929

Structure: 12-SA

Symbol X Y Z

C 0.423684 4.858084 -3.592615

C -0.932527 5.181180 -3.574471

C -1.578725 5.393859 -2.354671

C -0.875091 5.269214 -1.150716

C 0.480280 4.951567 -1.186753

C 1.137736 4.745892 -2.398844

H 0.918751 4.692530 -4.545948

H -1.490489 5.265568 -4.504997

H 1.002759 4.838190 -0.243380

H 2.193801 4.491489 -2.410883

C -1.595776 5.435212 0.179628

O -0.947910 4.789219 1.234986

H -2.624082 5.060373 0.065487

O -2.900710 5.719250 -2.267504

H -3.296725 5.739364 -3.151084

Au -0.618250 4.273174 4.303992

P 0.670101 6.222067 4.704418

C 2.458267 5.873039 4.968769

C 3.344656 7.056767 5.343682

C 0.632621 7.566814 3.456819

C 1.297430 7.172125 2.141271

C 0.141996 7.064339 6.249567

C 0.179596 6.129635 7.456375

H 2.805795 5.398170 4.044143

H 2.500512 5.090777 5.736416

H 4.386686 6.734365 5.454196

H 3.038211 7.508409 6.293235

H 3.321327 7.838255 4.576648

H 1.099196 8.461974 3.887686

H -0.424362 7.800147 3.285387

H 1.191201 7.976849 1.406899

H 0.821506 6.278088 1.729758

H 2.367115 6.971850 2.270012

H -0.882385 7.408537 6.064602

H 0.757435 7.956553 6.419856

H -0.247841 6.615450 8.340627

H 1.204933 5.832713 7.703483

H -0.393221 5.215760 7.259238

P -2.933430 4.171474 4.877780

C -3.464624 5.477110 6.064322

C -4.923570 5.434371 6.509536

H -5.145207 6.273548 7.179646

H -5.607349 5.502331 5.656716

H -5.153914 4.510684 7.050899

H -2.791522 5.410700 6.927117

H -3.233397 6.431313 5.575767

C -3.407574 2.594344 5.678375

C -2.574162 2.300116 6.921433

H -2.797447 1.301736 7.314773

H -1.503924 2.342053 6.690804

H -2.769056 3.023365 7.722012

H -4.479003 2.612825 5.913133

H -3.269346 1.827653 4.909287

C -4.082743 4.306354 3.465182

C -4.029623 5.685446 2.815458

H -4.579661 5.688142 1.867762

H -4.469840 6.460226 3.453892

H -2.995667 5.975453 2.605731

H -3.759611 3.523426 2.768635

H -5.096861 4.057822 3.802271

C -1.714128 6.876876 0.495992

N -1.799391 8.008964 0.741006

P 0.421869 2.212490 3.693211

C -0.705326 0.766764 3.732064

C -0.193076 -0.530513 3.114762

C 1.233282 2.140461 2.050559

C 2.203347 3.293200 1.817346

C 1.792136 1.766498 4.839713

C 1.397713 1.868762 6.310409

H -1.614167 1.107676 3.229134

H -0.970838 0.623947 4.787210

H -0.925950 -1.331377 3.266821

H 0.754456 -0.859589 3.558043

H -0.051481 -0.424933 2.034861

H 0.432177 2.158479 1.306168

H 1.742117 1.173260 1.954977

H 2.623617 3.239190 0.806565

H 3.042098 3.273532 2.522317

H 1.689189 4.251651 1.925423

H 2.612669 2.460554 4.624770

H 2.151457 0.758068 4.596651

H 2.254671 1.657142 6.960324

H 0.605580 1.157052 6.566865

H 1.031938 2.875322 6.545707

C -2.143466 1.638307 0.659708

C -1.295781 1.705588 -0.492251

C -1.122252 0.629888 -1.340956

C -1.785660 -0.593214 -1.118015

C -2.633188 -0.685518 -0.029999

C -2.837615 0.389337 0.863055

H -0.781297 2.641568 -0.689747

H -0.460591 0.735311 -2.199314

H -1.642483 -1.434659 -1.789863

H -3.170709 -1.614732 0.160738

C -3.764739 0.142508 1.950629

O -4.103367 0.897996 2.860146

H -4.204062 -0.881706 1.919867

O -2.258211 2.630542 1.468483

H -1.417610 3.879796 1.335167

Structure: TS_12/13-SA_

Symbol X Y Z

C 0.369817 5.620326 -2.592023

C -0.658700 6.514181 -2.299074

C -1.496857 6.303822 -1.203902

C -1.311101 5.185077 -0.361830

C -0.271881 4.299766 -0.680328

C 0.562558 4.504577 -1.777376

H 1.009141 5.796654 -3.453210

H -0.837824 7.388640 -2.919053

H -0.138663 3.432458 -0.043153

H 1.355460 3.792973 -1.993640

C -2.230742 4.864736 0.761870

O -1.769426 3.796387 1.586377

H -3.412913 4.247432 0.254050

O -2.503505 7.215134 -1.038240

H -2.922879 7.125518 -0.166349

Au -1.257024 4.623006 4.503402

P 0.033129 6.631749 4.539049

C 1.828347 6.337130 4.827774

C 2.723457 7.569331 4.920041

C -0.019906 7.727821 3.072089

C 0.593790 7.087254 1.831963

C -0.446335 7.760202 5.905192

C -0.423452 7.073607 7.268106

H 2.156109 5.679182 4.015239

H 1.894649 5.741116 5.745524

H 2.445282 8.212292 5.761644

H 2.679564 8.171572 4.006710

H 0.479493 8.672492 3.321744

H -1.079585 7.944020 2.900578

H 0.130715 6.121608 1.614206

H 1.671693 6.926096 1.945189

H -1.454987 8.112515 5.660873

H 0.207535 8.640775 5.896026

H 0.593989 6.784977 7.554522

H -1.038241 6.165786 7.259939

P -3.604123 4.469166 4.891750

C -3.923995 3.513023 6.432730

C -5.389456 3.306518 6.804949

H -5.906706 4.258591 6.963342

H -5.929382 2.757933 6.026256

H -3.417795 2.550309 6.298730

H -3.382546 4.028658 7.235267

C -4.564303 3.594733 3.598966

C -4.138483 2.146322 3.394815

H -3.057109 2.078516 3.237438

H -4.380849 1.524869 4.264552

H -5.628954 3.652279 3.856223

H -4.440356 4.162715 2.672062

C -4.559691 6.021582 5.109321

C -4.172228 6.796687 6.363669

H -4.460923 6.265169 7.277233

H -3.091986 6.961874 6.403999

H -4.362516 6.612277 4.207960

H -5.629784 5.778814 5.113817

C -2.676655 5.966458 1.553857

N -3.148294 6.876534 2.124643

P 0.029489 2.588043 4.485738

C -0.851056 1.059547 5.020829

C -0.005351 -0.190616 5.249668

C 0.793889 2.124817 2.882365

C 1.660790 3.239666 2.305084

C 1.453193 2.686836 5.643676

C 1.007225 3.024613 7.064449

H -1.614461 0.873932 4.257592

H -1.398519 1.325436 5.932792

H 0.705709 -0.056123 6.071394

H 0.560401 -0.471980 4.355457

H -0.036176 1.908004 2.201131

H 1.374054 1.202990 3.011473

H 2.521667 3.458190 2.946450

H 1.077062 4.155379 2.182243

H 2.113193 3.470194 5.258336

H 2.019869 1.748072 5.616045

H 0.386514 2.229360 7.492196

H 0.417146 3.948463 7.077775

H -2.430391 3.088943 1.495867

H -5.469894 2.728288 7.732878

H -4.650497 1.737791 2.519359

H -0.650241 -1.037998 5.509885

H 1.871600 3.162565 7.723634

H -0.811553 7.738880 8.047370

H 3.767672 7.269752 5.067103

H 0.440935 7.722945 0.953753

H -4.663012 7.776146 6.379850

H 2.047894 2.955152 1.320869

C -4.481967 2.987917 -1.071072

C -3.741700 3.486422 -2.171935

C -3.971367 3.032377 -3.460668

C -4.949491 2.061280 -3.727728

C -5.684381 1.562800 -2.666166

C -5.476432 1.997014 -1.340626

H -2.976828 4.235227 -1.992951

H -3.378633 3.442472 -4.275606

H -5.125045 1.712257 -4.741085

H -6.451310 0.809355 -2.842408

C -6.323577 1.377995 -0.327072

O -6.325254 1.560594 0.884360

H -7.045948 0.648523 -0.759685

O -4.273576 3.415699 0.153475

Structure: 14

Symbol X Y Z

C 3.295265 0.038063 1.421695

C 2.407294 0.068118 0.349893

C 2.549593 1.037731 -0.658603

C 3.596171 1.972793 -0.571028

C 4.479507 1.935249 0.504389

C 4.332595 0.969671 1.502694

H 3.177238 -0.715003 2.196277

H 1.599171 -0.654316 0.282688

H 3.706593 2.721876 -1.349605

H 5.285268 2.662072 0.563536

H 5.023956 0.943318 2.340739

C 1.641201 1.072231 -1.759098

C 0.868534 1.101165 -2.693746

H 0.188275 1.126723 -3.517535

Structure: TS_6/10_

Symbol X Y Z

C -3.982792 4.666769 -2.586127

C -4.505734 4.666212 -1.292828

C -3.729449 4.204265 -0.228258

C -2.425546 3.736849 -0.441820

C -1.927015 3.735472 -1.744176

C -2.687900 4.199680 -2.816537

H -4.593161 5.029592 -3.409387

H -5.517282 5.026088 -1.109174

H -0.919693 3.354301 -1.888239

H -2.277670 4.193753 -3.822922

C -1.565418 3.205099 0.684665

O -0.523873 2.547297 0.403067

H -2.164236 2.906362 1.563443

O -4.205413 4.195413 1.055951

H -5.114739 4.528470 1.061808

Au -0.365752 4.780743 4.360565

P 1.062942 6.607981 4.980247

C 2.774553 6.067277 5.393950

C 3.719859 7.126646 5.951915

C 1.309938 8.043072 3.859627

C 2.101913 7.702004 2.600581

C 0.468985 7.402612 6.528517

C 0.347667 6.404354 7.677408

H 3.176809 5.631192 4.472345

H 2.665370 5.231775 6.095651

H 4.708102 6.693388 6.146051

H 3.351622 7.541886 6.896195

H 3.856409 7.956534 5.250517

H 1.793023 8.847402 4.429874

H 0.308724 8.393222 3.586887

H 2.192908 8.588485 1.962432

H 1.586084 6.927760 2.027105

H 3.115689 7.359089 2.835620

H -0.510572 7.831400 6.284705

H 1.126581 8.238247 6.799176

H -0.140441 6.861930 8.545251

H 1.330909 6.044898 8.000388

H -0.242346 5.531712 7.374818

P -2.699600 5.061113 4.672260

C -3.109170 5.541811 6.403225

C -4.586770 5.747935 6.723012

H -4.715274 6.018246 7.777782

H -5.021359 6.553057 6.121278

H -5.171970 4.839984 6.542155

H -2.676464 4.765813 7.046071

H -2.536573 6.453040 6.610061

C -3.764103 3.600930 4.347995

C -3.476916 2.440540 5.294224

H -4.012422 1.538227 4.978549

H -2.406931 2.210544 5.311899

H -3.783560 2.667669 6.321533

H -4.818956 3.900607 4.398970

H -3.563868 3.320529 3.308522

C -3.471194 6.353843 3.628605

C -2.793343 7.710927 3.771161

H -3.280681 8.453814 3.129393

H -2.830714 8.085357 4.801250

H -1.747553 7.632319 3.462054

H -3.375264 5.988202 2.601245

H -4.541809 6.406668 3.864354

C -1.096873 4.945234 1.440223

N -0.650382 6.011124 1.650731

P 0.805071 2.729724 4.096858

C -0.097240 1.347306 3.299861

C 0.551119 -0.031788 3.380261

C 2.387589 2.847341 3.171360

C 2.243799 3.648685 1.879955

C 1.349063 2.090516 5.733429

C 0.185498 1.833752 6.686224

H -0.222761 1.663963 2.251650

H -1.098725 1.331208 3.743843

H -0.047844 -0.757682 2.817783

H 0.627747 -0.395843 4.411292

H 1.556408 -0.037638 2.945081

H 2.760011 1.834330 2.974314

H 3.106604 3.323363 3.850071

H 3.196128 3.675928 1.336656

H 1.942020 4.676874 2.100250

H 1.475751 3.223237 1.224637

H 2.020197 2.850988 6.150501

H 1.946610 1.182319 5.584794

H 0.548240 1.512308 7.669334

H -0.479208 1.050578 6.305126

H -0.411936 2.742515 6.821501

Structure: TS_10/13_

Symbol X Y Z

C -1.304171 4.638406 -3.235472

C -2.291757 5.518908 -2.781515

C -2.569199 5.631265 -1.421666

C -1.855749 4.873854 -0.467582

C -0.879130 3.988075 -0.949001

C -0.600053 3.868583 -2.311345

H -1.100588 4.558348 -4.300189

H -2.858697 6.119049 -3.492422

H -0.341240 3.399106 -0.213910

H 0.168174 3.175480 -2.646349

C -2.124364 4.955041 0.970309

O -1.285371 4.055695 1.819590

H -2.488508 3.951316 1.606917

O -3.548726 6.464215 -0.950812

H -3.948873 6.940700 -1.692710

Au -0.512698 4.747412 4.301900

P 1.019922 6.502866 3.852919

C 2.774962 5.944151 3.806637

C 3.830714 7.008741 3.524776

C 0.775356 7.389395 2.270100

C 0.943624 6.467351 1.066056

C 1.027342 7.859764 5.090335

C 1.323109 7.363415 6.503098

H 2.810714 5.150413 3.051898

H 2.963947 5.453599 4.768928

H 4.830724 6.559492 3.503778

H 3.837314 7.788487 4.293926

H 3.666817 7.492949 2.556367

H 1.457043 8.248427 2.224980

H -0.249885 7.774139 2.306733

H 0.628823 6.970770 0.145853

H 0.332570 5.566670 1.180955

H 1.986796 6.155191 0.936775

H 0.032059 8.316157 5.036954

H 1.746891 8.629550 4.784826

H 1.204760 8.170505 7.234877

H 2.348202 6.985721 6.588254

H 0.644198 6.549025 6.782144

P -2.713199 5.004286 5.163382

C -2.912585 4.153262 6.786612

C -4.291004 4.232968 7.436023

H -4.293725 3.702084 8.395441

H -4.588824 5.268498 7.631436

H -5.061226 3.776581 6.805144

H -2.623765 3.109898 6.617850

H -2.143351 4.573251 7.446225

C -4.033084 4.286031 4.108284

C -3.829385 2.801970 3.820664

H -4.591589 2.436746 3.123024

H -2.845074 2.631374 3.372379

H -3.899772 2.199440 4.733690

H -5.004366 4.468419 4.584971

H -4.021100 4.866522 3.179337

C -3.343017 6.701703 5.464185

C -2.572552 7.435625 6.556334

H -2.882446 8.485315 6.610736

H -2.736461 6.988980 7.543718

H -1.497119 7.412751 6.356681

H -3.243968 7.213592 4.500168

H -4.413287 6.652752 5.702916

C -2.407072 6.193531 1.590314

N -2.658889 7.172937 2.187091

P 0.431178 2.540204 4.385829

C -0.563138 1.285327 5.300321

C 0.048181 -0.100162 5.492004

C 0.739799 1.758415 2.754446

C 1.673428 2.586214 1.875847

C 2.067587 2.483490 5.222763

C 2.005150 3.060103 6.635027

H -1.515852 1.219215 4.762395

H -0.796652 1.737741 6.271354

H -0.651391 -0.751936 6.028817

H 0.972103 -0.058667 6.078578

H 0.276902 -0.581880 4.535831

H -0.241447 1.680815 2.273245

H 1.132870 0.745139 2.903057

H 1.776367 2.123630 0.887629

H 2.677597 2.666833 2.307409

H 1.263183 3.589591 1.736956

H 2.748346 3.077681 4.604509

H 2.452187 1.455893 5.227000

H 3.003392 3.104891 7.085223

H 1.371598 2.452803 7.291421

H 1.590537 4.074883 6.619328

Structure: 15

Symbol X Y Z

C -3.267837 -2.238447 -4.444793

C -3.682442 -1.018925 -3.902920

C -2.841922 -0.265215 -3.091616

C -1.531443 -0.716628 -2.765709

C -1.139151 -1.950452 -3.336973

C -1.980074 -2.696430 -4.157004

H -3.937850 -2.809899 -5.081933

H -4.672258 -0.619899 -4.111916

H -0.145055 -2.319981 -3.104783

H -1.627267 -3.639991 -4.568434

C -0.616601 -0.002950 -1.925366

O 0.605758 -0.604667 -1.610359

O -3.371531 0.898468 -2.593738

H -2.666336 1.491648 -2.278560

Au 0.469144 -0.161421 1.392128

P 1.824808 1.786086 1.640633

C 3.583743 1.391707 2.024771

C 4.532477 2.569923 2.222750

C 1.907257 2.961168 0.238280

C 2.535449 2.354719 -1.011197

C 1.314069 2.865368 3.035450

C 1.178963 2.102098 4.349985

H 3.930259 0.749005 1.208154

H 3.561577 0.756806 2.918591

H 5.546973 2.210246 2.431331

H 4.229306 3.200920 3.064788

H 4.584375 3.201585 1.329833

H 2.446518 3.859246 0.565707

H 0.869758 3.243353 0.027527

H 2.514593 3.074221 -1.836484

H 1.978324 1.470819 -1.330293

H 3.580698 2.065923 -0.849404

H 0.355178 3.304195 2.737249

H 2.024153 3.695769 3.132104

H 0.779825 2.749179 5.139058

H 2.146594 1.718564 4.692465

H 0.502206 1.247038 4.236853

P -1.896844 -0.266843 1.653317

C -2.264984 -1.174249 3.215893

C -3.737353 -1.344652 3.578922

H -3.835977 -1.851291 4.546310

H -4.251816 -0.380971 3.656815

H -4.268470 -1.948304 2.835985

H -1.774231 -2.150193 3.122085

H -1.725536 -0.643412 4.009964

C -2.871857 -1.133965 0.367541

C -2.295766 -2.482211 -0.036974

H -2.889808 -2.923664 -0.843804

H -1.276062 -2.356626 -0.410992

H -2.281029 -3.186226 0.803548

H -3.903715 -1.227678 0.728803

H -2.896852 -0.461382 -0.493360

C -2.818141 1.313666 1.800802

C -2.476380 2.098026 3.061675

H -2.933447 3.093496 3.034252

H -2.830184 1.593639 3.967827

H -1.394793 2.228358 3.155653

H -2.541085 1.881629 0.904424

H -3.894182 1.104620 1.745215

C -0.829785 1.261613 -1.413047

N -1.043650 2.352351 -0.994214

P 1.686842 -2.237520 1.308976

C 0.703006 -3.767167 1.608064

C 1.474602 -5.062198 1.848131

C 2.590773 -2.613986 -0.242967

C 3.577180 -1.518333 -0.636038

C 2.982908 -2.296336 2.611162

C 2.398814 -2.046456 3.999840

H 0.046986 -3.862733 0.736416

H 0.043882 -3.550011 2.456604

H 0.777551 -5.902406 1.948815

H 2.068457 -5.017821 2.767114

H 2.151491 -5.296272 1.019818

H 1.817388 -2.713305 -1.011875

H 3.099956 -3.580118 -0.140678

H 4.018243 -1.733514 -1.615938

H 4.400641 -1.435129 0.082016

H 3.071873 -0.550559 -0.695701

H 3.709732 -1.516517 2.361762

H 3.513120 -3.255683 2.567576

H 3.189604 -2.007390 4.757498

H 1.697994 -2.837226 4.290573

H 1.853560 -1.095654 4.025313

H 1.211328 -0.428040 -2.368707

C -1.168721 1.079246 -5.343985

C -2.305066 1.831562 -5.617893

C -2.413438 3.143752 -5.153224

C -1.373888 3.707096 -4.407987

C -0.231326 2.966933 -4.128233

C -0.118645 1.642838 -4.592838

H -1.093131 0.049593 -5.678891

H -3.120766 1.380419 -6.175837

H -3.310138 3.722009 -5.360499

H -1.460196 4.722712 -4.030843

H 0.570272 3.392983 -3.534373

C 1.055159 0.884265 -4.328448

C 2.058076 0.228070 -4.108423

H 2.988943 -0.293388 -4.043536

Structure: TS_15/16-bra_

Symbol X Y Z

Au 0.227891 4.815659 3.736936

P -0.599816 2.701447 4.557951

C -1.575919 2.861135 6.112774

C -2.014311 1.570064 6.797040

C -1.653236 1.643062 3.488728

C -2.964796 2.322944 3.103912

C 0.775276 1.572490 5.017534

C 1.739696 2.215503 6.011355

H -2.445304 3.475150 5.852261

H -0.967405 3.473551 6.789068

H -1.157857 0.959327 7.101857

H -1.828017 0.686592 3.997920

H -1.059729 1.423058 2.594609

H -2.769386 3.290125 2.631148

H -3.604383 2.488191 3.978253

H 1.290615 1.330551 4.080342

H 0.370860 0.631284 5.410012

H 1.260561 2.382127 6.982524

H 2.089985 3.186256 5.642313

P 2.033526 4.713739 2.195787

C 3.336516 3.580069 2.841904

C 4.547196 3.337008 1.945938

H 4.255885 2.919792 0.976044

H 5.107701 4.259064 1.761142

H 3.646711 3.993701 3.809123

H 2.829450 2.636799 3.071402

C 2.959758 6.224110 1.723061

C 3.487105 7.011568 2.914955

H 2.660094 7.324627 3.558081

H 4.185432 6.426423 3.525266

H 3.770580 5.933241 1.043474

H 2.253383 6.836666 1.153537

C 1.575972 4.031751 0.557020

C 0.941979 2.649656 0.640071

H 1.623369 1.909862 1.076564

H 0.038149 2.690246 1.253329

H 0.864567 4.743756 0.124390

H 2.466356 4.027948 -0.084172

P -0.560311 6.660698 5.018987

C -0.577556 8.295842 4.186274

C -0.871029 9.511489 5.059048

C -2.247530 6.489931 5.727241

C -3.307240 6.184586 4.675859

C 0.430601 6.880734 6.552944

C 1.915077 7.120339 6.304258

H -1.320425 8.205369 3.386541

H 0.384488 8.396688 3.672987

H -0.103445 9.659616 5.826636

H -1.841569 9.432552 5.560416

H -2.494070 7.399620 6.289080

H -2.179816 5.677076 6.459471

H -3.017970 5.326671 4.060791

H -3.457340 7.025791 3.993721

H 0.288154 5.958795 7.130560

H -0.008500 7.696111 7.141388

H 2.084677 8.047085 5.745679

H 2.350962 6.297363 5.728325

H -2.648204 0.960691 6.144437

C -5.847430 7.591801 1.465932

C -4.812151 8.514155 1.337869

C -3.481988 8.101534 1.247273

C -3.146191 6.725121 1.313727

C -4.217923 5.814781 1.425169

C -5.541599 6.227442 1.500324

H -6.877072 7.934658 1.528082

H -5.012921 9.581441 1.290759

H -3.973858 4.758549 1.458878

H -6.333275 5.486923 1.588263

C -1.798897 6.194641 1.248174

O -1.566663 4.900422 1.546685

O -2.559449 9.108002 1.156242

H -1.702240 8.773033 0.839860

C -0.677538 7.003919 1.034432

N 0.236335 7.716602 0.812279

H -1.601168 4.382041 0.550455

C -3.394021 7.417766 -1.743144

C -3.579701 8.726172 -2.172483

C -2.488943 9.508090 -2.561623

C -1.200288 8.965606 -2.525842

C -0.996702 7.658176 -2.101381

C -2.093742 6.861665 -1.698635

H -4.238171 6.815245 -1.422841

H -4.583019 9.144290 -2.187236

H -2.640700 10.533693 -2.888214

H -0.347127 9.569747 -2.824897

H 0.002773 7.238311 -2.062715

C -1.897383 5.546992 -1.227370

C -1.756748 4.337209 -0.923080

H -1.747028 3.370988 -1.409100

H -0.897571 10.414707 4.438993

H 4.012266 7.912669 2.578650

H 5.233182 2.624511 2.419096

H 2.614677 1.577293 6.177432

H 2.458422 7.200381 7.252788

H -2.594201 1.796527 7.699398

H -3.526363 1.700318 2.398151

H -4.271524 5.962287 5.146905

H 0.658465 2.296493 -0.357153

Structure: TS_15/16-lin_

Symbol X Y Z

C -3.473022 -2.419935 -3.115737

C -3.985940 -1.123635 -3.071813

C -3.146985 -0.022124 -2.901863

C -1.751201 -0.195997 -2.738276

C -1.263497 -1.515844 -2.790773

C -2.096770 -2.613235 -2.979468

H -4.143572 -3.263525 -3.255665

H -5.051631 -0.938664 -3.178469

H -0.193705 -1.649671 -2.682483

H -1.674149 -3.614512 -3.015951

C -0.783819 0.897429 -2.625258

O 0.489522 0.546729 -2.221878

O -3.764744 1.199976 -2.908248

H -3.132962 1.920346 -2.739024

Au 0.449489 -0.267947 0.491289

P 1.583729 1.711156 1.268233

C 3.403858 1.522203 1.480280

C 4.149649 2.706983 2.087139

C 1.394319 3.274356 0.319790

C 2.099569 3.254900 -1.033644

C 1.013830 2.181484 2.952616

C 1.277287 1.085878 3.982764

H 3.800715 1.280213 0.489286

H 3.544589 0.619789 2.087466

H 5.222466 2.489804 2.149142

H 3.801036 2.932841 3.100461

H 4.031641 3.611673 1.481592

H 1.754006 4.102284 0.944283

H 0.319723 3.423431 0.169414

H 1.908571 4.191627 -1.568255

H 1.724135 2.434704 -1.650000

H 3.184720 3.145439 -0.928120

H -0.060400 2.377749 2.866494

H 1.487842 3.124354 3.252365

H 0.813775 1.334942 4.943900

H 2.349731 0.947908 4.157783

H 0.868207 0.125771 3.649014

P -1.850817 -0.414205 1.075410

C -1.979450 -0.530858 2.909875

C -3.389223 -0.645814 3.482306

H -3.351112 -0.711725 4.575950

H -4.002038 0.224777 3.225985

H -3.905842 -1.538902 3.115719

H -1.364757 -1.388947 3.207396

H -1.472145 0.354021 3.309139

C -2.831476 -1.837358 0.466416

C -2.299187 -3.180153 0.949539

H -2.802460 -4.003326 0.430402

H -1.226865 -3.263579 0.751538

H -2.453396 -3.317746 2.025841

H -3.878808 -1.697026 0.761457

H -2.794802 -1.781637 -0.625101

C -2.939048 0.997130 0.641994

C -2.508650 2.319451 1.260732

H -3.148511 3.133336 0.905534

H -2.568279 2.295215 2.355013

H -1.483736 2.563906 0.972382

H -2.931468 1.065783 -0.448549

H -3.965024 0.739359 0.932529

C -1.175651 2.177218 -2.173732

N -1.505004 3.266673 -1.875503

P 1.870976 -2.005673 -0.327410

C 1.099206 -3.647621 -0.653190

C 1.955274 -4.690953 -1.366804

C 2.790930 -1.643598 -1.873698

C 3.580800 -0.341640 -1.824832

C 3.200200 -2.390610 0.883925

C 2.652209 -2.646784 2.285684

H 0.180855 -3.449937 -1.216082

H 0.777614 -4.021175 0.326270

H 1.422153 -5.647883 -1.410857

H 2.905734 -4.868219 -0.851321

H 2.179400 -4.391924 -2.395384

H 2.050442 -1.601108 -2.679299

H 3.455174 -2.488030 -2.092342

H 4.018615 -0.131324 -2.804789

H 4.397810 -0.393920 -1.096564

H 2.932378 0.495325 -1.555255

H 3.865633 -1.520360 0.893963

H 3.791328 -3.242643 0.525731

H 3.467126 -2.808228 3.000417

H 2.006499 -3.531517 2.313577

H 2.058774 -1.792132 2.629907

H 1.035179 0.594676 -3.065571

C 3.309910 1.578676 -5.007594

C 4.636730 1.212070 -5.201531

C 4.983390 -0.116201 -5.471466

C 3.968882 -1.076358 -5.552511

C 2.637585 -0.725239 -5.363479

C 2.273743 0.614992 -5.080922

H 3.051542 2.611777 -4.791587

H 5.411891 1.972770 -5.138611

H 6.022649 -0.396909 -5.619383

H 4.219050 -2.113559 -5.765418

H 1.856530 -1.478585 -5.425388

C 0.925176 0.970965 -4.861880

C -0.270574 1.270861 -4.667441

H -1.204395 1.607715 -5.087253

Structure: 16-bra

Symbol X Y Z

C -2.109883 7.334167 -2.674221

C -2.941660 8.020659 -1.798380

C -3.178649 7.536549 -0.504043

C -2.549375 6.358184 -0.069213

C -1.725016 5.681590 -0.971893

C -1.499643 6.145407 -2.263137

H -1.942401 7.725245 -3.674977

H -3.433819 8.943002 -2.095594

H -1.238960 4.785306 -0.599080

H -0.848035 5.595219 -2.937397

C -2.663524 5.680225 1.335564

O -1.574154 4.971540 1.654002

H -4.715927 2.849710 1.173284

O -4.011149 8.310887 0.251364

H -4.302784 7.840882 1.049374

Au -0.302542 4.985241 3.948023

P 1.057040 6.928251 3.695754

C 2.737860 6.558326 3.036423

C 3.681522 7.740720 2.838989

C 0.417803 8.245149 2.592165

C 0.317192 7.775653 1.144201

C 1.413431 7.847490 5.246180

C 2.028642 6.963455 6.327192

H 2.574672 6.029706 2.091344

H 3.175390 5.823049 3.722134

H 4.634838 7.401153 2.416750

H 3.904046 8.247334 3.784115

H 3.262077 8.481860 2.150460

H 1.049553 9.137587 2.690409

H -0.575789 8.497406 2.977844

H -0.220705 8.508813 0.533640

H -0.226430 6.827619 1.087369

H 1.308066 7.631338 0.697398

H 0.454389 8.262406 5.576629

H 2.063587 8.701583 5.019702

H 2.125189 7.508529 7.273070

H 3.027931 6.618044 6.039275

H 1.409561 6.076681 6.506020

P -2.066623 4.764167 5.522879

C -1.586825 3.636942 6.899672

C -2.640233 3.394220 7.976680

H -2.259664 2.702285 8.737402

H -2.920771 4.322145 8.485970

H -3.552442 2.955767 7.558147

H -1.285501 2.694449 6.428264

H -0.671590 4.058039 7.333173

C -3.636271 4.052661 4.883539

C -3.517245 2.593390 4.467030

H -4.448886 2.250765 4.004382

H -2.717891 2.471674 3.733172

H -3.302956 1.939438 5.319698

H -4.419700 4.185240 5.640365

H -3.910691 4.660943 4.015788

C -2.681510 6.266580 6.388227

C -1.651812 6.890651 7.322669

H -2.006316 7.855966 7.701311

H -1.443473 6.250562 8.187209

H -0.706416 7.060819 6.802040

H -2.958263 6.966521 5.592620

H -3.597966 6.009816 6.935045

C -2.866931 6.746562 2.381080

N -2.933783 7.522940 3.247186

P 0.687820 2.977437 3.060165

C -0.024182 1.373985 3.637132

C 0.767019 0.103880 3.334670

C 0.629972 2.811106 1.233529

C 1.329510 3.955343 0.508164

C 2.472792 2.819177 3.473803

C 2.711841 2.890822 4.980112

H -1.018954 1.322105 3.179818

H -0.193611 1.472937 4.715312

H 0.199227 -0.780086 3.648750

H 1.722847 0.085647 3.868692

H 0.977160 -0.004095 2.265287

H -0.436371 2.827241 0.983143

H 1.047459 1.839303 0.941268

H 1.221248 3.842320 -0.576748

H 2.402755 3.988324 0.728557

H 0.871621 4.905312 0.793502

H 2.981996 3.648444 2.972434

H 2.875318 1.891579 3.048218

H 3.783141 2.866650 5.210695

H 2.239429 2.051719 5.503866

H 2.292375 3.815631 5.392812

C -7.012760 6.223576 -0.498309

C -5.824955 5.538751 -0.251042

C -5.262190 5.521204 1.033856

C -5.919450 6.208687 2.066060

C -7.109469 6.894568 1.818048

C -7.657201 6.907008 0.534934

H -7.431198 6.232751 -1.501702

H -5.307357 5.024478 -1.056265

H -5.500228 6.201842 3.067946

H -7.606313 7.420310 2.629570

H -8.579662 7.447967 0.340235

C -3.972608 4.830947 1.264565

C -3.854605 3.503373 1.287000

H -2.867990 3.071039 1.428493

Structure: 16-lin

Symbol X Y Z

C -2.914244 7.474472 -2.899744

C -3.845043 7.891772 -1.951796

C -3.720043 7.495204 -0.617087

C -2.657930 6.664491 -0.223103

C -1.721760 6.278993 -1.182376

C -1.839144 6.670445 -2.514403

H -3.024094 7.789642 -3.934552

H -4.677310 8.535384 -2.223848

H -0.904866 5.653485 -0.835294

H -1.099838 6.351988 -3.245083

C -2.477008 6.064430 1.204580

O -1.270826 5.515011 1.387556

O -4.695413 7.939405 0.239846

H -4.345119 8.006203 1.145170

Au -0.475516 4.850819 3.718651

P 1.109714 6.654061 3.927477

C 2.781994 6.299614 3.236191

C 3.826826 7.405454 3.352839

C 0.647354 8.250096 3.144704

C 0.609135 8.157495 1.620082

C 1.491663 7.141663 5.661254

C 2.058983 5.990409 6.486471

H 2.621701 6.026130 2.188335

H 3.129142 5.388960 3.737392

H 4.767805 7.090848 2.885801

H 4.045672 7.651019 4.397491

H 3.502137 8.323695 2.852446

H 1.336901 9.032768 3.487758

H -0.348602 8.500616 3.525127

H 0.185684 9.074391 1.194425

H -0.007766 7.307682 1.303502

H 1.614255 8.030710 1.200414

H 0.552320 7.493146 6.098065

H 2.179595 7.996341 5.654523

H 2.179618 6.284078 7.535499

H 3.039873 5.671756 6.116775

H 1.392779 5.121612 6.451033

P -2.274045 4.795451 5.270573

C -1.594973 4.179459 6.871410

C -2.572944 4.040557 8.033893

H -2.056517 3.655173 8.921070

H -3.018732 5.002999 8.306299

H -3.387375 3.346752 7.800039

H -1.118200 3.218486 6.643233

H -0.778124 4.861567 7.133233

C -3.746905 3.735163 4.976167

C -3.430637 2.256062 4.819573

H -4.333330 1.694650 4.556400

H -2.704079 2.104197 4.019911

H -3.016924 1.828218 5.740300

H -4.462034 3.902204 5.791743

H -4.205618 4.121776 4.060905

C -3.131081 6.358244 5.726431

C -2.203548 7.478340 6.171687

H -2.778254 8.376301 6.424249

H -1.617386 7.196372 7.054229

H -1.516275 7.743189 5.364726

H -3.685474 6.669419 4.835091

H -3.870293 6.125186 6.503185

C -2.687050 7.201464 2.177878

N -2.821732 8.096128 2.912946

P 0.461442 2.911761 2.651607

C -0.388326 1.298811 2.930950

C 0.266838 0.040085 2.369316

C 0.574824 3.021174 0.821337

C 1.382187 4.226103 0.348436

C 2.192548 2.551514 3.159008

C 2.314915 2.321074 4.662479

H -1.393825 1.422588 2.514413

H -0.524681 1.209987 4.014941

H -0.361436 -0.834631 2.575634

H 1.246045 -0.146226 2.822958

H 0.402979 0.097412 1.284552

H -0.454367 3.114076 0.458797

H 0.994334 2.085331 0.431743

H 1.382036 4.275252 -0.746890

H 2.427562 4.170951 0.672964

H 0.926397 5.143232 0.730550

H 2.790193 3.417618 2.858848

H 2.570526 1.691763 2.591749

H 3.363440 2.200406 4.957889

H 1.775263 1.420715 4.977441

H 1.898767 3.169023 5.217386

C -3.658846 5.102396 1.395726

C -3.425763 3.790179 1.499388

H -2.376940 3.498097 1.481748

C -4.416976 2.721398 1.627782

C -5.730123 2.951300 2.072217

C -4.040607 1.400036 1.337268

C -6.627535 1.898468 2.224426

H -6.043867 3.962074 2.320384

C -4.936966 0.343967 1.486484

H -3.031741 1.205658 0.979756

C -6.235548 0.587901 1.936195

H -7.635965 2.098618 2.578438

H -4.620991 -0.670079 1.253849

H -6.936208 -0.233425 2.062298

H -4.664739 5.519169 1.360770

Structure: TS_16-bra/17_

Symbol X Y Z

C -1.428838 5.469174 -2.159533

C -1.935808 6.603727 -1.538547

C -2.479912 6.536439 -0.248548

C -2.488096 5.313922 0.452052

C -1.964653 4.189222 -0.198748

C -1.444574 4.244348 -1.487848

H -1.022916 5.542595 -3.165715

H -1.932601 7.568374 -2.039133

H -1.981924 3.248980 0.343814

H -1.055465 3.346168 -1.960361

C -3.065291 5.014506 1.842415

O -2.432444 4.180451 2.548534

H -6.256664 3.754277 2.439924

O -2.956521 7.704280 0.244478

H -3.222087 7.562436 1.179029

Au -0.257599 5.239157 4.815370

P 1.037194 7.146153 4.206323

C 2.707165 6.714437 3.559113

C 3.618844 7.874717 3.171759

C 0.318079 8.259435 2.943484

C 0.166474 7.583003 1.586319

C 1.400548 8.282924 5.601556

C 2.074135 7.571628 6.772235

H 2.533149 6.055097 2.702045

H 3.177506 6.089587 4.327889

H 4.569826 7.496635 2.778433

H 3.850284 8.515222 4.029390

H 3.167420 8.501294 2.395372

H 0.932298 9.166337 2.875111

H -0.666745 8.545046 3.328832

H -0.341734 8.247151 0.881385

H -0.439632 6.676101 1.669240

H 1.134999 7.308481 1.153269

H 0.435031 8.705227 5.902255

H 2.013717 9.118596 5.242395

H 2.174918 8.242177 7.632998

H 3.077341 7.221983 6.504211

H 1.491130 6.698380 7.088402

P -2.050856 5.258074 6.380879

C -1.577368 4.307574 7.887594

C -2.649475 4.169832 8.964661

H -2.262170 3.607438 9.822523

H -2.982778 5.145928 9.332967

H -3.529211 3.636331 8.589626

H -1.246791 3.322729 7.538685

H -0.680717 4.797691 8.286501

C -3.618139 4.489380 5.818064

C -3.483009 3.009289 5.485196

H -4.467257 2.579913 5.267141

H -2.858394 2.894293 4.596471

H -3.049339 2.433082 6.310553

H -4.375227 4.659798 6.594159

H -3.924050 5.046257 4.925844

C -2.635486 6.870008 7.041261

C -1.605298 7.577752 7.913998

H -1.934327 8.595277 8.153406

H -1.440272 7.051075 8.860580

H -0.641283 7.649193 7.402933

H -2.876793 7.464363 6.153902

H -3.569118 6.707186 7.594920

C -2.980251 6.680059 2.799485

N -2.870060 7.363782 3.745273

P 0.594045 3.130071 4.059006

C -0.134260 1.630339 4.844431

C 0.604015 0.309466 4.643063

C 0.459296 2.775992 2.267843

C 1.177076 3.800380 1.396330

C 2.388460 2.995339 4.436253

C 2.676429 3.240093 5.915872

H -1.153716 1.568847 4.448872

H -0.238401 1.853762 5.912250

H 0.032159 -0.514044 5.086864

H 1.589504 0.318554 5.120587

H 0.745704 0.075698 3.582645

H -0.617104 2.815746 2.068749

H 0.824753 1.761485 2.066608

H 0.981149 3.604217 0.337307

H 2.262618 3.781800 1.547172

H 0.813469 4.808365 1.611723

H 2.890588 3.752453 3.825057

H 2.766455 2.018042 4.111512

H 3.753697 3.215457 6.116157

H 2.202288 2.482457 6.550240

H 2.293358 4.219280 6.227068

C -6.309828 6.711291 -1.038372

C -5.573535 5.772473 -0.318609

C -5.393854 5.915914 1.063886

C -5.969857 7.016353 1.710990

C -6.707117 7.956979 0.990968

C -6.876260 7.808531 -0.386134

H -6.438703 6.588340 -2.111025

H -5.124134 4.923196 -0.826307

H -5.828578 7.132485 2.782017

H -7.146372 8.807646 1.506490

H -7.446307 8.543649 -0.948910

C -4.600334 4.919881 1.824606

C -5.177995 3.890421 2.450282

H -4.562547 3.166332 2.974167

Structure: 17

Symbol X Y Z

C -2.165597 2.056397 -3.797275

C -1.545486 0.826334 -3.625858

C -2.273303 -0.292023 -3.173048

C -3.658509 -0.139904 -2.919214

C -4.257552 1.118587 -3.087665

C -3.530959 2.215341 -3.527515

H -1.579135 2.902692 -4.146759

H -0.486137 0.708156 -3.825937

H -5.315701 1.210304 -2.857992

H -4.014048 3.178457 -3.665402

C -4.498671 -1.226959 -2.363364

O -5.392799 -0.974040 -1.556095

O -1.665715 -1.455402 -2.927654

H -0.662857 -1.301016 -2.675845

Au 0.727547 0.003152 0.806427

P 1.664215 2.177921 0.364633

C 3.462958 2.224059 -0.012507

C 4.102573 3.602194 -0.153271

C 0.923203 3.216566 -0.964029

C 1.324255 2.758214 -2.364431

C 1.494141 3.234519 1.861172

C 2.102456 2.580214 3.099750

H 3.579781 1.633285 -0.927629

H 3.957358 1.648134 0.776467

H 5.159369 3.502756 -0.427925

H 4.059217 4.167743 0.783818

H 3.616787 4.202587 -0.929688

H 1.200675 4.265570 -0.796352

H -0.162543 3.146383 -0.842811

H 0.744848 3.298222 -3.120956

H 1.152816 1.686377 -2.490737

H 2.384336 2.948257 -2.562101

H 0.419962 3.396109 2.008959

H 1.941547 4.217865 1.671054

H 1.939918 3.200065 3.988786

H 3.183095 2.436319 2.988463

H 1.651241 1.597460 3.279289

P -1.635328 -0.158166 0.886768

C -2.261497 -0.027881 2.614045

C -3.772620 -0.170865 2.777868

H -4.059425 -0.016367 3.824742

H -4.320766 0.558132 2.171809

H -4.114819 -1.168448 2.484574

H -1.737387 -0.788956 3.201395

H -1.911277 0.938603 2.995666

C -2.401255 -1.690133 0.243454

C -1.945974 -2.944067 0.976254

H -2.369598 -3.831859 0.498808

H -0.855551 -3.029676 0.955470

H -2.262787 -2.944667 2.025440

H -3.490120 -1.581513 0.289834

H -2.129266 -1.752291 -0.815363

C -2.544569 1.171190 0.000383

C -2.463117 2.524068 0.699172

H -2.824405 3.318463 0.036809

H -3.069420 2.549215 1.610635

H -1.432993 2.766128 0.982190

H -2.094541 1.230539 -0.995160

H -3.587040 0.865973 -0.143354

C 0.868662 -1.018351 -2.137274

N 1.982501 -0.747681 -1.875525

P 2.266772 -1.639310 1.601922

C 2.233225 -3.212432 0.657033

C 3.144271 -4.325364 1.165737

C 4.048774 -1.172033 1.623864

C 4.675291 -1.114674 0.232108

C 2.018507 -2.123333 3.360326

C 0.670678 -2.782163 3.627358

H 2.471480 -2.927803 -0.373820

H 1.188718 -3.541549 0.636210

H 3.076982 -5.199228 0.507245

H 2.865865 -4.651289 2.174003

H 4.194168 -4.014529 1.192312

H 4.594717 -1.871204 2.270870

H 4.090358 -0.191987 2.114733

H 5.645464 -0.605603 0.266525

H 4.027057 -0.596460 -0.478971

H 4.843114 -2.121903 -0.163704

H 2.117797 -1.200619 3.945774

H 2.842526 -2.782002 3.662669

H 0.536674 -2.975637 4.697649

H 0.577970 -3.737946 3.100727

H -0.148046 -2.140957 3.290331

C -4.284756 -2.627549 -2.847463

C -4.182680 -2.817394 -4.172421

H -4.150684 -1.977418 -4.861282

H -4.147436 -3.815739 -4.601271

C -4.253263 -3.750771 -1.884737

C -5.121678 -3.834332 -0.785313

C -3.310134 -4.772849 -2.076282

C -5.060533 -4.925516 0.080373

H -5.846661 -3.045094 -0.622806

C -3.255282 -5.865383 -1.212817

H -2.600248 -4.690129 -2.894693

C -4.133921 -5.948039 -0.131204

H -5.745568 -4.978302 0.923157

H -2.514653 -6.644228 -1.376691

H -4.089452 -6.796453 0.547042

Structure: TS_17/18_

Symbol X Y Z

C -2.169303 2.068689 -3.808053

C -1.571021 0.824519 -3.688527

C -2.287758 -0.301421 -3.201740

C -3.666204 -0.105918 -2.884813

C -4.241737 1.170566 -3.001863

C -3.515712 2.259007 -3.459859

H -1.584320 2.906666 -4.181724

H -0.529328 0.689027 -3.962491

H -5.286041 1.281138 -2.720396

H -3.982360 3.234960 -3.560273

C -4.511110 -1.179320 -2.328252

O -5.392901 -0.932135 -1.501204

O -1.685467 -1.447251 -2.991657

H -0.485594 -1.305998 -2.661506

Au 0.747196 0.003983 0.852057

P 1.675757 2.176376 0.377513

C 3.478404 2.251104 0.015828

C 4.088743 3.639711 -0.151786

C 0.928283 3.172392 -0.977421

C 1.319047 2.660302 -2.361803

C 1.475135 3.249481 1.857699

C 2.083749 2.622764 3.110457

H 3.622602 1.646417 -0.886203

H 3.978080 1.704284 0.822264

H 5.152756 3.556984 -0.402654

H 4.013478 4.230571 0.767287

H 3.605049 4.205037 -0.955281

H 1.211513 4.225187 -0.849509

H -0.156887 3.111007 -0.847179

H 0.750222 3.186267 -3.135415

H 1.118289 1.590063 -2.456156

H 2.382974 2.820077 -2.565993

H 0.397480 3.394981 1.994066

H 1.907362 4.237327 1.657088

H 1.901961 3.252514 3.988608

H 3.167677 2.496296 3.011535

H 1.647705 1.635222 3.301003

P -1.620959 -0.167862 0.875831

C -2.285834 -0.072832 2.590745

C -3.799718 -0.228307 2.713693

H -4.113143 -0.095401 3.755853

H -4.337255 0.508437 2.107705

H -4.127644 -1.222179 2.393034

H -1.771627 -0.842418 3.175806

H -1.951965 0.888606 2.998906

C -2.351378 -1.695835 0.188055

C -1.886344 -2.956335 0.903102

H -2.280761 -3.841700 0.397104

H -0.794134 -3.018786 0.905900

H -2.226585 -2.987132 1.944573

H -3.441693 -1.602791 0.227190

H -2.073381 -1.731258 -0.872036

C -2.515876 1.168080 -0.010740

C -2.450898 2.514061 0.702021

H -2.809477 3.311817 0.042313

H -3.068877 2.526577 1.605979

H -1.426217 2.760555 1.000635

H -2.054332 1.235852 -1.000929

H -3.554427 0.859990 -0.172636

C 0.757769 -1.154533 -2.230000

N 1.864579 -0.977037 -1.897665

P 2.282072 -1.645614 1.640145

C 2.246826 -3.236764 0.725465

C 3.177335 -4.330117 1.241885

C 4.060812 -1.171796 1.633266

C 4.651758 -1.106314 0.226416

C 2.035073 -2.097238 3.406340

C 0.690197 -2.762385 3.676206

H 2.465881 -2.974243 -0.315293

H 1.206109 -3.577359 0.730502

H 3.098946 -5.221337 0.608533

H 2.925580 -4.630729 2.264835

H 4.225597 -4.013057 1.233811

H 4.624755 -1.871348 2.263815

H 4.108273 -0.193327 2.126376

H 5.619843 -0.592803 0.236362

H 3.983813 -0.586071 -0.464890

H 4.812040 -2.110554 -0.179472

H 2.123096 -1.163000 3.974830

H 2.863011 -2.743193 3.724551

H 0.547852 -2.931471 4.749412

H 0.611505 -3.731271 3.171833

H -0.131253 -2.136935 3.316215

C -4.335660 -2.578566 -2.838300

C -4.312501 -2.758092 -4.167921

H -4.307712 -1.911348 -4.849206

H -4.312013 -3.753064 -4.606477

C -4.270859 -3.712929 -1.890468

C -5.088244 -3.798566 -0.752539

C -3.351814 -4.745203 -2.136322

C -5.001799 -4.899888 0.097978

H -5.794357 -3.001361 -0.549675

C -3.271464 -5.848080 -1.287773

H -2.680667 -4.662225 -2.986692

C -4.099777 -5.931979 -0.167166

H -5.647969 -4.953361 0.970996

H -2.550246 -6.635005 -1.494725

H -4.035473 -6.788785 0.498951

Structure: 18

Symbol X Y Z

C -1.569197 2.980933 -3.242917

C -1.164423 1.667617 -3.397251

C -1.985958 0.564567 -2.998765

C -3.277091 0.906784 -2.459778

C -3.650605 2.253179 -2.296900

C -2.823019 3.294506 -2.683557

H -0.906590 3.784720 -3.561729

H -0.195748 1.431241 -3.832009

H -4.623365 2.456377 -1.854499

H -3.137898 4.328404 -2.571285

C -4.207330 -0.118490 -1.974061

O -4.974290 0.090571 -1.025836

O -1.557071 -0.646763 -3.067590

H -0.094618 -1.070868 -2.601015

Au 1.271888 0.413278 0.886556

P 2.194367 2.423034 -0.042099

C 4.005421 2.622121 0.225922

C 4.636656 3.917549 -0.275395

C 1.956723 2.698649 -1.837152

C 2.699335 1.682877 -2.700216

C 1.465685 3.933457 0.702841

C 1.619659 3.962987 2.221523

H 4.475303 1.753010 -0.247892

H 4.166616 2.499499 1.303855

H 5.715780 3.914391 -0.082537

H 4.216130 4.795855 0.225422

H 4.495892 4.045119 -1.353866

H 2.257404 3.724764 -2.084512

H 0.876872 2.625860 -2.005688

H 2.451294 1.826418 -3.757673

H 2.428620 0.658532 -2.428176

H 3.785871 1.783437 -2.600028

H 0.407687 3.926373 0.417558

H 1.914450 4.822544 0.243180

H 1.074829 4.810096 2.652537

H 2.670748 4.059424 2.515538

H 1.230752 3.043151 2.674668

P -1.076510 0.104945 1.083065

C -1.601259 -0.250660 2.811612

C -3.094567 -0.506923 3.002139

H -3.325633 -0.635548 4.066176

H -3.703669 0.321932 2.626649

H -3.415037 -1.414755 2.481398

H -1.013462 -1.113625 3.142842

H -1.267381 0.596940 3.422116

C -1.801647 -1.266360 0.121406

C -1.222643 -2.630000 0.474084

H -1.690980 -3.399292 -0.146752

H -0.144000 -2.656357 0.296256

H -1.404591 -2.893554 1.522756

H -2.882544 -1.246242 0.292003

H -1.656600 -1.048157 -0.943048

C -2.083219 1.557559 0.594383

C -1.997906 2.707307 1.592453

H -2.433083 3.616899 1.163694

H -2.542241 2.482936 2.516055

H -0.960534 2.926745 1.866517

H -1.703839 1.867180 -0.384521

H -3.121674 1.244732 0.438395

C 0.853652 -1.493039 -2.138268

N 1.802278 -1.943558 -1.642501

P 2.875950 -1.086176 1.838485

C 2.462626 -2.866084 1.661532

C 3.470416 -3.868742 2.215035

C 4.612315 -0.941657 1.254473

C 4.770151 -1.188864 -0.242106

C 3.083278 -0.809324 3.644702

C 1.769883 -0.865075 4.418079

H 2.300567 -3.017742 0.588432

H 1.481817 -2.990416 2.135335

H 3.098550 -4.890350 2.074979

H 3.646538 -3.727783 3.287166

H 4.435066 -3.799550 1.701462

H 5.242782 -1.625488 1.837057

H 4.927042 0.076536 1.512575

H 5.803062 -0.999163 -0.555904

H 4.105428 -0.541636 -0.822274

H 4.516083 -2.218699 -0.509763

H 3.542505 0.181871 3.747041

H 3.805563 -1.536569 4.036069

H 1.939807 -0.683439 5.485345

H 1.284822 -1.842604 4.320911

H 1.071261 -0.107594 4.049246

C -4.280093 -1.432403 -2.699556

C -4.527106 -1.406192 -4.017423

H -4.557537 -0.466825 -4.563620

H -4.712142 -2.319033 -4.578896

C -4.166774 -2.699284 -1.944317

C -4.779136 -2.891149 -0.695898

C -3.413094 -3.749285 -2.490805

C -4.655248 -4.108105 -0.027152

H -5.357373 -2.081523 -0.263750

C -3.298200 -4.968614 -1.824933

H -2.893722 -3.588953 -3.431371

C -3.921302 -5.154389 -0.589628

H -5.141243 -4.241279 0.936649

H -2.706380 -5.768132 -2.264164

H -3.827055 -6.102173 -0.065287

Structure: TS_18/19_

Symbol X Y Z

C -2.417503 2.757081 -2.883850

C -1.919477 1.515350 -3.258612

C -2.704075 0.342789 -3.160156

C -3.993060 0.452479 -2.540066

C -4.464266 1.722072 -2.179710

C -3.711674 2.875804 -2.361433

H -1.799501 3.643234 -3.018088

H -0.932635 1.422022 -3.704083

H -5.440976 1.770380 -1.704448

H -4.109922 3.847508 -2.081638

C -4.732947 -0.745418 -2.012831

O -5.437827 -0.604912 -0.989860

O -2.213397 -0.785604 -3.652604

H -0.891950 -1.482471 -2.802439

Au 0.647199 -0.041790 0.901118

P 1.636978 1.950528 0.014414

C 3.470411 2.023470 0.177556

C 4.162984 3.283950 -0.332020

C 1.314342 2.314600 -1.751425

C 1.899842 1.258343 -2.684295

C 1.049496 3.465868 0.866141

C 1.163369 3.358115 2.384865

H 3.851242 1.137426 -0.342654

H 3.684121 1.861959 1.241306

H 5.247907 3.200528 -0.199199

H 3.834428 4.177700 0.208417

H 3.974483 3.446144 -1.398472

H 1.696066 3.314791 -1.992258

H 0.223796 2.348476 -1.848975

H 1.609205 1.456500 -3.721773

H 1.543035 0.258292 -2.419465

H 2.994794 1.246623 -2.646124

H 0.004080 3.594381 0.563692

H 1.599129 4.335919 0.487346

H 0.740526 4.244083 2.871239

H 2.207742 3.269571 2.704700

H 0.625548 2.477197 2.755090

P -1.700740 -0.318182 1.116420

C -2.214994 -0.558256 2.866424

C -3.709573 -0.786193 3.080018

H -3.934141 -0.859462 4.150643

H -4.309832 0.031478 2.667968

H -4.045647 -1.713423 2.605533

H -1.631671 -1.402374 3.250355

H -1.868856 0.325358 3.416459

C -2.432364 -1.743433 0.240288

C -1.888075 -3.087132 0.701078

H -2.397762 -3.893937 0.167778

H -0.817023 -3.164449 0.498300

H -2.047677 -3.243374 1.774445

H -3.518798 -1.689288 0.362151

H -2.250076 -1.594979 -0.828439

C -2.705630 1.093365 0.527993

C -2.596631 2.323514 1.420303

H -3.039879 3.192743 0.922034

H -3.118725 2.182617 2.373000

H -1.551666 2.560495 1.648314

H -2.336102 1.317192 -0.477522

H -3.746258 0.771077 0.410364

C -0.068531 -1.929985 -2.203427

N 0.781595 -2.395257 -1.566207

P 2.225056 -1.596824 1.811787

C 1.732559 -3.363881 1.740507

C 2.734151 -4.383902 2.273256

C 3.924540 -1.556919 1.115820

C 3.958503 -1.840993 -0.382842

C 2.537211 -1.249676 3.589991

C 1.257689 -1.201682 4.420046

H 1.494095 -3.555851 0.688436

H 0.781191 -3.427592 2.281202

H 2.308527 -5.392349 2.215131

H 2.994822 -4.197711 3.320929

H 3.660247 -4.386633 1.689119

H 4.557995 -2.262643 1.667903

H 4.310111 -0.552788 1.327026

H 4.973164 -1.713239 -0.776905

H 3.286593 -1.169510 -0.926857

H 3.633666 -2.861728 -0.606717

H 3.047304 -0.278798 3.622306

H 3.239996 -1.993357 3.985653

H 1.482624 -0.971457 5.467516

H 0.725289 -2.158907 4.397546

H 0.577704 -0.432768 4.038925

C -4.390873 -2.003297 -2.637176

C -3.798248 -1.859443 -3.890447

H -4.143998 -1.068554 -4.549105

H -3.394262 -2.719443 -4.417131

C -4.406450 -3.287037 -1.924361

C -5.269089 -3.522612 -0.832950

C -3.542085 -4.340196 -2.294082

C -5.265360 -4.742857 -0.158676

H -5.936462 -2.727816 -0.521720

C -3.550946 -5.562217 -1.627433

H -2.830930 -4.199195 -3.103319

C -4.413690 -5.777661 -0.549724

H -5.944912 -4.886892 0.679156

H -2.864958 -6.345809 -1.942630

H -4.418216 -6.729949 -0.025159

Structure: 19

Symbol X Y Z

C -3.493319 2.191325 -4.384713

C -2.560100 1.264004 -3.924251

C -2.986187 -0.014564 -3.566900

C -4.343260 -0.378673 -3.610626

C -5.258600 0.573058 -4.072778

C -4.843957 1.841168 -4.474252

H -3.163193 3.183164 -4.683858

H -1.504817 1.511803 -3.847576

H -6.308194 0.291934 -4.077863

H -5.564502 2.559974 -4.851986

C -4.814109 -1.662486 -3.000817

O -6.057116 -1.756306 -2.721297

O -2.047604 -0.893580 -3.104349

H -1.378979 -0.722630 -1.330766

Au -7.826172 0.352611 -0.810669

P -8.010363 2.695738 -1.183782

C -6.405732 3.589727 -1.269645

C -6.468520 5.110449 -1.375563

C -8.957031 3.331326 -2.629167

C -8.210003 3.159821 -3.947979

C -8.923404 3.476468 0.209050

C -8.379321 3.094395 1.581524

H -5.868638 3.156547 -2.122443

H -5.842057 3.284940 -0.381006

H -5.457085 5.523377 -1.464202

H -6.930536 5.560958 -0.490762

H -7.034946 5.437825 -2.253777

H -9.214275 4.384940 -2.460130

H -9.897565 2.767756 -2.638570

H -8.873151 3.339529 -4.800877

H -7.805183 2.147594 -4.040656

H -7.372308 3.859962 -4.021861

H -9.965598 3.148765 0.108664

H -8.917153 4.564058 0.066770

H -8.930749 3.610972 2.375232

H -7.320666 3.357531 1.685794

H -8.474420 2.016866 1.745024

P -9.142052 -1.365466 -1.791357

C -10.938262 -1.210682 -1.415630

C -11.835310 -2.289857 -2.016718

H -12.881181 -2.123923 -1.732543

H -11.786021 -2.291496 -3.110756

H -11.552905 -3.289595 -1.670291

H -11.026494 -1.192463 -0.322374

H -11.239659 -0.213865 -1.759369

C -8.701054 -3.063134 -1.262825

C -9.076066 -3.355997 0.184954

H -8.600693 -4.281913 0.527753

H -8.749363 -2.547242 0.845571

H -10.158333 -3.468581 0.313100

H -9.161286 -3.790374 -1.943644

H -7.618340 -3.112447 -1.409805

C -9.047244 -1.467603 -3.618109

C -9.698251 -0.281736 -4.320524

H -9.461727 -0.287092 -5.390443

H -10.789620 -0.296985 -4.221387

H -9.341146 0.664069 -3.903316

H -7.971679 -1.526709 -3.822291

H -9.499991 -2.413269 -3.941889

C -1.041693 -0.695597 -0.295201

N -0.699859 -0.664602 0.811135

P -6.192342 -0.279304 0.845409

C -5.605335 -1.993677 0.589246

C -4.381567 -2.444971 1.375392

C -4.675252 0.751304 0.825050

C -4.044296 0.786806 -0.558864

C -6.673189 -0.167471 2.617557

C -7.973264 -0.887368 2.958416

H -5.445227 -2.083037 -0.490121

H -6.465003 -2.637929 0.805087

H -4.172688 -3.495504 1.149159

H -4.524134 -2.352138 2.458670

H -3.489942 -1.873505 1.100451

H -3.968762 0.369479 1.572599

H -4.976373 1.755233 1.149678

H -3.149816 1.418217 -0.571191

H -4.743682 1.170624 -1.307112

H -3.759098 -0.218158 -0.882393

H -6.757115 0.902009 2.843095

H -5.843198 -0.551997 3.223490

H -8.255228 -0.704985 4.001703

H -7.875833 -1.970047 2.828479

H -8.795119 -0.543878 2.319175

C -3.797620 -2.576794 -2.700025

C -2.451016 -2.273663 -3.285090

C -4.001475 -3.818556 -1.968280

C -5.275787 -4.431283 -1.898849

C -2.954855 -4.468072 -1.271361

C -5.493953 -5.584240 -1.151962

H -6.087194 -3.967771 -2.445016

C -3.174295 -5.627013 -0.531886

H -1.954421 -4.043788 -1.270433

C -4.448448 -6.195583 -0.453655

H -6.492876 -6.016624 -1.122985

H -2.341867 -6.081768 0.001575

H -4.619356 -7.096757 0.130037

H -1.648286 -2.857696 -2.835442

H -2.415107 -2.449903 -4.373887

Structure: TS_19/20_

Symbol X Y Z

C -4.173854 0.521343 -6.947071

C -3.247042 0.344803 -5.924778

C -3.570788 -0.468386 -4.833304

C -4.834297 -1.073109 -4.740056

C -5.745363 -0.888975 -5.787276

C -5.424399 -0.107898 -6.891135

H -3.915290 1.144783 -7.799675

H -2.268303 0.815154 -5.957825

H -6.720698 -1.357890 -5.691571

H -6.140432 0.024095 -7.697840

C -5.249788 -1.791345 -3.498684

O -6.440111 -2.144891 -3.383707

O -2.641479 -0.616862 -3.861663

H -4.285594 -0.638380 -1.743233

Au -5.819154 1.946375 0.601280

P -6.070137 3.470290 -1.237881

C -4.445498 4.158177 -1.753944

C -4.426117 5.036364 -3.001059

C -6.815152 2.892924 -2.811861

C -6.107580 1.667953 -3.376795

C -7.112837 4.934841 -0.849535

C -6.671576 5.681964 0.404778

H -3.781184 3.294040 -1.866158

H -4.069692 4.710063 -0.886154

H -3.422423 5.450597 -3.151938

H -5.121754 5.879669 -2.922983

H -4.682429 4.467698 -3.900540

H -6.806701 3.724626 -3.527625

H -7.867588 2.671488 -2.601765

H -6.595709 1.320144 -4.291699

H -6.115628 0.844462 -2.658333

H -5.061016 1.877061 -3.620345

H -8.131592 4.548030 -0.722541

H -7.131733 5.603310 -1.719500

H -7.371573 6.490778 0.642684

H -5.680901 6.131466 0.277052

H -6.628533 5.004955 1.264979

P -7.036780 -0.072270 0.872260

C -8.563856 0.141281 1.877750

C -9.415248 -1.113881 2.053553

H -10.290417 -0.897205 2.677322

H -9.779046 -1.494292 1.093617

H -8.853581 -1.918996 2.538927

H -8.248670 0.533553 2.851017

H -9.140057 0.942339 1.399482

C -6.142817 -1.459992 1.664372

C -5.876415 -1.258100 3.149531

H -5.199856 -2.034280 3.524062

H -5.413884 -0.285767 3.338560

H -6.798041 -1.303552 3.740418

H -6.702780 -2.384755 1.488876

H -5.203958 -1.562390 1.110707

C -7.630012 -0.839309 -0.678749

C -8.684265 -0.003883 -1.393272

H -8.819208 -0.359722 -2.419634

H -9.653860 -0.052306 -0.885034

H -8.384769 1.047952 -1.434483

H -6.753526 -0.983706 -1.315782

H -8.005707 -1.845237 -0.457936

C -3.892479 0.370790 -0.954418

N -3.022692 1.036821 -0.535234

P -4.327235 2.799595 2.291271

C -3.100362 1.579144 2.908025

C -2.230487 2.023042 4.080140

C -3.291550 4.273827 1.894817

C -2.099608 3.970321 0.989325

C -5.215113 3.407501 3.784907

C -6.091525 2.353502 4.450695

H -2.499486 1.321633 2.028222

H -3.655235 0.670495 3.159716

H -1.498848 1.243726 4.323498

H -2.826130 2.206816 4.981021

H -1.672291 2.938065 3.855075

H -2.956168 4.728564 2.836176

H -3.966483 4.996392 1.422871

H -1.653276 4.901397 0.621300

H -2.387060 3.355254 0.133591

H -1.321982 3.421865 1.530966

H -5.826532 4.253862 3.447747

H -4.480274 3.811666 4.492287

H -6.659956 2.788110 5.280694

H -5.493211 1.529369 4.853443

H -6.802143 1.931420 3.733426

C -4.224565 -1.901220 -2.480336

C -2.816544 -1.778505 -3.039303

C -4.360066 -2.829521 -1.329833

C -5.444234 -3.716732 -1.173440

C -3.389320 -2.819217 -0.305242

C -5.534892 -4.558628 -0.067178

H -6.217928 -3.726655 -1.931176

C -3.484536 -3.660687 0.802136

H -2.551388 -2.130979 -0.356693

C -4.558046 -4.541726 0.930407

H -6.382892 -5.235769 0.015085

H -2.715924 -3.618827 1.570894

H -4.634806 -5.199322 1.792896

H -2.075243 -1.657275 -2.249671

H -2.536828 -2.659452 -3.641221

Structure: 20

Symbol X Y Z

C -1.260278 8.133918 -3.596815

C -2.552746 7.790353 -3.223643

C -2.773060 7.162103 -1.992462

C -1.687701 6.863378 -1.146059

C -0.390469 7.231114 -1.542822

C -0.169797 7.862346 -2.755707

H -1.097260 8.625736 -4.552307

H -3.404144 8.007988 -3.861370

H 0.430976 6.988703 -0.874816

H 0.836087 8.138452 -3.057583

C -1.902791 6.117191 0.103148

O -1.022487 5.919241 0.929456

H -4.327349 7.436952 0.268695

O -4.054604 6.859297 -1.682541

H -3.497177 5.489425 1.351519

C -3.804491 2.510779 -2.028746

C -3.835723 3.823062 -1.552951

C -3.323972 4.129894 -0.286658

C -2.772226 3.099340 0.486351

C -2.737917 1.790726 0.009888

C -3.255958 1.491074 -1.252016

H -4.209430 2.289069 -3.012963

H -4.259080 4.605627 -2.174057

H -2.363180 3.330061 1.466885

H -2.309066 1.004160 0.625540

H -3.231928 0.470593 -1.625386

C -3.307140 5.541119 0.273638

C -4.319812 6.509723 -0.319827

H -5.325504 6.083964 -0.321993

Structure: TS_16-bra/22_

Symbol X Y Z

C -1.440255 4.432121 -2.191360

C -1.741890 5.641524 -1.573896

C -2.328072 5.675661 -0.301916

C -2.603915 4.479892 0.384994

C -2.288230 3.279006 -0.262159

C -1.718051 3.234812 -1.531958

H -0.990648 4.430828 -3.181310

H -1.525755 6.581251 -2.080806

H -2.500073 2.359834 0.275856

H -1.492538 2.279270 -1.997929

C -3.246864 4.304069 1.765720

O -2.871596 3.296422 2.424431

H -6.592828 4.183652 2.719963

O -2.620395 6.867470 0.277908

H -2.430019 7.583085 -0.346081

Au -0.309032 5.398916 4.866018

P 1.051330 7.331101 4.475164

C 2.640825 6.955926 3.621147

C 3.559315 8.132366 3.304042

C 0.297463 8.678531 3.480545

C 0.073697 8.311363 2.016955

C 1.597022 8.229006 5.984335

C 2.178066 7.297455 7.043888

H 2.365896 6.421263 2.705964

H 3.155148 6.222712 4.254251

H 4.447463 7.785292 2.762804

H 3.906742 8.636561 4.211831

H 3.060884 8.877499 2.675136

H 0.925691 9.573944 3.575844

H -0.665137 8.891351 3.959282

H -0.367005 9.160421 1.481334

H -0.620627 7.473077 1.931503

H 1.009779 8.050222 1.510627

H 0.711101 8.748566 6.365829

H 2.317652 9.006877 5.704051

H 2.420494 7.847500 7.960262

H 3.097156 6.814907 6.692788

H 1.464247 6.505536 7.298663

P -2.025855 5.343479 6.518730

C -1.494509 4.458603 8.045386

C -2.542140 4.307231 9.144639

H -2.108129 3.818033 10.024793

H -2.937189 5.277041 9.466606

H -3.387351 3.696583 8.810698

H -1.128585 3.477996 7.718676

H -0.612844 4.996655 8.414935

C -3.613153 4.546511 6.057463

C -3.486862 3.077132 5.674712

H -4.481669 2.623916 5.592320

H -3.001659 2.984500 4.700426

H -2.924550 2.500166 6.418728

H -4.304222 4.685246 6.898910

H -4.001433 5.125427 5.212398

C -2.626130 6.975601 7.117954

C -1.617122 7.714598 7.988688

H -1.934706 8.748776 8.163771

H -1.496273 7.234295 8.965869

H -0.634578 7.741098 7.510137

H -2.847743 7.538049 6.203893

H -3.573211 6.835324 7.654508

C -2.679572 5.930072 2.763069

N -2.566285 6.724721 3.620576

P 0.473808 3.411319 3.802287

C -0.269790 1.841580 4.406407

C 0.402840 0.542333 3.971578

C 0.307083 3.271883 1.987312

C 0.722170 4.526727 1.230547

C 2.276270 3.213563 4.108389

C 2.627022 3.286930 5.591932

H -1.303535 1.883838 4.047736

H -0.316931 1.909531 5.499404

H -0.169970 -0.318863 4.335876

H 1.418775 0.452950 4.371728

H 0.460047 0.459298 2.881147

H -0.761556 3.080283 1.838096

H 0.870026 2.396017 1.640039

H 0.490456 4.418299 0.165650

H 1.796004 4.729424 1.324078

H 0.172253 5.395747 1.602219

H 2.771178 4.021084 3.557872

H 2.617946 2.270084 3.664838

H 3.710671 3.224759 5.745654

H 2.162979 2.468048 6.153698

H 2.271732 4.228325 6.026835

C -5.979496 6.597596 -1.188659

C -5.473516 5.572147 -0.391306

C -5.280239 5.766456 0.982727

C -5.617908 7.002981 1.544380

C -6.126602 8.029435 0.749343

C -6.304198 7.832042 -0.621478

H -6.123896 6.431676 -2.253838

H -5.219231 4.612732 -0.834143

H -5.452574 7.158645 2.606758

H -6.379752 8.986607 1.199664

H -6.698706 8.632497 -1.243065

C -4.736466 4.678013 1.830438

C -5.528568 3.973836 2.646271

H -5.108532 3.171240 3.245077

Structure: TS_16-bra/21_

Symbol X Y Z

C -0.436845 6.850098 -1.477037

C -1.794839 7.138357 -1.500363

C -2.663689 6.539662 -0.567403

C -2.123410 5.662217 0.404098

C -0.760719 5.351357 0.380404

C 0.088452 5.938207 -0.548070

H 0.222411 7.323502 -2.201363

H -2.217231 7.806791 -2.245713

H -0.392325 4.661430 1.133828

H 1.148497 5.698480 -0.556666

C -2.977070 5.016672 1.420241

O -2.629815 3.967433 2.007603

H -6.587244 3.369762 0.431003

O -3.975145 6.706608 -0.637956

H -4.373541 5.704775 -0.262038

Au -0.473932 3.661807 3.944684

P 1.045084 5.478529 4.008663

C 2.116839 5.747502 2.538389

C 3.192345 6.824624 2.645617

C 0.273614 7.112899 4.325143

C -0.832830 7.434512 3.325145

C 2.232065 5.302030 5.397747

C 3.000283 3.983969 5.342305

H 1.433648 5.972743 1.714256

H 2.563235 4.773371 2.306693

H 3.726947 6.913564 1.692794

H 3.933719 6.590047 3.416618

H 2.765508 7.807161 2.871972

H 1.049238 7.888389 4.332209

H -0.136064 7.061310 5.341246

H -1.306872 8.392459 3.564022

H -1.607865 6.663077 3.348399

H -0.455422 7.491916 2.299408

H 1.641684 5.366215 6.317885

H 2.915142 6.159823 5.398572

H 3.654548 3.877059 6.214705

H 3.627399 3.922200 4.445958

H 2.311705 3.131260 5.323331

P -2.018212 3.639575 5.810569

C -2.395646 1.982693 6.521741

C -3.304443 1.966064 7.747806

H -3.477309 0.935541 8.079464

H -2.865512 2.512829 8.588997

H -4.281081 2.411537 7.532059

H -2.837075 1.395276 5.710013

H -1.430671 1.511314 6.740726

C -3.666070 4.386693 5.498634

C -4.546878 3.527675 4.593811

H -5.478619 4.057779 4.371234

H -4.041611 3.330218 3.644105

H -4.807365 2.574611 5.068134

H -4.151900 4.578660 6.464141

H -3.485064 5.358797 5.027113

C -1.388397 4.573390 7.266247

C -0.169652 3.900047 7.894782

H 0.323590 4.567624 8.610004

H -0.451382 2.989095 8.433083

H 0.565001 3.615891 7.132905

H -1.134231 5.574554 6.899900

H -2.189627 4.698433 8.005215

C -3.904831 5.910368 2.138980

N -4.583276 6.574811 2.812754

P -0.582978 1.721294 2.571461

C -2.198295 0.855118 2.681783

C -2.247263 -0.571615 2.145384

C -0.332148 1.887403 0.764337

C 1.062385 2.376026 0.387819

C 0.660278 0.475413 3.099494

C 0.525330 0.129348 4.580851

H -2.887727 1.514005 2.143957

H -2.504245 0.879745 3.731703

H -3.278778 -0.943035 2.156600

H -1.644549 -1.254834 2.753930

H -1.889762 -0.634627 1.113319

H -1.106675 2.585307 0.428924

H -0.544609 0.924637 0.286383

H 1.096468 2.650090 -0.672032

H 1.822001 1.605678 0.561339

H 1.346128 3.259997 0.969238

H 1.646019 0.912365 2.902905

H 0.571546 -0.420346 2.471993

H 1.297395 -0.584635 4.889408

H -0.450405 -0.316905 4.803088

H 0.625816 1.029211 5.198922

C -3.531844 0.840680 -0.883252

C -4.171859 1.878999 -0.209997

C -4.046097 3.213123 -0.637703

C -3.226127 3.468902 -1.752396

C -2.574472 2.433188 -2.418571

C -2.724749 1.112411 -1.989369

H -3.658093 -0.183011 -0.539764

H -4.782603 1.666748 0.664329

H -3.106526 4.489622 -2.102362

H -1.947976 2.658558 -3.278686

H -2.215535 0.304456 -2.509458

C -4.689324 4.307141 0.092999

C -5.924545 4.222917 0.608820

H -6.338003 5.027820 1.217859

Structure: 21

Symbol X Y Z

C -2.020440 -3.640689 -3.545813

C -2.613748 -2.634534 -2.823278

C -1.950881 -1.371707 -2.580407

C -0.611706 -1.255882 -3.131121

C -0.043648 -2.311984 -3.887752

C -0.719698 -3.490539 -4.096864

H -2.558930 -4.573689 -3.705023

H -3.614486 -2.752090 -2.413912

H 0.946906 -2.157959 -4.309426

H -0.274690 -4.292620 -4.678569

C 0.166778 -0.073316 -2.930033

O 1.337362 0.109817 -3.303305

O -2.503285 -0.449668 -1.918678

Au 0.319819 -0.227146 0.692034

P 1.754136 1.599365 1.306509

C 3.496864 1.128889 1.669950

C 4.400820 2.234272 2.208208

C 1.886403 3.020284 0.153114

C 2.655086 2.691371 -1.123896

C 1.200860 2.379230 2.877538

C 1.261954 1.406815 4.053572

H 3.900310 0.718033 0.739903

H 3.444454 0.286338 2.370225

H 5.416217 1.852128 2.365792

H 4.043826 2.624393 3.167152

H 4.470214 3.074338 1.509026

H 2.346864 3.860854 0.687848

H 0.859543 3.307352 -0.096980

H 2.640062 3.552206 -1.801395

H 2.207249 1.848773 -1.658321

H 3.704033 2.451447 -0.916303

H 0.172890 2.716686 2.705315

H 1.802330 3.274608 3.077837

H 0.774382 1.831647 4.938005

H 2.296615 1.172302 4.325507

H 0.759753 0.463253 3.812487

P -1.971643 -0.003286 1.345904

C -2.103674 0.276785 3.163199

C -3.518495 0.443708 3.710992

H -3.492937 0.594009 4.796786

H -4.020918 1.310576 3.269137

H -4.138636 -0.437014 3.513917

H -1.593510 -0.563925 3.648569

H -1.499717 1.162630 3.384279

C -3.056351 -1.454684 1.057140

C -2.672633 -2.652572 1.920061

H -3.170399 -3.562411 1.566606

H -1.592066 -2.828256 1.891517

H -2.952534 -2.505898 2.969081

H -4.100052 -1.163790 1.231423

H -2.958457 -1.669355 -0.010833

C -2.889130 1.398149 0.606483

C -2.325045 2.756663 1.005596

H -2.849131 3.556539 0.471213

H -2.428875 2.949048 2.079988

H -1.267289 2.826800 0.737491

H -2.820043 1.244706 -0.473801

H -3.945171 1.307348 0.890633

C -0.439148 1.085044 -2.230578

N -0.676497 2.135161 -1.786257

P 1.295741 -2.277505 -0.038204

C 0.133614 -3.621050 -0.502902

C 0.743769 -4.929207 -0.995674

C 2.542316 -2.288562 -1.383773

C 3.613412 -1.215719 -1.240509

C 2.241592 -2.953680 1.389759

C 1.414434 -3.046436 2.669349

H -0.527652 -3.198251 -1.264568

H -0.493526 -3.793229 0.377821

H -0.050388 -5.662492 -1.177606

H 1.437156 -5.365940 -0.267644

H 1.277283 -4.788663 -1.940069

H 1.992784 -2.140429 -2.316875

H 2.990797 -3.288968 -1.419419

H 4.298931 -1.249734 -2.094378

H 4.208127 -1.348925 -0.329958

H 3.157412 -0.223268 -1.220319

H 3.090121 -2.276090 1.539343

H 2.656528 -3.931879 1.115743

H 2.032300 -3.386603 3.508266

H 0.583892 -3.752570 2.563140

H 0.990826 -2.069754 2.931248

Structure: PhCHCH_2_

Symbol X Y Z

H -9.591126 8.849702 0.154771

H -9.634070 11.122460 -1.925129

C -9.093758 5.967893 -2.578795

C -9.266969 6.940092 -1.594435

C -9.366910 8.300973 -1.925870

C -9.287263 8.659423 -3.282972

C -9.114432 7.691183 -4.266864

C -9.016712 6.339757 -3.920785

H -9.019167 4.920776 -2.296557

H -9.326361 6.643921 -0.549107

H -9.360761 9.703615 -3.573670

H -9.055339 7.990197 -5.310421

H -8.881717 5.586064 -4.692125

C -9.548944 9.280498 -0.846154

C -9.664981 10.611020 -0.966084

H -9.796963 11.237301 -0.088358

Structure: M23

Symbol X Y Z

C -3.410264 -2.945415 -3.633238

C -3.755526 -1.618166 -3.401101

C -2.773424 -0.661332 -3.127171

C -1.421163 -1.040447 -3.049085

C -1.094262 -2.377686 -3.303427

C -2.067958 -3.327671 -3.595781

H -4.186656 -3.674192 -3.851731

H -4.790013 -1.288732 -3.439663

H -0.048641 -2.661228 -3.241896

H -1.781458 -4.358952 -3.783435

C -0.283920 -0.084707 -2.759744

O 0.830598 -0.752855 -2.228508

O -3.223931 0.607821 -2.890756

H -2.605469 1.265396 -3.258167

Au 0.498985 -0.116497 1.384563

P 1.883056 1.800563 1.724094

C 3.620318 1.376550 2.163927

C 4.566385 2.543822 2.429578

C 2.023140 2.986416 0.335138

C 2.762784 2.415387 -0.868932

C 1.338690 2.865747 3.116552

C 1.199093 2.093602 4.425564

H 3.993683 0.752912 1.344668

H 3.558803 0.718814 3.039342

H 5.573497 2.172939 2.652971

H 4.241245 3.145225 3.284957

H 4.644437 3.206499 1.561487

H 2.510214 3.897108 0.706397

H 0.996985 3.246441 0.053594

H 2.814263 3.162611 -1.665508

H 2.238190 1.547534 -1.276085

H 3.787655 2.118362 -0.619366

H 0.378270 3.293578 2.807448

H 2.036886 3.704431 3.226702

H 0.767679 2.726011 5.209474

H 2.170444 1.735854 4.784535

H 0.548967 1.219905 4.298490

P -1.865616 -0.134743 1.695617

C -2.269187 -1.115622 3.202002

C -3.750730 -1.302510 3.515999

H -3.875387 -1.855452 4.454388

H -4.268311 -0.343651 3.626385

H -4.259868 -1.869174 2.729684

H -1.773809 -2.085231 3.074695

H -1.750338 -0.623219 4.033613

C -2.853766 -0.903217 0.358072

C -2.379479 -2.298030 -0.020328

H -2.986804 -2.701269 -0.835916

H -1.343891 -2.265173 -0.366851

H -2.438857 -2.992043 0.826125

H -3.906006 -0.908997 0.668877

H -2.784251 -0.223989 -0.496732

C -2.750394 1.459388 1.924202

C -2.431578 2.155869 3.241999

H -2.866508 3.161263 3.262487

H -2.829728 1.604251 4.100650

H -1.352358 2.254888 3.383726

H -2.448817 2.075965 1.070296

H -3.828811 1.278729 1.829989

C -0.660009 0.963130 -1.783168

N -0.881800 1.789736 -0.996457

P 1.661588 -2.205830 1.155760

C 0.645737 -3.737089 1.301445

C 1.391387 -5.046135 1.548252

C 2.658624 -2.506370 -0.353409

C 3.686671 -1.411625 -0.619687

C 2.868335 -2.350063 2.535114

C 2.199956 -2.177913 3.897422

H 0.068694 -3.792514 0.371942

H -0.084508 -3.554001 2.097741

H 0.686892 -5.886062 1.543707

H 1.897891 -5.048836 2.518962

H 2.141486 -5.243876 0.775494

H 1.937705 -2.543240 -1.176439

H 3.142747 -3.487275 -0.272293

H 4.175538 -1.573645 -1.586464

H 4.468883 -1.389911 0.147310

H 3.206684 -0.429950 -0.649633

H 3.613686 -1.563685 2.378412

H 3.394944 -3.309836 2.466667

H 2.941164 -2.208203 4.703966

H 1.465705 -2.968365 4.089883

H 1.674535 -1.217207 3.952928

H 1.562447 -0.213354 -2.687236

C -1.706981 1.546665 -5.357876

C -2.536525 2.606253 -5.730978

C -2.205617 3.901443 -5.323282

C -1.052606 4.137792 -4.578208

C -0.218553 3.073618 -4.206976

C -0.570490 1.752039 -4.570097

H -1.947704 0.531885 -5.671473

H -3.423901 2.422655 -6.330307

H -2.842244 4.738179 -5.601565

H -0.773054 5.144745 -4.279353

C 0.296231 0.601856 -4.110611

O 1.597036 0.938519 -3.844013

H 0.217332 -0.222858 -4.845495

O 0.911839 3.311185 -3.524884

H 1.398606 2.390152 -3.527666

Structure: TS_23/24_

Symbol X Y Z

C -2.834569 -3.365022 -3.195971

C -3.364745 -2.080306 -3.163831

C -2.538225 -0.967909 -2.971016

C -1.153353 -1.140637 -2.796664

C -0.642564 -2.441782 -2.841696

C -1.460356 -3.550952 -3.035769

H -3.493860 -4.215770 -3.348533

H -4.430138 -1.907210 -3.288520

H 0.428777 -2.554226 -2.713092

H -1.029101 -4.548164 -3.062665

C -0.145822 -0.011146 -2.634224

O 1.059243 -0.409013 -2.104892

O -3.182053 0.236279 -2.917954

H -2.597628 0.968476 -3.180951

Au 0.446673 -0.080718 1.249851

P 1.862589 1.804137 1.678311

C 3.594986 1.349460 2.105829

C 4.546192 2.496830 2.432928

C 2.008793 3.031293 0.325234

C 2.747269 2.483986 -0.890921

C 1.347048 2.836148 3.106278

C 1.215356 2.026754 4.393506

H 3.969867 0.765325 1.260661

H 3.524800 0.648682 2.946828

H 5.554328 2.110727 2.623824

H 4.231740 3.047263 3.325760

H 4.618264 3.210083 1.605093

H 2.495695 3.933005 0.718178

H 0.982350 3.299009 0.050274

H 2.757840 3.230152 -1.690358

H 2.249314 1.588460 -1.272871

H 3.786889 2.229303 -0.655457

H 0.391123 3.289803 2.822984

H 2.060125 3.659964 3.231953

H 0.789229 2.635174 5.199032

H 2.189445 1.659156 4.734626

H 0.566365 1.155353 4.246027

P -1.896090 -0.010126 1.701199

C -2.212952 -0.914731 3.275171

C -3.675552 -1.084321 3.676538

H -3.749136 -1.566718 4.658256

H -4.195963 -0.122677 3.740521

H -4.218367 -1.709579 2.960335

H -1.720669 -1.888974 3.174413

H -1.652919 -0.378480 4.050942

C -3.045618 -0.784882 0.501357

C -2.758314 -2.255707 0.241967

H -3.519542 -2.682503 -0.416591

H -1.793662 -2.375373 -0.253732

H -2.746708 -2.841821 1.168279

H -4.064454 -0.641577 0.883120

H -2.970997 -0.204303 -0.423575

C -2.700706 1.629180 1.938938

C -2.364037 2.304879 3.263396

H -2.717674 3.341933 3.267470

H -2.830662 1.791979 4.111040

H -1.286570 2.319180 3.440838

H -2.367687 2.235046 1.089471

H -3.786019 1.501065 1.839448

C -0.685070 1.074381 -1.772637

N -1.035819 1.934076 -1.071632

P 1.570679 -2.169206 0.891397

C 0.496276 -3.655917 0.726093

C 1.165560 -5.023334 0.834841

C 2.769138 -2.383765 -0.480476

C 3.834074 -1.297243 -0.548526

C 2.567985 -2.509540 2.399849

C 1.724854 -2.464886 3.672280

H 0.001118 -3.546910 -0.245789

H -0.293188 -3.552711 1.478443

H 0.422647 -5.816044 0.687269

H 1.618771 -5.178676 1.819740

H 1.944847 -5.160953 0.078484

H 2.169399 -2.355878 -1.394376

H 3.226834 -3.376358 -0.389002

H 4.478550 -1.452683 -1.421006

H 4.475969 -1.293768 0.339673

H 3.365456 -0.315903 -0.650982

H 3.345243 -1.738245 2.435505

H 3.078646 -3.474650 2.294116

H 2.347684 -2.619053 4.560761

H 0.950505 -3.240349 3.670005

H 1.223205 -1.494945 3.771313

H 1.701301 0.209364 -2.936353

C -1.757498 1.522392 -5.271410

C -2.605101 2.574729 -5.620867

C -2.249446 3.878375 -5.267277

C -1.053819 4.127153 -4.596975

C -0.207566 3.069310 -4.247101

C -0.576211 1.741475 -4.555730

H -2.014579 0.502217 -5.552584

H -3.524551 2.378638 -6.165205

H -2.898099 4.710618 -5.529898

H -0.753361 5.139163 -4.338993

C 0.271392 0.586258 -4.103990

O 1.620074 0.883640 -3.949315

H 0.122745 -0.264823 -4.788294

O 0.959661 3.336750 -3.626656

H 1.459545 2.459529 -3.622991

Structure: M24

Symbol X Y Z

C -2.407306 -3.586337 -2.836267

C -3.209766 -2.464617 -2.999773

C -2.685338 -1.174215 -2.845663

C -1.327989 -1.006398 -2.517962

C -0.542965 -2.150187 -2.357824

C -1.056658 -3.432923 -2.512560

H -2.836593 -4.577195 -2.961555

H -4.263383 -2.558594 -3.247936

H 0.495369 -1.974512 -2.099290

H -0.416265 -4.302485 -2.386767

C -0.571195 0.322208 -2.378949

O 0.548401 0.258544 -1.612527

O -3.588237 -0.167485 -3.009094

H -3.153658 0.699028 -3.064718

Au 0.369666 -0.212840 0.959271

P 1.879342 1.645320 1.172834

C 3.384708 1.516139 0.125714

C 4.402237 2.647795 0.234104

C 1.242769 3.336734 0.804971

C 1.019655 3.605204 -0.678602

C 2.551059 1.905659 2.869113

C 3.340757 0.717794 3.404704

H 3.015338 1.402815 -0.899328

H 3.846134 0.556416 0.378857

H 5.232029 2.478364 -0.462150

H 4.827713 2.714896 1.241360

H 3.963229 3.620687 -0.009391

H 1.940841 4.067326 1.234479

H 0.299295 3.437750 1.351682

H 0.538481 4.578268 -0.824639

H 0.375356 2.843067 -1.114755

H 1.961877 3.611610 -1.236415

H 1.692038 2.113748 3.518114

H 3.167403 2.813258 2.861862

H 3.699996 0.915968 4.420987

H 4.214345 0.498820 2.780580

H 2.716358 -0.178154 3.436281

P -1.884761 0.087858 1.687874

C -2.087808 -0.789096 3.298253

C -3.462313 -0.699755 3.954077

H -3.458143 -1.213932 4.922474

H -3.760909 0.338722 4.133141

H -4.235763 -1.167561 3.336153

H -1.809967 -1.833621 3.119176

H -1.311829 -0.387067 3.960244

C -3.304267 -0.511672 0.689602

C -3.255874 -2.010940 0.426496

H -4.109186 -2.318548 -0.185609

H -2.347281 -2.278024 -0.118388

H -3.283170 -2.590685 1.356422

H -4.229235 -0.229471 1.208330

H -3.286816 0.045137 -0.251299

C -2.374866 1.816948 2.079970

C -1.574491 2.403319 3.238977

H -1.719732 3.487207 3.305970

H -1.872733 1.967658 4.198641

H -0.503173 2.214209 3.111584

H -2.211200 2.384011 1.157543

H -3.451074 1.853634 2.291335

C -1.488654 1.362123 -1.812655

N -2.160698 2.174482 -1.320035

P 1.170265 -2.472846 0.992554

C -0.121753 -3.781787 1.067847

C 0.359378 -5.219773 1.237064

C 2.280010 -3.074207 -0.352485

C 3.186633 -1.978025 -0.901816

C 2.176867 -2.787793 2.500458

C 1.394565 -2.532036 3.785624

H -0.695292 -3.674816 0.141046

H -0.804904 -3.501492 1.877274

H -0.495135 -5.906773 1.232020

H 0.892110 -5.365693 2.182914

H 1.027919 -5.521420 0.423745

H 1.637496 -3.473402 -1.144143

H 2.865516 -3.918456 0.032544

H 3.798518 -2.359646 -1.727650

H 3.868696 -1.608121 -0.128298

H 2.591295 -1.133356 -1.264215

H 3.044551 -2.121810 2.434116

H 2.560053 -3.815204 2.473499

H 2.045486 -2.609542 4.663770

H 0.582525 -3.257177 3.908487

H 0.945355 -1.532545 3.779162

H 1.284293 1.279047 -2.684250

C -1.942075 0.643808 -5.513341

C -2.984950 1.191744 -6.255768

C -3.215060 2.568119 -6.191337

C -2.398047 3.382431 -5.412346

C -1.349181 2.829094 -4.669816

C -1.129674 1.434971 -4.692818

H -1.756137 -0.427680 -5.547730

H -3.610721 0.553302 -6.872756

H -4.026842 3.013904 -6.760861

H -2.549736 4.457462 -5.369655

C -0.086955 0.780523 -3.836975

O 1.028193 1.623114 -3.601293

H 0.249862 -0.151067 -4.312706

O -0.564191 3.669113 -3.954881

H 0.201556 3.131249 -3.632322

Structure: TS_24/25_

Symbol X Y Z

C -3.409551 -3.098141 -2.373194

C -3.853885 -1.784720 -2.307824

C -2.952785 -0.712455 -2.377285

C -1.571131 -0.963382 -2.495504

C -1.152120 -2.300760 -2.547335

C -2.044061 -3.366304 -2.493599

H -4.129413 -3.911528 -2.327957

H -4.910589 -1.552922 -2.206321

H -0.087948 -2.487462 -2.652000

H -1.680168 -4.388751 -2.548473

C -0.463060 0.047927 -2.697055

O 0.659988 -0.169660 -2.160080

O -3.499827 0.519702 -2.297376

H -2.771396 1.183633 -2.307560

Au 0.292096 -0.039259 1.041322

P 1.800764 1.770252 1.588765

C 3.569083 1.293639 1.789007

C 4.537256 2.409011 2.172681

C 1.837520 3.157894 0.394555

C 2.364151 2.716813 -0.968525

C 1.421287 2.577012 3.197623

C 1.572756 1.625799 4.382141

H 3.868572 0.832365 0.843908

H 3.586936 0.491377 2.535885

H 5.557694 2.015585 2.250026

H 4.283786 2.856748 3.139405

H 4.548792 3.208245 1.424386

H 2.426480 3.984154 0.812105

H 0.802000 3.495640 0.285269

H 2.249210 3.524539 -1.699024

H 1.797796 1.853820 -1.332254

H 3.425867 2.446734 -0.928971

H 0.391567 2.938026 3.127461

H 2.061912 3.459132 3.319636

H 1.216096 2.095946 5.305404

H 2.617661 1.337840 4.539571

H 0.996187 0.707362 4.227336

P -1.984170 0.301002 1.677328

C -1.971219 0.080510 3.510496

C -3.305348 0.208011 4.239312

H -3.156817 0.097475 5.319939

H -3.769402 1.185269 4.069445

H -4.016788 -0.562869 3.926079

H -1.516504 -0.899672 3.698843

H -1.256390 0.819058 3.890265

C -3.316518 -0.819877 1.096074

C -2.937718 -2.291935 1.101282

H -3.791373 -2.911054 0.805608

H -2.132943 -2.474000 0.387016

H -2.603912 -2.626613 2.091080

H -4.205038 -0.629629 1.710772

H -3.561717 -0.493951 0.081181

C -2.818302 1.916833 1.420687

C -2.075914 3.115093 1.990834

H -2.637176 4.036343 1.799137

H -1.931411 3.033260 3.074559

H -1.102550 3.217885 1.505510

H -2.920494 2.028189 0.337325

H -3.829249 1.837490 1.840300

C -1.099079 1.734351 -1.804259

N -0.970970 2.700359 -1.154974

P 1.395793 -2.121679 0.616713

C 0.346748 -3.632018 0.544468

C 1.054384 -4.978048 0.414938

C 2.482943 -2.291997 -0.852090

C 3.493853 -1.162897 -1.005384

C 2.534290 -2.463634 2.021426

C 1.813365 -2.459147 3.366746

H -0.337065 -3.475353 -0.296371

H -0.275919 -3.608560 1.445603

H 0.316137 -5.788740 0.412356

H 1.741676 -5.164538 1.247289

H 1.623563 -5.051762 -0.517076

H 1.815912 -2.299373 -1.717926

H 2.987716 -3.264096 -0.795649

H 4.066014 -1.290528 -1.931387

H 4.210011 -1.141861 -0.176656

H 2.978736 -0.201116 -1.058201

H 3.295839 -1.677289 1.997586

H 3.052694 -3.415376 1.851934

H 2.523513 -2.573153 4.193511

H 1.086734 -3.276110 3.437025

H 1.270694 -1.517830 3.510962

H 1.338119 1.042820 -3.409243

C -2.537858 0.483794 -5.315849

C -3.699461 1.046834 -5.833833

C -3.840269 2.436951 -5.827100

C -2.822374 3.241678 -5.327258

C -1.652446 2.671840 -4.808556

C -1.515434 1.268983 -4.769914

H -2.417631 -0.597540 -5.312572

H -4.483259 0.411305 -6.236297

H -4.741199 2.898364 -6.224193

H -2.905307 4.325079 -5.334719

C -0.331063 0.575842 -4.163204

O 0.841950 1.375161 -4.196472

H -0.147054 -0.351031 -4.732937

O -0.686369 3.517564 -4.382375

H 0.083222 2.965535 -4.113820

Structure: 25

Symbol X Y Z

C -2.026389 -4.133446 -3.470264

C -2.358571 -3.057206 -2.660894

C -1.693591 -1.824923 -2.783706

C -0.704191 -1.687224 -3.784469

C -0.340590 -2.807605 -4.551239

C -0.995050 -4.024381 -4.410818

H -2.559941 -5.073771 -3.354297

H -3.129116 -3.143154 -1.899723

H 0.446290 -2.689134 -5.291910

H -0.718080 -4.871233 -5.031856

C -0.052119 -0.409027 -4.158340

O 1.164240 -0.299332 -4.256932

O -2.001964 -0.866785 -1.910930

H -1.256711 -0.143970 -1.845809

Au 0.389070 0.338492 1.220575

P 2.015697 1.730668 2.278329

C 3.615178 0.881854 2.614373

C 4.661270 1.663805 3.402970

C 2.501947 3.327497 1.508799

C 3.346559 3.166890 0.247613

C 1.443180 2.263994 3.942403

C 1.052482 1.088150 4.833044

H 4.002810 0.585444 1.632900

H 3.358229 -0.051953 3.128182

H 5.576627 1.070909 3.515444

H 4.307072 1.914247 4.408624

H 4.933539 2.597774 2.900161

H 3.024721 3.928505 2.264568

H 1.565236 3.845826 1.273297

H 3.577921 4.150695 -0.177016

H 2.800800 2.593608 -0.506169

H 4.298553 2.666656 0.458108

H 0.578715 2.914323 3.763687

H 2.214246 2.882010 4.419186

H 0.605174 1.438132 5.770169

H 1.922278 0.473221 5.089731

H 0.325151 0.443103 4.327468

P -1.901871 0.929233 1.465882

C -2.318103 1.584032 3.136884

C -3.769051 1.999806 3.360640

H -3.908707 2.364303 4.385307

H -4.067013 2.804642 2.680581

H -4.458808 1.162161 3.211935

H -2.020379 0.810156 3.853926

H -1.641645 2.428394 3.309690

C -3.106580 -0.429680 1.208818

C -2.951402 -1.536336 2.248545

H -3.536922 -2.419667 1.969841

H -1.902379 -1.841510 2.338656

H -3.289726 -1.212202 3.239278

H -4.126727 -0.025133 1.210628

H -2.906779 -0.805708 0.199667

C -2.461255 2.228443 0.305125

C -1.661218 3.518976 0.438599

H -2.014236 4.256711 -0.289258

H -1.751003 3.958113 1.439485

H -0.604456 3.326163 0.230211

H -2.329857 1.817247 -0.700042

H -3.535298 2.398516 0.450622

C -0.037673 0.937827 -1.621951

N 0.775423 1.763642 -1.430596

P 1.124810 -1.781085 0.424705

C -0.187796 -3.038406 0.147601

C 0.216117 -4.292020 -0.622179

C 2.055760 -1.725383 -1.151742

C 3.204849 -0.723275 -1.125859

C 2.283195 -2.614065 1.586351

C 1.727283 -2.688904 3.006168

H -1.000263 -2.513738 -0.362524

H -0.568734 -3.296274 1.142990

H -0.615258 -5.005514 -0.643450

H 1.076085 -4.797333 -0.167337

H 0.464420 -4.056682 -1.661174

H 1.337679 -1.442001 -1.927431

H 2.414097 -2.734942 -1.387211

H 3.699743 -0.695199 -2.102803

H 3.960561 -0.984945 -0.376152

H 2.827318 0.279784 -0.910545

H 3.208400 -2.027198 1.574311

H 2.531197 -3.612620 1.204676

H 2.468091 -3.108843 3.696336

H 0.832483 -3.319153 3.057708

H 1.449235 -1.691634 3.365844

H 0.786997 1.633113 -4.534210

C -3.275309 0.023760 -4.699018

C -4.592741 0.102757 -4.261111

C -4.963611 1.151328 -3.415500

C -4.027664 2.105878 -3.036859

C -2.698007 2.018017 -3.470589

C -2.303075 0.943766 -4.290346

H -2.979261 -0.784829 -5.364320

H -5.320553 -0.637543 -4.581063

H -5.988731 1.235612 -3.062770

H -4.301345 2.941955 -2.399130

C -0.891418 0.734358 -4.774126

O -0.122371 1.921263 -4.758455

H -0.968389 0.379035 -5.819904

O -1.862368 3.004020 -3.073955

H -0.974251 2.812873 -3.446902

Structure: 26

Symbol X Y Z

C -3.546014 -2.105545 -1.529346

C -3.757961 -2.016921 -2.902619

C -2.702572 -1.684760 -3.754135

C -1.423937 -1.439980 -3.225930

C -1.225082 -1.566474 -1.844016

C -2.275989 -1.884307 -0.992108

H -4.379326 -2.356112 -0.878589

H -4.744109 -2.211336 -3.319252

H -0.228813 -1.385726 -1.451452

H -2.109626 -1.956453 0.078233

C -0.256152 -1.052223 -4.049040

O 0.856914 -1.517316 -3.838200

O -2.859816 -1.630554 -5.105154

H -3.774971 -1.846151 -5.340157

H 1.457475 -0.462064 -5.374630

C -2.382175 1.535763 -4.738641

C -3.011935 2.607754 -4.111132

C -2.267138 3.445870 -3.277645

C -0.905166 3.226079 -3.095537

C -0.276986 2.149905 -3.729090

C -1.022735 1.277243 -4.543825

H -2.950931 0.871193 -5.382532

H -4.070681 2.789241 -4.272972

H -2.744676 4.285354 -2.778833

H -0.305522 3.880948 -2.469757

C -0.388342 0.036580 -5.130125

O 0.911985 0.310261 -5.634390

H -1.028973 -0.355890 -5.928395

O 1.054499 1.977855 -3.524378

H 1.379235 1.391658 -4.242632

Structure: PBu_3_

Symbol X Y Z

P 11.945462 10.919650 2.159177

C 13.598717 11.519031 2.763220

C 11.842154 11.733570 0.493864

C 12.349179 9.191570 1.613514

C 14.805143 11.369450 1.840788

H 13.459261 12.574873 3.033303

H 13.782594 10.998495 3.713162

C 11.773060 13.258394 0.579143

H 12.684346 11.413146 -0.131804

H 10.929294 11.348602 0.017635

C 12.775659 8.279703 2.762669

H 13.121298 9.204912 0.831863

H 11.435705 8.796984 1.147015

C 16.095181 11.903469 2.464766

H 14.622509 11.898691 0.895850

H 14.948291 10.312437 1.578501

C 11.531295 13.942005 -0.770230

H 12.708177 13.645634 1.008339

H 10.973468 13.544229 1.276599

C 12.979972 6.828723 2.326776

H 13.710611 8.653036 3.203744

H 12.022841 8.317437 3.563404

C 17.302220 11.756497 1.541844

H 15.957158 12.961965 2.727870

H 16.284984 11.374622 3.409726

C 12.660911 13.734725 -1.777497

H 11.395793 15.018626 -0.598235

H 10.585624 13.576467 -1.195741

C 13.427636 5.924077 3.471985

H 13.724241 6.797190 1.518114

H 12.043122 6.446457 1.897088

H 17.148207 12.302043 0.602188

H 18.215355 12.144965 2.008012

H 17.478180 10.703399 1.288185

H 12.767928 12.680542 -2.057609

H 12.479908 14.302718 -2.697597

H 13.621205 14.067537 -1.362422

H 14.378888 6.268409 3.897260

H 13.566614 4.889595 3.136556

H 12.686527 5.916768 4.281170

Structure: 1-Bu

Symbol X Y Z

P 12.128425 10.862265 2.002174

C 13.691876 11.497265 2.711016

C 11.906818 11.726891 0.407578

C 12.440430 9.119772 1.546626

C 14.923096 11.379603 1.815022

H 13.504347 12.543236 2.983192

H 13.842223 10.962802 3.657038

C 11.766100 13.241408 0.570976

H 12.748219 11.467213 -0.244910

H 11.000851 11.306700 -0.047697

C 12.779555 8.239662 2.749447

H 13.239479 9.086631 0.795342

H 11.523780 8.763737 1.059660

C 16.178204 11.935254 2.488683

H 14.754542 11.917607 0.872736

H 15.095101 10.328453 1.547839

C 11.422921 13.957602 -0.737689

H 12.700369 13.657439 0.972165

H 10.986073 13.450097 1.315099

C 12.916242 6.765433 2.371070

H 13.717069 8.581629 3.208296

H 11.999647 8.351364 3.515336

C 17.415194 11.825073 1.601871

H 16.006462 12.986629 2.758080

H 16.348170 11.398506 3.432281

C 12.502037 13.833131 -1.811244

H 11.254629 15.019165 -0.514370

H 10.469605 13.568716 -1.121951

C 13.266828 5.885992 3.568000

H 13.687182 6.660780 1.594567

H 11.974675 6.421402 1.921087

H 17.279864 12.379467 0.664841

H 18.302164 12.228202 2.103536

H 17.623573 10.779469 1.342972

H 12.630872 12.796522 -2.143880

H 12.247793 14.429713 -2.694597

H 13.471327 14.186973 -1.436843

H 14.220108 6.191748 4.016951

H 13.356817 4.833463 3.276413

H 12.496051 5.953630 4.345481

Au 10.348726 11.121510 3.447972

C 8.796105 11.341574 4.705868

N 7.894111 11.467843 5.439424

Structure: 2-Bu

Symbol X Y Z

Au 0.298451 3.407533 3.741329

P -2.053236 3.259888 3.563643

C -2.705066 3.540202 1.868413

C -4.219683 3.490807 1.685972

C -2.727603 1.632401 4.074408

C -2.128166 0.467545 3.288807

C -2.978981 4.443032 4.617381

C -2.642999 5.894495 4.280377

H -2.208849 2.796485 1.232364

H -2.307295 4.512189 1.549062

H -4.700613 4.242838 2.326074

H -3.821821 1.645677 3.988693

H -2.494264 1.525712 5.141479

H -1.032165 0.523831 3.335896

H -2.393596 0.559496 2.226829

H -2.680165 4.216971 5.649256

H -4.058718 4.259076 4.546793

H -3.048075 6.150746 3.292078

H -1.552026 6.001330 4.199345

P 1.171875 4.267171 5.805986

C 0.422564 5.862700 6.326762

C 0.950531 6.506716 7.604928

H 0.842954 5.812290 8.449129

H 2.024503 6.712435 7.506082

H 0.537368 6.547388 5.476803

H -0.653831 5.673042 6.411551

C 2.979742 4.574140 5.819228

C 3.393505 5.596332 4.761629

H 2.970870 5.299437 3.792102

H 2.964190 6.578607 5.005275

H 3.309077 4.881727 6.820657

H 3.447594 3.604666 5.603740

C 0.920742 3.145623 7.238325

C -0.553063 2.827252 7.477262

H -1.093827 3.744688 7.748853

H -0.994771 2.476392 6.535301

H 1.471152 2.225637 7.002372

H 1.383164 3.576214 8.135949

C 1.703408 2.877806 2.310067

N 2.525378 2.591628 1.523922

C -2.596274 -0.890676 3.808830

H -3.694013 -0.937010 3.768118

H -2.322947 -0.981807 4.869803

C -2.001475 -2.055590 3.022370

H -2.344920 -3.020039 3.414111

H -2.286465 -2.002878 1.964234

H -0.905699 -2.043949 3.070744

C -0.775334 1.776199 8.562507

H -0.336529 2.128268 9.506617

H -0.231928 0.860259 8.291657

C -2.253748 1.456540 8.767058

H -2.703913 1.068932 7.844490

H -2.397095 0.703083 9.549942

H -2.814942 2.353333 9.058543

C 4.909950 5.730320 4.632259

H 5.336099 6.010932 5.606310

H 5.333459 4.748926 4.378287

C 5.317216 6.753413 3.575564

H 4.926376 6.473812 2.589654

H 4.926707 7.749852 3.819050

H 6.407518 6.832189 3.493862

C 0.218760 7.808935 7.932087

H 0.325238 8.502717 7.086576

H -0.856948 7.603737 8.025613

C 0.730705 8.469638 9.208800

H 0.192072 9.400243 9.421694

H 0.606678 7.805812 10.073644

H 1.797620 8.711960 9.126666

C -3.174245 6.884203 5.315618

H -4.269981 6.811562 5.360393

H -2.808589 6.594590 6.311198

C -2.758399 8.321754 5.019209

H -3.129442 8.646864 4.039299

H -3.148402 9.015632 5.772626

H -1.665750 8.417844 5.009649

C -4.635621 3.734378 0.234661

H -4.156466 2.982789 -0.407997

H -4.248215 4.709750 -0.091600

C -6.148829 3.689670 0.041129

H -6.423804 3.866964 -1.004991

H -6.555180 2.712954 0.332659

H -6.647598 4.452698 0.652054

H -4.605864 2.514685 2.009159

Structure: 3-Bu

Symbol X Y Z

Au 0.407150 3.395408 3.647490

P -1.985370 3.256617 3.564473

C -2.690577 3.511477 1.883592

C -4.206377 3.445653 1.732385

C -2.704068 1.647699 4.084037

C -2.248275 0.502907 3.180478

C -2.887755 4.479712 4.597821

C -2.556616 5.916608 4.199756

H -2.211729 2.759880 1.244738

H -2.314542 4.482622 1.538629

H -4.681130 4.212623 2.359192

H -3.799697 1.710669 4.127157

H -2.350507 1.472715 5.107763

H -1.160230 0.560740 3.051048

H -2.693746 0.617688 2.182149

H -2.570675 4.293221 5.632094

H -3.969610 4.297033 4.556478

H -2.961643 6.129335 3.200614

H -1.466278 6.019546 4.113008

P 1.211685 4.386036 5.690706

C 0.425972 5.936126 6.293956

C 0.907842 6.512425 7.620897

H 0.755126 5.780271 8.425085

H 1.988302 6.705045 7.578050

H 0.548838 6.675656 5.492547

H -0.649319 5.727842 6.339088

C 3.014595 4.741315 5.749876

C 3.400071 5.899847 4.836092

H 2.882072 5.777187 3.878850

H 3.033059 6.846980 5.255741

H 3.336418 4.924943 6.783463

H 3.501897 3.816112 5.415698

C 1.011794 3.207918 7.084648

C -0.449805 2.858117 7.345493

H -0.989374 3.744067 7.710626

H -0.914001 2.579636 6.390887

H 1.559387 2.307075 6.778903

H 1.494425 3.599153 7.989834

C 1.249579 1.432383 3.877962

N 1.712300 0.367913 4.054060

P 1.112623 4.352421 1.564333

C 2.934003 4.340096 1.283633

C 3.480990 5.174040 0.129329

C 0.451680 3.491160 0.074914

C 0.451253 1.971553 0.218754

C 0.575850 6.082507 1.238468

C 1.052902 7.065792 2.299795

H 3.192232 3.279860 1.156770

H 3.404330 4.643326 2.225223

H 3.267236 6.236934 0.306682

H 2.974675 4.910542 -0.809317

H 1.008880 3.807078 -0.817057

H -0.576521 3.852218 -0.053574

H 0.002750 1.706498 1.184276

H 1.483137 1.597288 0.263095

H -0.521856 6.046278 1.238230

H 0.888343 6.397259 0.233943

H 2.143933 7.176506 2.241989

H 0.840968 6.640748 3.289373

C 4.989094 4.994503 -0.047898

H 5.492359 5.228319 0.900742

H 5.203352 3.936964 -0.255898

C 5.560451 5.867534 -1.161509

H 6.640989 5.719945 -1.272362

H 5.090488 5.635452 -2.125583

H 5.387498 6.931452 -0.955426

C 0.402901 8.442503 2.196465

H 0.581570 8.861634 1.196199

H -0.685710 8.331878 2.296897

C 0.925316 9.404460 3.259922

H 0.756109 9.005626 4.268037

H 2.004829 9.565075 3.148248

H 0.431120 10.380926 3.200345

C -0.301202 1.267117 -0.907681

H 0.161174 1.513398 -1.874478

H -1.328439 1.659230 -0.951246

C -0.340169 -0.246953 -0.719408

H 0.672825 -0.667449 -0.699793

H -0.822482 -0.509638 0.229481

H -0.893364 -0.739731 -1.527742

C -2.611897 -0.871411 3.738454

H -3.699134 -0.930891 3.893711

H -2.148901 -0.984799 4.728498

C -2.154014 -2.006993 2.827614

H -2.411962 -2.986807 3.246478

H -2.622185 -1.932409 1.837714

H -1.067031 -1.977202 2.686080

C -0.627249 1.714613 8.341514

H -0.151681 1.982671 9.295713

H -0.093543 0.831090 7.965762

C -2.095485 1.369225 8.575423

H -2.580749 1.062847 7.640289

H -2.206043 0.547419 9.292374

H -2.647385 2.233084 8.967784

C 4.900436 6.006899 4.576180

H 5.432140 6.138984 5.528898

H 5.257918 5.057494 4.152950

C 5.241414 7.152943 3.627257

H 4.746158 7.020083 2.657174

H 4.911326 8.116099 4.036435

H 6.320094 7.217603 3.443844

C 0.181311 7.809837 7.976889

H 0.330897 8.538138 7.167258

H -0.899977 7.616810 8.017625

C 0.648707 8.407697 9.300767

H 0.114158 9.336219 9.532863

H 0.481011 7.708768 10.129823

H 1.721355 8.637140 9.272454

C -3.093886 6.949457 5.188257

H -4.190568 6.885538 5.222982

H -2.742217 6.698085 6.198920

C -2.664316 8.371243 4.841463

H -3.010412 8.656842 3.840194

H -3.066463 9.098969 5.555684

H -1.571838 8.460977 4.852303

C -4.648789 3.640424 0.281797

H -4.172030 2.872964 -0.344231

H -4.275178 4.608532 -0.081448

C -6.164032 3.576075 0.111930

H -6.457025 3.718091 -0.934801

H -6.556033 2.605280 0.440302

H -6.660766 4.352878 0.706909

H -4.577288 2.476927 2.092828

Structure: 6-Bu

Symbol X Y Z

C -1.115316 1.967442 -0.450271

C -1.159873 3.346117 -0.289168

C -2.389923 3.983457 -0.059793

C -3.567882 3.207304 0.031057

C -3.489631 1.813189 -0.122393

C -2.277797 1.188104 -0.368624

H -0.158940 1.488610 -0.644555

H -0.256115 3.944340 -0.342961

H -4.412192 1.244140 -0.045797

H -2.226041 0.111125 -0.497002

C -4.865562 3.847175 0.275838

O -5.923474 3.239236 0.387094

H -4.848693 4.947390 0.360958

O -2.490011 5.316882 0.067675

H -1.591258 5.726234 0.172647

Au -0.315498 5.363680 3.789450

P 1.236145 6.579755 5.161954

C 2.352421 5.550677 6.200101

C 3.431356 6.273645 6.999423

C 2.379137 7.567416 4.118173

C 3.237250 6.681514 3.215736

C 0.568933 7.811145 6.352485

C -0.173528 7.145359 7.509690

H 2.805256 4.817464 5.521025

H 1.699666 4.977231 6.870142

H 2.972004 7.020585 7.660962

H 4.092114 6.827873 6.319588

H 3.002854 8.225037 4.738072

H 1.734606 8.208657 3.502844

H 2.596867 5.924063 2.745387

H 3.975963 6.138551 3.822314

H -0.116143 8.446678 5.776733

H 1.373276 8.459025 6.725225

H 0.543922 6.637292 8.168097

H -0.830609 6.359259 7.114537

P -2.614009 6.005670 4.008922

C -3.222135 6.527202 5.663656

C -4.667180 7.008852 5.752629

H -4.801820 7.897586 5.122049

H -5.347485 6.243262 5.356257

H -3.056090 5.675378 6.336175

H -2.541052 7.315272 6.003042

C -3.837150 4.752022 3.463465

C -3.995145 3.613672 4.467533

H -3.008392 3.351786 4.872256

H -4.590628 3.952410 5.326256

H -4.804657 5.221118 3.244006

H -3.440317 4.364172 2.519386

C -2.972704 7.442324 2.928602

C -2.095201 8.644536 3.267271

H -2.357556 9.032275 4.262657

H -1.051029 8.309206 3.325179

H -2.763791 7.100292 1.908649

H -4.038994 7.698207 2.975252

C 0.036049 6.081028 1.783868

N 0.011538 6.440825 0.664193

P 0.221047 3.041641 3.959401

C -1.003209 1.857595 3.264823

C -0.543467 0.422820 3.023403

C 1.822267 2.588036 3.179297

C 1.859002 2.910111 1.689847

C 0.467498 2.437588 5.682049

C -0.587357 2.963692 6.650963

H -1.390950 2.296357 2.338918

H -1.836894 1.868212 3.976358

H 0.057356 0.056971 3.867810

H 0.112247 0.393045 2.143110

H 2.043729 1.527724 3.361788

H 2.586264 3.174355 3.707424

H 1.527040 3.944135 1.536878

H 1.133938 2.276820 1.164729

H 1.460856 2.788806 5.988218

H 0.503090 1.340108 5.690932

H -1.567386 2.531364 6.407086

H -0.689508 4.046977 6.500749

C 3.958261 7.469485 2.124663

H 4.594765 8.238682 2.585469

H 3.210021 8.002296 1.522244

C 4.798346 6.573514 1.219372

H 4.169199 5.828256 0.718916

H 5.311034 7.153624 0.443293

H 5.561883 6.033362 1.793922

C -0.999498 8.129584 8.335177

H -0.335317 8.896133 8.758534

H -1.694580 8.662951 7.671001

C -1.782985 7.444626 9.450337

H -2.481088 6.705514 9.039481

H -1.111003 6.920237 10.141132

H -2.367007 8.166195 10.033045

C 4.269544 5.310732 7.840604

H 3.605545 4.755286 8.518254

H 4.729110 4.562504 7.179248

C 5.353168 6.020236 8.647652

H 5.940004 5.310685 9.242392

H 6.046290 6.558230 7.988845

H 4.914841 6.752705 9.337159

C -5.074455 7.347322 7.186673

H -4.389677 8.108687 7.585446

H -4.942116 6.456707 7.817188

C -6.513948 7.842863 7.291161

H -7.218742 7.085938 6.924778

H -6.783406 8.077092 8.327645

H -6.662427 8.750832 6.693266

C -2.200817 9.770498 2.241968

H -3.243853 10.111322 2.174222

H -1.937852 9.372095 1.252739

C -1.291256 10.948494 2.580072

H -0.241512 10.631225 2.619761

H -1.371213 11.746797 1.833022

H -1.546172 11.377764 3.557729

C -4.640252 2.371295 3.857845

H -5.623574 2.635076 3.446891

H -4.036958 2.037397 3.003100

C -4.784030 1.235238 4.865550

H -5.228574 0.345100 4.405975

H -3.808144 0.945419 5.276475

H -5.420791 1.532068 5.708584

C 3.239170 2.724246 1.065750

H 3.584079 1.692755 1.227520

H 3.956717 3.375649 1.584332

C 3.240293 3.042980 -0.426956

H 2.568797 2.369095 -0.973879

H 2.895873 4.068402 -0.608644

H 4.241680 2.942074 -0.861311

C -0.262843 2.678314 8.114926

H -0.172404 1.593874 8.270021

H 0.722078 3.105651 8.353809

C -1.313602 3.253744 9.060305

H -2.302886 2.824666 8.857196

H -1.068951 3.050115 10.109294

H -1.393021 4.340662 8.939419

C -1.725551 -0.522286 2.809185

H -2.317099 -0.568046 3.733895

H -2.386625 -0.097161 2.043799

C -1.294747 -1.926574 2.398345

H -2.158221 -2.589599 2.269285

H -0.744244 -1.905680 1.449182

H -0.636855 -2.376877 3.152711

Structure: TS_7/10-Bu_

Symbol X Y Z

C -4.340214 2.762665 -1.702494

C -4.737061 3.997631 -1.195134

C -3.873902 4.769466 -0.417063

C -2.572870 4.302470 -0.099426

C -2.203993 3.043628 -0.618747

C -3.057491 2.288027 -1.411944

H -5.024267 2.181209 -2.315204

H -5.723809 4.401539 -1.406249

H -1.209660 2.679672 -0.379877

H -2.728004 1.324260 -1.792543

C -1.586869 5.042832 0.663432

O -0.451556 4.435230 1.057343

O -4.389195 5.949435 0.047569

H -3.681750 6.550327 0.339996

Au -0.532703 4.849533 3.952702

P 0.843022 6.752898 4.360845

C 2.608529 6.341383 4.674272

C 3.587231 7.497851 4.855478

C 0.888781 8.077621 3.100748

C 1.555609 7.673293 1.788824

C 0.355817 7.669193 5.878209

C -0.068524 6.752914 7.021826

H 2.928121 5.707004 3.840870

H 2.608537 5.698117 5.564092

H 3.278118 8.134948 5.694508

H 3.580666 8.136304 3.962664

H 1.392147 8.951270 3.537598

H -0.155826 8.351552 2.911331

H 1.006723 6.839954 1.334727

H 2.577667 7.313702 1.973851

H -0.476732 8.320678 5.588412

H 1.175505 8.330884 6.184401

H 0.786059 6.148015 7.355760

H -0.814908 6.042082 6.642247

P -2.893944 4.814478 4.243376

C -3.194228 4.058116 5.896678

C -4.632814 4.020087 6.403018

H -5.039314 5.038922 6.452566

H -5.267452 3.466706 5.698595

H -2.776016 3.044428 5.844883

H -2.558769 4.603622 6.605699

C -3.914476 3.825703 3.088826

C -3.317527 2.462853 2.774149

H -2.357473 2.617844 2.267642

H -3.102061 1.922908 3.706626

H -4.922944 3.731882 3.513205

H -4.011313 4.416020 2.171984

C -3.829578 6.388841 4.272246

C -3.339553 7.372434 5.325368

H -3.472637 6.956029 6.334233

H -2.261980 7.513232 5.188944

H -3.699559 6.822542 3.273908

H -4.896632 6.164753 4.402200

C -1.811896 6.334339 1.160136

N -2.015969 7.433202 1.539306

P 0.670000 2.770243 3.825092

C -0.284562 1.207426 4.001994

C 0.503020 -0.078326 4.240781

C 1.654227 2.494501 2.304334

C 2.574874 3.665740 1.976147

C 1.897124 2.685341 5.187327

C 1.232903 2.864943 6.550999

H -0.879875 1.123932 3.085579

H -1.004971 1.366409 4.813815

H 1.031080 -0.022654 5.202071

H 1.274388 -0.202381 3.469154

H 0.922597 2.365016 1.498534

H 2.226309 1.562592 2.401005

H 3.310359 3.802155 2.780995

H 1.974999 4.582442 1.931649

H 2.612294 3.496038 5.010574

H 2.458879 1.743760 5.139701

H 0.577116 2.008017 6.760351

H 0.578204 3.747768 6.518050

H 0.269418 4.654862 0.224123

C -2.419138 6.046413 -2.736319

C -3.494151 6.783990 -3.218530

C -3.631566 8.135496 -2.893408

C -2.674700 8.752937 -2.080683

C -1.593225 8.030702 -1.592395

C -1.448790 6.660999 -1.911592

H -2.319824 4.991895 -2.974246

H -4.239780 6.294296 -3.839809

H -4.479273 8.704134 -3.267084

H -2.776887 9.804069 -1.821637

H -0.854769 8.503849 -0.955619

C -0.373541 5.907642 -1.396001

C 0.653939 5.262330 -1.074356

H 1.635897 5.054893 -1.476327

C 1.606478 8.850122 0.815577

H 2.103112 9.699924 1.306933

H 0.577795 9.174909 0.609326

C 2.323142 8.522781 -0.490152

H 1.827636 7.696501 -1.009772

H 2.337083 9.387803 -1.163865

H 3.363969 8.227690 -0.303679

C -0.654640 7.515448 8.207310

H 0.102917 8.198987 8.615849

H -1.477571 8.146907 7.847705

C -1.166614 6.589773 9.306624

H -1.938632 5.912610 8.919746

H -0.358054 5.971637 9.715206

H -1.604662 7.156356 10.136362

C 5.012831 7.003338 5.103478

H 5.022474 6.364326 5.998001

H 5.320522 6.363116 4.264645

C 6.011755 8.144410 5.273260

H 5.740866 8.782661 6.123687

H 7.025749 7.766677 5.448283

H 6.040561 8.778423 4.378311

C -0.403474 -1.309853 4.241704

H -0.922107 -1.374535 3.275399

H -1.186798 -1.179185 5.001922

C 0.360367 -2.604349 4.505717

H -0.308788 -3.472515 4.499937

H 1.129932 -2.770545 3.741429

H 0.862113 -2.574941 5.481210

C 3.319540 3.482264 0.656461

H 3.967266 2.596184 0.719533

H 2.589856 3.276911 -0.137593

C 4.147989 4.710268 0.291111

H 3.507418 5.594119 0.191393

H 4.894865 4.927915 1.065591

H 4.680236 4.571445 -0.657190

C 2.238185 3.022931 7.689281

H 2.915900 2.157694 7.700923

H 2.867411 3.902747 7.491156

C 1.561922 3.169083 9.049004

H 0.889062 4.034741 9.060228

H 0.962194 2.281865 9.287742

H 2.295782 3.303634 9.851939

C -4.044341 8.724459 5.235307

H -5.127217 8.576552 5.352307

H -3.895634 9.133832 4.226997

C -3.547167 9.722833 6.276633

H -3.703039 9.343887 7.294749

H -2.474429 9.919005 6.155752

H -4.071066 10.682151 6.193756

C -4.224071 1.606040 1.897347

H -5.182526 1.443497 2.412258

H -4.448625 2.152300 0.974089

C -3.586396 0.263780 1.554991

H -2.642216 0.414043 1.017751

H -3.370729 -0.317956 2.460994

H -4.239525 -0.340249 0.914321

C -4.737845 3.374331 7.784946

H -4.334832 2.352691 7.736889

H -4.096832 3.924781 8.488287

C -6.169381 3.339729 8.312669

H -6.823593 2.770646 7.640395

H -6.220402 2.873175 9.303302

H -6.582107 4.352792 8.397939

Structure: TS_12/13-Bu_

Symbol X Y Z

C -3.821841 2.304590 -2.337550

C -4.397123 3.055413 -1.313470

C -3.608383 3.493963 -0.247310

C -2.245559 3.158049 -0.184296

C -1.686007 2.424844 -1.229396

C -2.458054 2.000612 -2.308830

H -4.440007 1.968147 -3.166375

H -5.449491 3.325006 -1.333969

H -0.634848 2.170083 -1.144791

H -2.008760 1.427556 -3.116434

C -1.412176 3.480853 1.030444

O -0.186198 3.178127 1.052141

H -1.994041 3.362688 1.964266

O -4.192922 4.242704 0.727266

H -3.492109 4.798903 1.134784

Au 0.875232 0.639014 0.753687

P 2.144533 1.160676 2.710481

C 3.963349 1.074655 2.456166

C 4.842613 1.322362 3.677925

C 1.845861 2.820680 3.416384

C 2.400843 3.941142 2.538215

C 1.849100 -0.001975 4.099900

C 2.259022 -1.427100 3.731494

H 4.190662 1.802927 1.667405

H 4.172015 0.087025 2.025130

H 4.626567 0.574396 4.452627

H 4.604447 2.300307 4.117043

H 2.254935 2.870830 4.434322

H 0.756626 2.922531 3.477193

H 2.073611 3.775163 1.505899

H 3.500147 3.919666 2.549060

H 0.772306 0.044675 4.307216

H 2.368923 0.336955 5.005309

H 3.353070 -1.490382 3.656800

H 1.873412 -1.665266 2.730205

P -1.102082 -0.633248 0.922563

C -0.807213 -2.159672 1.903137

C -1.976037 -3.127436 2.058161

H -2.818712 -2.621910 2.547896

H -2.336452 -3.446676 1.071264

H 0.049297 -2.662961 1.436249

H -0.457785 -1.824130 2.885854

C -1.802098 -1.249345 -0.654036

C -0.813241 -2.154052 -1.387083

H 0.198522 -1.737508 -1.279445

H -0.784910 -3.142738 -0.908579

H -2.756989 -1.760387 -0.477011

H -2.019818 -0.354302 -1.247839

C -2.510282 0.191171 1.749584

C -2.118846 0.773272 3.105531

H -1.788956 -0.030180 3.779778

H -1.253260 1.434054 2.966420

H -2.836069 0.990253 1.073101

H -3.346567 -0.513563 1.842473

C -1.582601 5.419193 1.080681

N -1.243362 6.538500 1.156112

P 2.156263 0.988850 -1.225065

C 1.370247 0.595105 -2.840204

C 2.150151 0.929187 -4.107996

C 2.861575 2.664159 -1.443344

C 1.779254 3.728344 -1.617071

C 3.632616 -0.102835 -1.149069

C 3.287339 -1.510445 -0.664170

H 0.399110 1.102768 -2.849129

H 1.144279 -0.477119 -2.803847

H 3.143284 0.460563 -4.079897

H 2.318305 2.012241 -4.167561

H 3.567502 2.664182 -2.285112

H 3.440010 2.868081 -0.533036

H 1.092978 3.686829 -0.760983

H 1.177761 3.507865 -2.510770

H 4.320093 0.375436 -0.440010

H 4.142056 -0.127619 -2.120628

H 2.656263 -2.018635 -1.406460

H 2.675930 -1.428370 0.246265

C -1.591865 -4.362931 2.872834

H -1.222182 -4.045057 3.857578

H -0.750936 -4.870600 2.379929

C -2.751774 -5.338857 3.047608

H -2.452098 -6.216432 3.632040

H -3.592970 -4.862509 3.566484

H -3.118835 -5.693066 2.076147

C -3.256787 1.545300 3.770262

H -4.084847 0.857187 3.991809

H -3.654728 2.284603 3.061764

C -2.806202 2.249335 5.046680

H -2.011519 2.974433 4.830867

H -3.633000 2.790880 5.520190

H -2.412263 1.531373 5.777643

C -1.132787 -2.315723 -2.871377

H -2.148547 -2.719176 -2.985453

H -1.142540 -1.323474 -3.343414

C -0.133299 -3.217766 -3.589474

H -0.368506 -3.309776 -4.655912

H 0.887159 -2.821686 -3.506925

H -0.129993 -4.226407 -3.157668

C 4.514487 -2.368792 -0.369272

H 5.112511 -2.486511 -1.283862

H 5.155138 -1.840819 0.351734

C 4.137889 -3.738893 0.188099

H 3.569237 -3.637542 1.120888

H 3.513672 -4.295544 -0.522186

H 5.025589 -4.345695 0.400596

C 1.412847 0.466959 -5.365445

H 1.245524 -0.617674 -5.306413

H 0.416594 0.930743 -5.386475

C 2.165420 0.804684 -6.649209

H 1.617857 0.464262 -7.535645

H 2.315891 1.887251 -6.744203

H 3.154257 0.329180 -6.663567

C 6.332271 1.273151 3.338036

H 6.567286 0.296217 2.891775

H 6.549074 2.024572 2.565668

C 7.223061 1.512518 4.553837

H 7.045265 0.755506 5.328024

H 8.285355 1.474306 4.286370

H 7.025244 2.495515 4.999133

C 1.764819 -2.475626 4.725138

H 0.673857 -2.389483 4.828624

H 2.182789 -2.262407 5.718860

C 2.127725 -3.894849 4.299378

H 1.762059 -4.636867 5.018273

H 1.689934 -4.132690 3.322258

H 3.214916 -4.015989 4.215004

C 1.912442 5.313507 2.995236

H 2.197919 5.471702 4.046089

H 0.816499 5.323812 2.954882

C 2.454892 6.448295 2.133402

H 2.118955 6.339245 1.096768

H 2.102277 7.422458 2.490384

H 3.553098 6.463384 2.135665

C 2.359249 5.134814 -1.743700

H 2.981093 5.343699 -0.861779

H 3.032371 5.180012 -2.612790

C 1.270887 6.197118 -1.868741

H 0.603734 6.183926 -0.999514

H 1.699910 7.202968 -1.948593

H 0.655158 6.023752 -2.760692

Structure: TS_15/16-bra-Bu_

Symbol X Y Z

C -0.223804 5.891992 -3.143144

C -1.466859 6.407292 -2.774819

C -1.987715 6.184180 -1.502096

C -1.257176 5.440632 -0.544391

C -0.008819 4.930964 -0.943060

C 0.507831 5.147335 -2.219120

H 0.163710 6.076562 -4.141641

H -2.059255 6.994913 -3.471276

H 0.561395 4.364720 -0.214612

H 1.479817 4.737981 -2.482815

C -1.767499 5.099984 0.790352

O -0.805442 4.560819 1.672590

H -2.556908 3.683829 0.454463

O -3.230469 6.707361 -1.263347

H -3.455448 6.666614 -0.318484

Au -1.208281 4.621644 4.479931

P 0.091687 6.625612 4.543961

C 1.916489 6.442393 4.415995

C 2.762431 7.688722 4.665436

C -0.320932 7.912013 3.297906

C 0.346779 7.704835 1.937801

C -0.137597 7.507637 6.136516

C -0.050873 6.574108 7.340899

H 2.100805 6.057490 3.407507

H 2.206480 5.635368 5.099911

H 2.724648 7.961865 5.728096

H 2.353432 8.544521 4.111347

H -0.053181 8.896309 3.705795

H -1.411798 7.887950 3.189140

H 0.222585 6.667897 1.607693

H 1.428242 7.874899 2.030685

H -1.123407 7.984579 6.089610

H 0.597760 8.318276 6.210691

H 0.913969 6.046966 7.338327

H -0.819281 5.794441 7.240747

P -3.528569 4.608276 4.985965

C -3.752694 3.749051 6.596664

C -5.185739 3.570788 7.087886

H -5.692487 4.544005 7.135603

H -5.751928 2.962564 6.370149

H -3.253108 2.776966 6.505642

H -3.162241 4.317950 7.327185

C -4.650152 3.738645 3.826582

C -4.297345 2.268200 3.645723

H -3.265584 2.191325 3.289871

H -4.333282 1.750401 4.614741

H -5.680869 3.855994 4.186790

H -4.579232 4.277339 2.874360

C -4.373061 6.220090 5.211387

C -3.881288 6.996297 6.429482

H -4.270466 6.538098 7.349034

H -2.788795 6.921719 6.494319

H -4.179004 6.776001 4.286740

H -5.457435 6.057734 5.267497

C -2.687009 5.933246 1.450582

N -3.519378 6.589631 1.964377

P -0.059763 2.505297 4.486357

C -1.078683 0.978485 4.548284

C -0.360782 -0.332699 4.856507

C 1.125834 2.141443 3.129849

C 1.973212 3.348316 2.740600

C 0.945098 2.446269 6.022328

C 0.060847 2.491655 7.267655

H -1.565104 0.919257 3.567103

H -1.879859 1.156787 5.275325

H 0.123856 -0.279932 5.840880

H 0.438165 -0.506268 4.123943

H 0.518955 1.809021 2.276714

H 1.755763 1.291082 3.421466

H 2.632808 3.625279 3.574037

H 1.305995 4.199745 2.569386

H 1.610687 3.317828 5.998406

H 1.578689 1.549823 6.024263

H -0.561480 1.587101 7.308098

H -0.635244 3.338439 7.188538

C -2.455978 2.495366 0.563402

N -1.910906 1.566137 1.016703

H -0.918181 3.590238 1.688830

C -5.244071 2.906729 8.463646

H -4.729945 1.936213 8.416395

H -4.680145 3.518546 9.182086

C -6.672366 2.709609 8.963642

H -6.689431 2.233239 9.950705

H -7.196912 3.669781 9.046154

H -7.246476 2.075989 8.276127

C -5.218886 1.548011 2.665117

H -6.261578 1.651954 2.998543

H -5.153892 2.041049 1.685675

C -4.861011 0.071833 2.521485

H -5.532300 -0.437484 1.820194

H -3.836379 -0.041017 2.149752

H -4.929027 -0.445618 3.487334

C -4.290601 8.467602 6.382686

H -5.382866 8.532562 6.279694

H -3.870669 8.924244 5.475644

C -3.842556 9.252985 7.611507

H -2.749517 9.258882 7.700954

H -4.176115 10.295933 7.562743

H -4.248001 8.813943 8.531880

C 4.219845 7.479304 4.253801

H 4.624196 6.608378 4.789212

H 4.255526 7.226306 3.185155

C 5.091276 8.702505 4.523508

H 6.129075 8.530889 4.215313

H 4.720256 9.577884 3.975840

H 5.096650 8.956343 5.591046

C -0.217439 8.650437 0.878263

H -0.224095 9.676277 1.276164

H -1.265870 8.385962 0.689165

C 0.570959 8.609848 -0.426579

H 0.130054 9.275043 -1.177799

H 1.610408 8.925737 -0.265925

H 0.585347 7.598908 -0.845743

C -0.226634 7.302507 8.671002

H 0.572338 8.048694 8.783657

H -1.170661 7.863125 8.650215

C -0.225757 6.354580 9.865599

H -0.331879 6.899280 10.810749

H -1.055220 5.640799 9.796180

H 0.705026 5.776259 9.913966

C -1.323066 -1.520970 4.836429

H -1.806190 -1.573746 3.851384

H -2.127977 -1.343268 5.563907

C -0.631649 -2.845669 5.145776

H -1.340746 -3.681342 5.126413

H 0.156213 -3.059165 4.412603

H -0.164899 -2.825546 6.138834

C 0.855537 2.610585 8.565675

H 1.624317 1.825529 8.596395

H 1.392093 3.569542 8.570118

C 2.810498 3.119502 1.484961

H 2.150966 2.782694 0.672958

H 3.528323 2.304808 1.656217

C 3.547110 4.385401 1.054540

H 2.837814 5.194834 0.841512

H 4.225006 4.735299 1.843449

H 4.143303 4.217537 0.150297

C -0.034226 2.505405 9.800713

H -0.518050 1.522029 9.851763

H -0.826056 3.263161 9.780956

H 0.539348 2.645798 10.723974

Structure: PPh_3_

Symbol X Y Z

Au 11.173353 4.378395 11.436230

P 9.130723 3.764076 12.311326

C 8.153479 2.728919 11.189717

C 6.763552 2.857823 11.090963

H 6.243852 3.618540 11.666396

C 6.045804 2.009446 10.247894

H 4.966970 2.114240 10.170538

C 6.710760 1.032893 9.505114

H 6.149298 0.375223 8.846959

C 8.098674 0.905658 9.599651

H 8.619774 0.151995 9.015770

C 8.820744 1.754358 10.435275

H 9.902714 1.665412 10.500829

C 9.267243 2.813249 13.848330

C 10.237950 3.199018 14.782781

H 10.911922 4.021810 14.555572

C 10.341232 2.526002 15.997717

H 11.095593 2.827837 16.719052

C 9.484788 1.459201 16.280597

H 9.571376 0.929911 17.225749

C 8.524500 1.067052 15.347113

H 7.861817 0.233215 15.562636

C 8.412681 1.741904 14.131384

H 7.665552 1.433217 13.405835

C 8.067870 5.176046 12.713063

C 8.060653 6.271884 11.839465

H 8.716784 6.277462 10.972141

C 7.218140 7.353266 12.085884

H 7.216159 8.201261 11.406640

C 6.388121 7.350540 13.209479

H 5.736623 8.198097 13.404745

C 6.401615 6.264853 14.086211

H 5.761726 6.264029 14.964505

C 7.239085 5.176723 13.840708

H 7.249408 4.333945 14.525976

C 12.952160 4.913101 10.676387

N 13.988912 5.225014 10.234953

Structure: 1-Ph

Symbol X Y Z

Au 0.168233 3.141524 3.546852

P -2.207620 3.641872 3.455104

P 1.082658 3.932062 5.613664

C 0.272967 1.031655 3.319819

N 0.305046 -0.131342 3.171563

P 1.128467 4.197985 1.583131

C 2.683873 4.775099 5.435211

C 2.731020 5.902335 4.604498

C 3.863155 4.308425 6.022157

C 3.931943 6.562944 4.373531

H 1.814700 6.253214 4.137538

C 5.071764 4.963342 5.777424

H 3.842411 3.431428 6.662243

C 5.109664 6.089321 4.956251

H 3.956796 7.435223 3.725189

H 5.986574 4.587005 6.228336

H 6.053624 6.590851 4.760790

C 0.072273 5.128283 6.552346

C -1.239399 4.750782 6.869686

C 0.539466 6.378328 6.969545

C -2.068246 5.604493 7.588346

H -1.616882 3.784866 6.547306

C -0.297842 7.240857 7.679263

H 1.556596 6.684186 6.746085

C -1.601524 6.857835 7.989173

H -3.086012 5.299893 7.814503

H 0.074804 8.213514 7.991222

H -2.255046 7.533692 8.534502

C 1.407721 2.650147 6.868342

C 1.611577 1.329329 6.455273

C 1.488648 2.969770 8.231229

C 1.899894 0.338076 7.394298

H 1.524929 1.074548 5.403116

C 1.773101 1.977546 9.166834

H 1.325188 3.993086 8.558395

C 1.980393 0.659744 8.749416

H 2.050064 -0.686214 7.063153

H 1.830939 2.231522 10.222341

H 2.197507 -0.114162 9.481391

C 0.491652 5.872085 1.262557

C -0.793091 6.003015 0.713947

C 1.156609 7.022689 1.705553

C -1.406276 7.249362 0.635544

H -1.318011 5.125485 0.353785

C 0.538626 8.271183 1.629877

H 2.163990 6.946829 2.099869

C -0.747648 8.388124 1.102643

H -2.406970 7.324751 0.218753

H 1.067740 9.153086 1.982606

H -1.232116 9.359695 1.052983

C 2.943511 4.355660 1.618355

C 3.652721 3.566044 2.528931

C 3.642097 5.184560 0.731595

C 5.044851 3.610825 2.562451

H 3.103518 2.925491 3.214086

C 5.033441 5.240292 0.776359

H 3.100328 5.787932 0.007884

C 5.736170 4.453318 1.692489

H 5.585618 2.999980 3.279546

H 5.570362 5.892410 0.091860

H 6.821785 4.498916 1.726779

C 0.863369 3.325860 -0.001464

C 0.752192 3.995429 -1.225986

C 0.818220 1.926993 0.023930

C 0.576982 3.274654 -2.406106

H 0.787966 5.080344 -1.261293

C 0.654536 1.207702 -1.158776

H 0.889778 1.401250 0.972019

C 0.527046 1.879486 -2.375643

H 0.479641 3.804301 -3.350631

H 0.614669 0.122002 -1.123806

H 0.386978 1.319242 -3.296734

C -2.875660 3.630715 1.761213

C -3.779002 4.587430 1.285157

C -2.366720 2.663508 0.883284

C -4.152690 4.587288 -0.059369

H -4.162807 5.352400 1.953810

C -2.737691 2.667429 -0.458159

H -1.650800 1.933002 1.249405

C -3.625807 3.635447 -0.934259

H -4.843894 5.342143 -0.426310

H -2.308889 1.933618 -1.134501

H -3.899949 3.651839 -1.985994

C -2.730696 5.258003 4.108829

C -3.990919 5.494418 4.668933

C -1.802733 6.304614 4.037029

C -4.320734 6.764658 5.140203

H -4.710620 4.685359 4.753479

C -2.134177 7.572389 4.504154

H -0.814870 6.113468 3.628173

C -3.394332 7.805132 5.056761

H -5.299045 6.938669 5.581555

H -1.401237 8.372184 4.449188

H -3.648721 8.791645 5.436054

C -3.235518 2.411270 4.318654

C -2.645688 1.637875 5.326166

C -4.584268 2.207726 3.996676

C -3.403004 0.703453 6.030947

H -1.582833 1.744388 5.528840

C -5.339468 1.269620 4.698458

H -5.039641 2.771804 3.186426

C -4.751905 0.522269 5.722159

H -2.932827 0.105132 6.806880

H -6.384544 1.116307 4.441316

H -5.340625 -0.212865 6.264868

Structure: 2-Ph

Symbol X Y Z

Au -0.139911 3.041300 3.356767

P -2.360399 3.921451 3.262362

C 0.592735 1.839451 4.852548

N 1.009149 1.149841 5.704001

P 1.062117 3.805500 1.432520

C 0.766019 5.565361 1.094868

C -0.390202 5.976508 0.416953

C 1.594394 6.530884 1.685181

C -0.707071 7.329684 0.327127

H -1.050226 5.241426 -0.030869

C 1.270644 7.884148 1.595557

H 2.487778 6.223984 2.222205

C 0.118950 8.287182 0.917436

H -1.610365 7.631188 -0.195844

H 1.918794 8.622913 2.059836

H -0.135771 9.341768 0.853840

C 2.877391 3.673393 1.504645

C 3.469734 3.080359 2.623414

C 3.682507 4.144413 0.457559

C 4.859218 2.958822 2.696380

H 2.841177 2.713183 3.431159

C 5.066865 4.022834 0.533132

H 3.227741 4.609550 -0.413111

C 5.656712 3.428861 1.653920

H 5.313162 2.496103 3.568578

H 5.687574 4.389655 -0.280331

H 6.738058 3.334071 1.710632

C 0.614745 2.924084 -0.094422

C 0.648700 3.516020 -1.362310

C 0.237280 1.580285 0.027831

C 0.295319 2.776888 -2.489946

H 0.934059 4.558218 -1.471680

C -0.107635 0.840007 -1.101180

H 0.204552 1.120158 1.013506

C -0.084497 1.439517 -2.361895

H 0.314623 3.247325 -3.469627

H -0.402453 -0.200592 -0.994886

H -0.363471 0.866421 -3.242296

C -2.973764 4.183203 1.570481

C -3.605222 5.361888 1.160852

C -2.716909 3.174977 0.631796

C -3.958470 5.534508 -0.178199

H -3.796189 6.153839 1.879143

C -3.065659 3.349269 -0.704072

H -2.214218 2.264431 0.944528

C -3.681329 4.535597 -1.112994

H -4.439162 6.457323 -0.493011

H -2.833947 2.571114 -1.425670

H -3.939472 4.682039 -2.158590

C -2.503205 5.557463 4.039344

C -3.597001 5.922371 4.832651

C -1.476859 6.483796 3.806668

C -3.666808 7.204860 5.377977

H -4.391579 5.207517 5.025909

C -1.555862 7.766327 4.342646

H -0.620006 6.202789 3.202240

C -2.650678 8.129327 5.130478

H -4.517070 7.480619 5.996458

H -0.757656 8.477375 4.148365

H -2.708238 9.127817 5.556125

C -3.649569 2.910747 4.048330

C -3.294344 2.132570 5.157608

C -4.974474 2.909179 3.593153

C -4.261443 1.375667 5.817455

H -2.259222 2.112859 5.490423

C -5.936764 2.143518 4.250224

H -5.251576 3.502060 2.725515

C -5.582314 1.379804 5.364594

H -3.979197 0.773382 6.676823

H -6.962865 2.142411 3.891415

H -6.333397 0.781436 5.873828

Structure: 3-Ph

Symbol X Y Z

Au -0.165637 2.898678 3.370046

P -2.323271 4.002175 3.376638

P 1.017062 3.825527 5.270695

C -0.292914 0.806059 3.398619

N -0.364538 -0.364785 3.415086

P 0.983397 3.777655 1.427950

C 1.780927 5.456054 5.016967

C 0.995724 6.615087 5.085994

C 3.119429 5.564452 4.612750

C 1.548608 7.860092 4.797731

H -0.049609 6.546805 5.364834

C 3.665460 6.810835 4.310281

H 3.736605 4.676989 4.523587

C 2.885975 7.964751 4.411871

H 0.923322 8.746220 4.861755

H 4.702019 6.874141 3.990279

H 3.314865 8.936413 4.180287

C -0.115900 4.039619 6.687885

C -1.171573 3.125234 6.789412

C 0.018790 5.038871 7.658262

C -2.096270 3.220158 7.826804

H -1.275672 2.350894 6.035350

C -0.912129 5.140911 8.691132

H 0.835775 5.752052 7.599699

C -1.974420 4.237532 8.774754

H -2.920413 2.513277 7.876516

H -0.809868 5.931383 9.430513

H -2.703900 4.327971 9.575587

C 2.359766 2.816293 5.985664

C 2.448263 1.468902 5.621956

C 3.269951 3.342868 6.912107

C 3.446948 0.659119 6.164080

H 1.732707 1.055158 4.915752

C 4.270604 2.535352 7.447380

H 3.203586 4.386572 7.207561

C 4.363051 1.192182 7.070914

H 3.507602 -0.386238 5.872582

H 4.978120 2.952544 8.159639

H 5.146223 0.563932 7.487721

C 0.547918 5.476320 0.948089

C -0.449063 5.717709 -0.008135

C 1.107731 6.562062 1.635556

C -0.856468 7.021206 -0.287656

H -0.916996 4.890136 -0.530322

C 0.709491 7.863717 1.342629

H 1.855818 6.392187 2.401541

C -0.271454 8.100106 0.378094

H -1.636740 7.188170 -1.025345

H 1.159652 8.690996 1.884119

H -0.586867 9.116228 0.155126

C 2.788580 3.805282 1.708222

C 3.309711 2.805414 2.538628

C 3.657122 4.744878 1.141643

C 4.672397 2.757512 2.823457

H 2.635048 2.076869 2.977967

C 5.019647 4.703789 1.434241

H 3.271578 5.522611 0.488988

C 5.529663 3.715738 2.279874

H 5.054555 1.985836 3.486664

H 5.684396 5.448677 1.003830

H 6.590987 3.694498 2.513956

C 0.837756 2.828581 -0.124365

C 1.244070 3.357298 -1.357002

C 0.343522 1.521850 -0.063931

C 1.133116 2.594335 -2.516926

H 1.637735 4.368771 -1.412058

C 0.239059 0.756036 -1.225684

H 0.043543 1.105050 0.894334

C 0.626501 1.292880 -2.453458

H 1.443135 3.013484 -3.470948

H -0.148366 -0.257830 -1.167347

H 0.538301 0.699620 -3.360120

C -2.972149 4.151351 1.675294

C -3.800008 5.191788 1.238949

C -2.611318 3.140956 0.775448

C -4.227161 5.236626 -0.087440

H -4.096119 5.979812 1.925151

C -3.045987 3.179503 -0.547390

H -1.968549 2.334289 1.114789

C -3.846830 4.235827 -0.984819

H -4.855615 6.058223 -0.422140

H -2.738442 2.396460 -1.235619

H -4.173426 4.280860 -2.020660

C -2.350942 5.691369 4.049244

C -2.655031 5.919246 5.399184

C -1.929884 6.767633 3.256041

C -2.564548 7.202949 5.934905

H -2.954077 5.095391 6.038198

C -1.855229 8.051117 3.790764

H -1.659968 6.605093 2.218713

C -2.175026 8.275846 5.130762

H -2.795735 7.358736 6.985215

H -1.531128 8.872071 3.157334

H -2.109839 9.277112 5.548986

C -3.686521 3.157676 4.248647

C -3.522720 1.814285 4.600829

C -4.896387 3.804365 4.535170

C -4.549944 1.127927 5.249478

H -2.590806 1.306841 4.364245

C -5.918539 3.120161 5.188598

H -5.036178 4.845655 4.257285

C -5.745158 1.781024 5.550604

H -4.410814 0.084590 5.520616

H -6.851542 3.630382 5.414691

H -6.542970 1.249286 6.062981

Structure: 6-Ph

Symbol X Y Z

Au -0.195348 2.890504 3.347139

P -2.337952 3.998756 3.370239

P 1.008042 3.829824 5.256863

C -0.170758 0.797404 3.349255

N 0.002339 -0.363550 3.319180

P 1.008696 3.743283 1.424560

C 1.757173 5.467710 4.997591

C 0.962180 6.619076 5.074132

C 3.095193 5.592288 4.596237

C 1.502463 7.870771 4.790233

H -0.080325 6.540296 5.359358

C 3.629376 6.845090 4.300308

H 3.722675 4.713223 4.505763

C 2.838306 7.990669 4.404712

H 0.869068 8.750531 4.860088

H 4.666293 6.919186 3.983806

H 3.257642 8.967639 4.178056

C -0.120589 4.040554 6.678002

C -1.176109 3.127133 6.783621

C 0.021100 5.036659 7.650727

C -2.095266 3.219843 7.826139

H -1.284513 2.354450 6.028767

C -0.903913 5.136766 8.688924

H 0.839630 5.747854 7.590196

C -1.966983 4.234489 8.775990

H -2.919458 2.513213 7.878999

H -0.795946 5.924333 9.430521

H -2.691532 4.323319 9.581422

C 2.362419 2.830377 5.963102

C 2.433086 1.474660 5.628751

C 3.310480 3.372257 6.841399

C 3.449915 0.672690 6.144134

H 1.695530 1.045565 4.957794

C 4.333221 2.572717 7.347249

H 3.260452 4.421694 7.118547

C 4.408905 1.222300 6.994389

H 3.488408 -0.375870 5.866639

H 5.073640 3.003993 8.016292

H 5.213360 0.602352 7.381499

C 0.579732 5.438839 0.929425

C -0.412948 5.662283 -0.035612

C 1.130351 6.534049 1.608341

C -0.833451 6.959270 -0.325435

H -0.867090 4.825116 -0.555481

C 0.719924 7.829460 1.303667

H 1.879801 6.376412 2.375627

C -0.262410 8.048376 0.336330

H -1.611395 7.113994 -1.068220

H 1.161855 8.665726 1.838104

H -0.587971 9.059583 0.105805

C 2.803434 3.767250 1.747303

C 3.293474 2.819823 2.653412

C 3.692974 4.671620 1.155431

C 4.643313 2.787947 2.989652

H 2.605505 2.116499 3.107294

C 5.046033 4.640015 1.490732

H 3.330592 5.418762 0.455082

C 5.522178 3.704479 2.412640

H 4.998283 2.057159 3.709616

H 5.728722 5.354705 1.037708

H 6.575842 3.690231 2.679251

C 0.862300 2.808058 -0.135216

C 1.454293 3.267829 -1.319411

C 0.177774 1.590720 -0.137986

C 1.356775 2.518199 -2.488898

H 1.990352 4.212953 -1.327454

C 0.080203 0.837635 -1.308357

H -0.258294 1.223808 0.786671

C 0.668270 1.300859 -2.485040

H 1.820247 2.879695 -3.403267

H -0.447189 -0.112623 -1.295256

H 0.596512 0.714638 -3.397485

C -3.029132 4.185354 1.688967

C -3.852327 5.245572 1.292804

C -2.712848 3.184372 0.764079

C -4.317821 5.317120 -0.019335

H -4.115652 6.027857 1.998359

C -3.184972 3.249100 -0.544646

H -2.076042 2.362941 1.075084

C -3.980920 4.324367 -0.942467

H -4.942740 6.153426 -0.322571

H -2.912839 2.471071 -1.253157

H -4.337723 4.390089 -1.967125

C -2.349687 5.682307 4.056289

C -2.669862 5.916504 5.400457

C -1.915694 6.752554 3.261504

C -2.585387 7.203945 5.929165

H -2.977065 5.096388 6.040372

C -1.849636 8.039374 3.788100

H -1.630608 6.582221 2.229172

C -2.187911 8.271769 5.122487

H -2.828837 7.366739 6.975587

H -1.516752 8.856399 3.154172

H -2.129413 9.275730 5.535087

C -3.684108 3.141794 4.255954

C -3.505882 1.800494 4.608474

C -4.897911 3.777163 4.550508

C -4.520982 1.104575 5.265568

H -2.568787 1.304709 4.367081

C -5.908514 3.084034 5.212328

H -5.050070 4.816393 4.271579

C -5.719947 1.747073 5.574508

H -4.370342 0.063197 5.537995

H -6.844547 3.585552 5.444834

H -6.508777 1.208601 6.093538

C 3.410833 0.424973 0.271907

C 2.663999 -0.173542 1.277429

C 3.299743 -0.702327 2.410523

C 4.706983 -0.624279 2.511723

C 5.438844 -0.015133 1.477685

C 4.806317 0.508065 0.362422

H 2.896340 0.838331 -0.590716

H 1.584260 -0.229450 1.205247

H 6.518433 0.038055 1.588533

H 5.380481 0.985915 -0.425873

C 5.403839 -1.157263 3.683956

O 6.606672 -1.034531 3.889854

H 4.775459 -1.702769 4.409024

O 2.608148 -1.282756 3.409068

H 1.637299 -1.086189 3.321905

Structure: TS_7/10-Ph_

Symbol X Y Z

Au -0.093888 3.284883 3.342942

P -2.378880 3.903777 3.341015

P 1.088581 3.818354 5.339454

P 1.087024 3.829331 1.364573

C 1.829813 5.456338 5.082045

C 1.020513 6.597512 5.168396

C 3.156254 5.584939 4.649259

C 1.543953 7.852768 4.871980

H -0.020277 6.505252 5.461515

C 3.672280 6.843288 4.341041

H 3.783768 4.706458 4.538964

C 2.872860 7.981401 4.462475

H 0.904763 8.728096 4.947872

H 4.700121 6.928066 3.999176

H 3.279285 8.961247 4.225271

C 0.010527 3.994374 6.798916

C -1.038729 3.075638 6.914227

C 0.176683 4.972921 7.784800

C -1.924904 3.139649 7.986003

H -1.168863 2.319986 6.146754

C -0.717547 5.045864 8.852515

H 0.988691 5.690664 7.712410

C -1.771300 4.134659 8.953359

H -2.742989 2.426827 8.050435

H -0.592338 5.818977 9.606435

H -2.470433 4.201468 9.782946

C 2.444704 2.758203 5.913373

C 2.520617 1.458807 5.404732

C 3.388586 3.197330 6.851892

C 3.544455 0.604275 5.811697

H 1.776684 1.122352 4.689731

C 4.411669 2.343353 7.255928

H 3.331603 4.205258 7.255057

C 4.492571 1.048031 6.733045

H 3.598525 -0.395425 5.390296

H 5.148666 2.687191 7.977344

H 5.297160 0.387571 7.046491

C 0.582130 5.521513 0.935021

C -0.439732 5.741205 0.000985

C 1.098502 6.607037 1.655215

C -0.914976 7.031914 -0.227363

H -0.874918 4.907212 -0.539634

C 0.631536 7.896097 1.412593

H 1.860633 6.445893 2.410066

C -0.373724 8.114524 0.468631

H -1.713614 7.185904 -0.947872

H 1.046637 8.726901 1.976204

H -0.742621 9.120503 0.285568

C 2.887032 3.876436 1.627565

C 3.422321 2.847601 2.413042

C 3.734225 4.862364 1.111171

C 4.783650 2.814180 2.701173

H 2.768760 2.076728 2.809037

C 5.096077 4.835419 1.410836

H 3.333364 5.662164 0.495186

C 5.621632 3.817583 2.210017

H 5.178714 2.014557 3.321839

H 5.747518 5.613963 1.021795

H 6.682195 3.806449 2.448003

C 0.861668 2.872974 -0.159345

C 1.271952 3.356775 -1.409148

C 0.266281 1.613696 -0.063500

C 1.059810 2.589144 -2.551564

H 1.743236 4.333052 -1.490104

C 0.054096 0.844402 -1.206828

H -0.030295 1.232386 0.907209

C 0.446112 1.335673 -2.451617

H 1.372000 2.967470 -3.521707

H -0.418501 -0.129246 -1.113662

H 0.278344 0.742486 -3.347022

C -3.061183 4.086612 1.662604

C -3.915544 5.123111 1.270762

C -2.676497 3.119829 0.725880

C -4.353787 5.201575 -0.050906

H -4.220942 5.881809 1.985701

C -3.124748 3.190328 -0.589996

H -2.010863 2.317467 1.025667

C -3.957040 4.240072 -0.983470

H -5.003395 6.018847 -0.354049

H -2.804034 2.436765 -1.304418

H -4.294398 4.311401 -2.014312

C -2.440759 5.560250 4.085238

C -2.759355 5.725755 5.439416

C -2.007500 6.665757 3.340363

C -2.667266 6.984800 6.031810

H -3.066264 4.873503 6.036641

C -1.935321 7.924137 3.931066

H -1.721178 6.542927 2.301027

C -2.266207 8.089218 5.277705

H -2.906581 7.097152 7.085754

H -1.602505 8.771727 3.338659

H -2.199716 9.070559 5.740403

C -3.631146 2.939535 4.236848

C -3.287482 1.671064 4.711522

C -4.930270 3.426213 4.435778

C -4.227223 0.892380 5.386255

H -2.285738 1.289482 4.542640

C -5.866039 2.652400 5.117415

H -5.206381 4.410144 4.065182

C -5.515282 1.384800 5.593816

H -3.948047 -0.095595 5.742481

H -6.871180 3.034860 5.275256

H -6.249695 0.782440 6.122223

C -2.790286 -1.812030 0.121400

C -1.511452 -2.365331 0.108895

C -0.517191 -1.917089 0.979979

C -0.786834 -0.874341 1.906001

C -2.093168 -0.341998 1.898978

C -3.079772 -0.790023 1.029415

H -3.547636 -2.178660 -0.566694

H -1.252627 -3.165217 -0.580217

H -2.312005 0.456547 2.596996

H -4.067708 -0.336824 1.059671

C 0.177853 -0.333713 2.838638

O -0.161802 0.726260 3.615501

O 0.702168 -2.508097 0.830402

H 1.309164 -2.263212 1.549469

C 1.542545 -0.647940 2.813104

N 2.685547 -0.936688 2.825560

H -0.429014 0.287219 4.589388

C -1.012598 -3.681672 3.448570

C -0.696453 -4.851984 2.769176

C 0.596191 -5.377356 2.825349

C 1.578675 -4.726312 3.579066

C 1.278664 -3.558872 4.269421

C -0.025478 -3.015750 4.210103

H -2.010624 -3.258594 3.390399

H -1.460408 -5.346566 2.174820

H 0.839792 -6.286328 2.281579

H 2.587168 -5.129982 3.623196

H 2.039081 -3.045281 4.848169

C -0.339024 -1.819225 4.889800

C -0.663324 -0.824871 5.577219

H -1.023142 -0.565643 6.562362

Structure: TS_12/13-Ph_

Symbol X Y Z

Au 0.013044 3.280937 2.975101

P -2.270862 3.995294 3.109823

P 1.131753 3.571771 5.036879

P 1.119590 4.038160 1.025194

C 1.857299 5.237559 4.949963

C 1.051956 6.372470 5.106983

C 3.197329 5.393037 4.568708

C 1.589574 7.644069 4.922863

H 0.005255 6.265702 5.369682

C 3.726191 6.666878 4.366988

H 3.826167 4.521490 4.418619

C 2.926943 7.796804 4.553568

H 0.952799 8.514194 5.054726

H 4.763949 6.772598 4.062279

H 3.342074 8.789806 4.401354

C -0.040662 3.592673 6.430232

C -1.077821 2.652674 6.381419

C 0.032494 4.479165 7.510199

C -2.039527 2.603379 7.386916

H -1.148227 1.976646 5.534827

C -0.935723 4.436744 8.512902

H 0.826312 5.219024 7.559142

C -1.973820 3.503970 8.451978

H -2.848356 1.880700 7.319516

H -0.883139 5.139386 9.340670

H -2.732350 3.483611 9.230181

C 2.507444 2.540865 5.627121

C 2.839228 1.380242 4.927566

C 3.237596 2.893081 6.771392

C 3.896637 0.575340 5.352824

H 2.272781 1.101067 4.046820

C 4.293777 2.092173 7.196021

H 2.986004 3.794947 7.323302

C 4.625618 0.933270 6.485565

H 4.137158 -0.325655 4.796614

H 4.859542 2.369369 8.081827

H 5.451015 0.310347 6.820525

C 0.602512 5.761493 0.767821

C -0.432657 6.080178 -0.120356

C 1.147631 6.766404 1.579081

C -0.894837 7.392699 -0.214288

H -0.887949 5.306303 -0.729546

C 0.692721 8.077478 1.471639

H 1.924985 6.524056 2.296873

C -0.326845 8.396599 0.572052

H -1.704255 7.624738 -0.901006

H 1.127848 8.845967 2.104376

H -0.686086 9.419541 0.493816

C 2.932912 4.113612 1.194099

C 3.541872 3.104613 1.947796

C 3.726260 5.109886 0.612923

C 4.922477 3.087131 2.128182

H 2.925341 2.339875 2.407438

C 5.107265 5.101701 0.804576

H 3.268120 5.904414 0.030723

C 5.707589 4.093077 1.562771

H 5.375338 2.298639 2.723657

H 5.716238 5.886629 0.363194

H 6.784012 4.094783 1.713896

C 0.842996 3.216742 -0.571433

C 1.257858 3.799432 -1.776796

C 0.216132 1.966102 -0.580601

C 1.030854 3.141320 -2.982580

H 1.746938 4.770101 -1.774195

C -0.002016 1.306255 -1.791051

H -0.095931 1.508418 0.357389

C 0.398860 1.893685 -2.990609

H 1.347013 3.599204 -3.916393

H -0.488065 0.334058 -1.790868

H 0.222834 1.381677 -3.933228

C -2.991945 4.326145 1.468597

C -3.844405 5.397081 1.181536

C -2.648762 3.426732 0.452150

C -4.312808 5.584807 -0.118669

H -4.129271 6.095682 1.962799

C -3.125337 3.607920 -0.843435

H -1.993063 2.592464 0.680239

C -3.949601 4.697028 -1.133844

H -4.961234 6.428997 -0.339536

H -2.834550 2.910318 -1.624475

H -4.309482 4.853488 -2.147534

C -2.321100 5.577299 3.999306

C -2.591114 5.634408 5.373208

C -1.928247 6.745603 3.330561

C -2.503796 6.847458 6.055640

H -2.855839 4.734313 5.917277

C -1.852942 7.956037 4.013301

H -1.674346 6.708861 2.276513

C -2.145889 8.013692 5.377402

H -2.710510 6.873637 7.122047

H -1.549725 8.851102 3.477314

H -2.081974 8.958353 5.911324

C -3.492220 2.913564 3.906505

C -3.223757 1.539361 3.913682

C -4.687185 3.395847 4.455511

C -4.134370 0.653311 4.489032

H -2.307892 1.170071 3.454089

C -5.587716 2.509045 5.042061

H -4.911497 4.458898 4.433114

C -5.309935 1.139079 5.063409

H -3.916353 -0.410877 4.476063

H -6.509391 2.886320 5.478239

H -6.015946 0.450357 5.520733

C 3.835951 -1.832332 2.200139

C 3.072028 -2.358931 3.239321

C 1.812817 -1.821980 3.521266

C 1.307941 -0.756875 2.753462

C 2.080201 -0.262441 1.699038

C 3.340729 -0.783910 1.419638

H 4.820026 -2.247705 1.996841

H 3.434369 -3.185283 3.844620

H 1.681830 0.564194 1.121241

H 3.933223 -0.371160 0.607745

C 0.044943 -0.041096 3.132150

O -0.440837 0.832602 2.352004

H -0.025611 0.136222 4.221814

O 1.108999 -2.344221 4.556742

H 0.154859 -2.156740 4.395702

C -1.282174 -1.530773 3.263212

N -2.324681 -2.052762 3.143828

Structure: TS_15/16-bra-Ph_

Symbol X Y Z

C 2.292762 4.590054 -1.534699

C 1.149727 5.246423 -1.988785

C 0.057678 5.452263 -1.146127

C 0.096132 5.020985 0.200655

C 1.263239 4.370590 0.631446

C 2.345860 4.144224 -0.216016

H 3.128818 4.429887 -2.210520

H 1.076489 5.603765 -3.012419

H 1.313793 4.049354 1.664558

H 3.225518 3.629490 0.161305

C -1.053332 5.153130 1.124309

O -0.725668 4.894061 2.479733

H -1.889412 3.985700 0.938751

O -1.020393 6.071168 -1.714105

H -1.704418 6.276164 -1.055101

C -1.902584 6.295024 1.011015

N -2.685362 7.159220 0.885464

C -2.474275 2.800980 1.149516

N -2.991462 1.753631 1.132509

H -1.352705 4.224137 2.816605

Au 1.580712 5.136760 4.358152

P -0.363389 5.586640 5.675068

P 2.765144 3.103722 4.360341

P 2.675858 7.187431 3.777659

C 4.385927 3.197690 3.547914

C 5.345687 4.068251 4.085906

C 4.678790 2.489659 2.379432

C 6.593384 4.196446 3.488558

H 5.111147 4.639920 4.980058

C 5.926361 2.636091 1.769112

H 3.939589 1.820860 1.948156

C 6.886540 3.478507 2.326529

H 7.327628 4.875203 3.912786

H 6.145031 2.085395 0.857842

H 7.856204 3.590506 1.849127

C 3.154080 2.612148 6.074497

C 2.131917 2.718484 7.025132

C 4.393207 2.084176 6.456604

C 2.339719 2.307747 8.338586

H 1.172917 3.135797 6.739504

C 4.603560 1.679689 7.775150

H 5.195520 1.990662 5.730917

C 3.579887 1.791205 8.717889

H 1.537255 2.409282 9.063367

H 5.569958 1.274569 8.064226

H 3.749445 1.478704 9.745024

C 1.965445 1.620698 3.686437

C 0.744932 1.729065 3.014145

C 2.548230 0.357753 3.869970

C 0.109253 0.589475 2.519908

H 0.286211 2.703084 2.880766

C 1.915170 -0.778439 3.375118

H 3.490601 0.264822 4.403713

C 0.695375 -0.662456 2.700296

H -0.839670 0.691227 2.001444

H 2.369203 -1.755417 3.519437

H 0.200663 -1.551571 2.318026

C 2.982350 7.946425 5.395335

C 2.037322 8.814163 5.959730

C 4.058298 7.501618 6.178669

C 2.181633 9.246055 7.276721

H 1.179692 9.139594 5.379950

C 4.200885 7.939942 7.493581

H 4.782928 6.808352 5.761250

C 3.263496 8.815895 8.045339

H 1.435624 9.910972 7.702849

H 5.040396 7.590184 8.088753

H 3.370315 9.151658 9.073281

C 4.274776 7.072457 2.920081

C 4.317929 6.200608 1.826047

C 5.375432 7.887440 3.203308

C 5.442276 6.154775 1.009444

H 3.463539 5.567017 1.609192

C 6.514966 7.814938 2.401389

H 5.344759 8.582652 4.037621

C 6.547135 6.956310 1.300947

H 5.459643 5.479549 0.158942

H 7.373071 8.442740 2.628321

H 7.433975 6.910310 0.674129

C 1.841214 8.434530 2.755998

C 2.062854 9.808592 2.902744

C 1.038661 7.966854 1.711473

C 1.453375 10.704933 2.027159

H 2.704879 10.180509 3.696282

C 0.447665 8.863035 0.824190

H 0.881210 6.899928 1.591890

C 0.646633 10.234228 0.987820

H 1.614602 11.772719 2.151622

H -0.177100 8.490296 0.018903

H 0.174301 10.936434 0.305917

C -0.960899 7.291170 5.517943

C -1.202861 8.115726 6.621643

C -1.093374 7.804654 4.221374

C -1.554119 9.451523 6.423860

H -1.094856 7.725477 7.629244

C -1.447998 9.135305 4.027017

H -0.908312 7.157565 3.371331

C -1.668214 9.964342 5.129902

H -1.730574 10.094130 7.282757

H -1.532811 9.521555 3.015784

H -1.928868 11.008957 4.980646

C -0.028324 5.386670 7.446820

C -0.936820 4.777579 8.318892

C 1.196240 5.858935 7.937752

C -0.622461 4.648677 9.672813

H -1.884283 4.402912 7.941555

C 1.499133 5.743990 9.290475

H 1.913902 6.307092 7.257070

C 0.591223 5.135937 10.160596

H -1.328882 4.169874 10.345970

H 2.450688 6.115583 9.660340

H 0.833206 5.033685 11.215330

C -1.792199 4.529388 5.320582

C -1.567798 3.155394 5.144814

C -3.078194 5.048512 5.139003

C -2.616245 2.312562 4.789419

H -0.564634 2.752581 5.252944

C -4.125912 4.200215 4.777703

H -3.256139 6.113497 5.254605

C -3.897118 2.836134 4.599416

H -2.425868 1.256258 4.624317

H -5.118526 4.612088 4.617043

H -4.707039 2.184049 4.286162
